# Intraneuronal sortilin aggregation relative to granulovacuolar degeneration, tau pathogenesis and sorfra plaque formation in human hippocampal formation

**DOI:** 10.3389/fnagi.2022.926904

**Published:** 2022-08-01

**Authors:** Juan Jiang, Chen Yang, Jia-Qi Ai, Qi-Lei Zhang, Xiao-Lu Cai, Tian Tu, Lily Wan, Xiao-Sheng Wang, Hui Wang, Aihua Pan, Jim Manavis, Wei-Ping Gai, Chong Che, Ewen Tu, Xiao-Ping Wang, Zhen-Yan Li, Xiao-Xin Yan

**Affiliations:** ^1^Department of Anatomy and Neurobiology, Central South University Xiangya School of Medicine, Changsha, China; ^2^Department of Neurology, Xiangya Hospital, Changsha, China; ^3^Faculty of Health and Medical Sciences, The University of Adelaide, Adelaide, SA, Australia; ^4^GeneScience Pharmaceuticals Co., Ltd., Changchun High-Tech Dev. Zone, Changchun, China; ^5^Department of Neurology, Brain Hospital of Hunan Province, Changsha, China; ^6^Department of Psychiatry, The Second Xiangya Hospital, Changsha, China; ^7^Department of Neurosurgery, Xiangya Hospital, Changsha, China

**Keywords:** brain aging, dementia, neurodegeneration, neuritic plaque, proteostasis, Vps10p

## Abstract

Extracellular β-amyloid (Aβ) deposition and intraneuronal phosphorylated-tau (pTau) accumulation are the hallmark lesions of Alzheimer’s disease (AD). Recently, “sorfra” plaques, named for the extracellular deposition of *sor*tilin c-terminal *fra*gments, are reported as a new AD-related proteopathy, which develop in the human cerebrum resembling the spatiotemporal trajectory of tauopathy. Here, we identified intraneuronal sortilin aggregation as a change related to the development of granulovacuolar degeneration (GVD), tauopathy, and sorfra plaques in the human hippocampal formation. Intraneuronal sortilin aggregation occurred as cytoplasmic inclusions among the pyramidal neurons, co-labeled by antibodies to the extracellular domain and intracellular C-terminal of sortilin. They existed infrequently in the brains of adults, while their density as quantified in the subiculum/CA1 areas increased in the brains from elderly lacking Aβ/pTau, with pTau (i.e., primary age-related tauopathy, PART cases), and with Aβ/pTau (probably/definitive AD, pAD/AD cases) pathologies. In PART and pAD/AD cases, the intraneuronal sortilin aggregates colocalized partially with various GVD markers including casein kinase 1 delta (Ck1δ) and charged multivesicular body protein 2B (CHMP2B). Single-cell densitometry established an inverse correlation between sortilin immunoreactivity and that of Ck1δ, CHMP2B, p62, and pTau among pyramidal neurons. In pAD/AD cases, the sortilin aggregates were reduced in density as moving from the subiculum to CA subregions, wherein sorfra plaques became fewer and absent. Taken together, we consider intraneuronal sortilin aggregation an aging/stress-related change implicating protein sorting deficit, which can activate protein clearance responses including *via* enhanced phosphorylation and hydrolysis, thereby promoting GVD, sorfra, and Tau pathogenesis, and ultimately, neuronal destruction and death.

## Introduction

Senile (or neuritic) plaques (SP) and neurofibrillary tangles (NFT) are the two primary neuropathologies of Alzheimer’s disease (AD); these terms were coined in the late 1890s based on the classic histological stains (Critchley, 1929; Oĭfa, 1973; García-Marín et al., 2007; Ohry and Buda, 2015). The identification of β-amyloid peptides (Aβ) and phosphorylated Tau (pTau) as the major constituents of SP and NFT has greatly advanced the research into AD (Glenner and Wong, 1984; Masters et al., 1985). Neuropathological studies indicate that Aβ and Tau pathogenesis develop in the human brain with stereotypical spatiotemporal patterns (Serrano-Pozo et al., 2011; Veitch et al., 2019; Koychev et al., 2020). Specifically, Aβ deposition starts in the isocortex (i.e., Thal phase 1), spreads into the allocortical/limbic structures (phase 2), and further to subcortical regions (phases 3–5), while tauopathy advances through the entorhinal (Braak stages I and II), limbic (III and IV) and isocortical (V and VI) stages (Braak and Braak, 1991; Thal et al., 2002; Braak and Del Tredici, 2014). Aβ and pTau have served as neuropathological and imaging/biofluid markers for the diagnosis or risk assessment of AD, and are also being tested as mechanism-based therapeutical targets (Montine et al., 2012; Johnson et al., 2016; Buckley et al., 2017; Grothe et al., 2017; Jack et al., 2018; Schwarz et al., 2018; Long and Holtzman, 2019; Mattsson et al., 2019; Leuzy et al., 2021; Salloway et al., 2021; Beach, 2022; Chhatwal et al., 2022).

Large autopsy studies indicate that tauopathy occurs prior to microscopically detectable Aβ deposition in the human brain (Braak et al., 2011; Veitch et al., 2019; Arnsten et al., 2021; Frisoni et al., 2022). Thus, early Braak stage pTau/NFT lesions are commonly observed in the entorhinal cortex and hippocampus in the absence of cerebral Aβ deposition, a condition referred to as primary age-related tauopathy (PART) (Crary et al., 2014; Kim et al., 2019; Hickman et al., 2020; Weigand et al., 2020; Teylan et al., 2020; Humphrey et al., 2021; Walker et al., 2021). Notably, a type of cytoplasmic inclusions called granulovacuolar degeneration (GVD) also occurs early in these brain areas, and progresses with stage I involving the CA1/CA2/subiculum, stage II the CA3/CA4 and entorhinal cortex, and further stages III-V in some other cortical/subcortical regions (Woodard, 1962; Ball and Lo, 1977; Funk et al., 2011; Thal et al., 2011; Köhler, 2016). The GVD bodies occur in the so-called pretangle neurons. The causal relationship between GVD and Tau pathogenesis is a topic of growing interest (Kurdi and Alghamdi, 2020; Andrés-Benito et al., 2021; Hondius et al., 2021; Hou et al., 2020; Puladi et al., 2021).

We recently identified extracellular plaque-like deposition of *sor*tilin C-terminal *fra*gments (shorted as *sorfra*) in aged and AD human brains with Aβ and pTau pathologies (Hu et al., 2017). Sorfra plaques appear to be human-specific as they could not be detected in the brains of commonly used transgenic mouse models of AD and aged monkeys (Zhou et al., 2018). A whole-brain comparative mapping study established that sorfra plaques occurred essentially in the cerebrum, and developed spatiotemporally from the basal neocortical areas, to allocortical structures, and finally to the primary neocortical areas. This pattern of sorfra plaque formation resembles the propagation of tauopathy in the cerebrum, pointing to a certain intrinsic mechanistic link between these two lesions (Tu et al., 2020).

In our initial study, we noticed that a subpopulation of neurons around the sorfra plaques contained heavily labeled sortilin immunoreactive aggregates morphologically resembling the GVD bodies (i.e., Figures 6E1–E3 in Hu et al., 2017). During the past few years, we have collected a sufficient number of postmortem brains from youth, adult, and elderly subjects with and without AD-type neuropathology (Yan et al., 2015; Ma et al., 2019; Xu et al., 2019; Shi et al., 2020). In the current study, we carried out correlative pathological characterization and morphometric analyses using temporal lobe sections from selected samples to further understand the development of intraneuronal sortilin aggregation relative to brain aging, sorfra plaque formation, GVD and Tau pathogenesis.

## Materials and methods

### Human brain samples and tissue preparation

This study was approved by the Ethics Committee of Central South University Xiangya School of Medicine, in compliance with the Code of Ethics of the World Medical Association (Declaration of Helsinki). Postmortem human brains were banked through a willed body donation program (Yan et al., 2015), with donor’s clinical records prior to and/or during the last hospitalization obtained whenever available. All brains were histopathologically processed according to the Standard Brain Banking Protocol set by China Brain Bank Consortium (Qiu et al., 2019). Briefly, each brain was bisected, with a half preserved fresh—frozen at –70°C (for the future biochemical studies) and the other half immersed in formalin for 2–3 weeks. The fixed half brains were then cut coronally into 1-cm thick slices and blocked for the preparations of cryostat and paraffin sections as needed. Consecutive cryostat sections at 40-μm thickness were cut and stored in a cryoprotectant at –20°C. The sets of these sections were immunohistochemically processed, with the stages of Aβ, Tau, and GVD pathologies assessed for a given brain whenever applicable, according to established references (Braak and Braak, 1991; Thal et al., 2002, 2011; Braak and Del Tredici, 2014). We identified 52 brain cases out of the current repository (*n* = 180 by the end of 2021) to carry out the analyses in this study. The cases were grouped according to the age at death and the presence/extent of Aβ and pTau pathologies. Specifically, the “Adult” group consisted of cases with age below 65 years without Aβ or Tau pathologies in the brain; the “Aged” group consisted of cases above 65 years, and also lacking Aβ and Tau lesions in the brain; the “PART” group consisted of cases with tauopathy (Braak stages I–IV) but no Aβ deposition; and the pathologically probable and diagnosable AD (“pAD/AD”) group had extracellular Aβ deposition from Thal phases 1–5 and tauopathy from Braak stages I–VI in the brain (Table 1).

**TABLE 1:** Demographic information of brain donors and staging of Alzheimer-related neuropathology.

	Case #	Age (years)	Sex	Clinical diagnosis and cause of death	Postmortem delay (hours)	Braak NFT stage	Thal Aβ phase	GVD stage	Tissue usage
Adult group (*n* = 14; age 43.2 ± 13.2)	1	60	M	Heart failure	14	0	0	I	IHC
	2	55	M	Lung cancer	8	0	0	0	IHC
	3	50	F	Leukemia	8	0	0	0	IHC
	4	37	F	Leukemia	12	0	0	0	IHC
	5	31	F	Vagina cancer	10	0	0	0	IHC
	6	31	M	Heart failure	7	0	0	0	IHC
	7	28	M	Lung cancer	2	0	0	0	IHC/IF
	8	64	M	Movement disorder	18	0	0	I	IHC/IF
	9	57	M	Cerebral hemorrhage	8	0	0	0	IHC
	10	38	M	Liver cancer	8	0	0	0	IF
	11	47	M	Lung cancer	7	0	0	0	IHC/IF
	12	51	M	Lymphoma	9	0	0	0	IHC/IF
	13	22	F	Osteosarcoma	10	0	0	0	IHC
	14	37	F	Leukemia	12	0	0	0	IF
Aged group (*n* = 9; 77.9 ± 10.5)	15	79	M	Stroke	2	0	0	I	IHC
	16	68	F	Coronary heart disease	6	0	0	I	IHC
	17	67	M	Cor pulmonale	20	0	0	0	IHC
	18	62	F	Multiple myeloma	4	0	0	0	IHC/IF
	19	89	F	Multisystem failure	16	0	0	I	IHC
	20	81	M	Cardiovascular failure	6	0	0	I	IHC/IF
	21	88	F	Coronary heart disease	9	0	0	I	IHC/IF
	22	91	M	Heart failure	12	0	0	I	IHC/IF
	23	76	M	Stroke	7	0	0	I	IHC
PART group (*n* = 14; age 74.6 ± 10.6)	24	57	M	Cerebral infarction	20	I	0	I	IHC
	25	77	M	Multisystem failure	12	II	0	I	IHC
	26	65	M	Lung cancer	6	I	0	I	IHC
	27	70	M	Cholangiocarcinoma	48	I	0	I	IHC
	28	70	F	Pneumonia	12	II	0	I	IHC
	29	66	M	Cor pulmonale	10	II–III	0	I	IHC
	30	90	M	Coronary heart disease	9	III	0	III	IHC/IF
	31	95	M	Esophageal cancer	7	IV	0	II	IHC/IF
	32	66	M	Lung adenocarcinoma	10	III	0	II	IHC/IF
	33	71	M	Cerebral infarction	8	III	0	II	IHC/IF
	34	84	F	Lung cancer	10	III	0	III	IHC/IF
	35	82	M	Respiratory failure	7	IV	0	III	IHC/IF
	36	81	M	Stroke	8	III	0	II	IHC/IF
	37	71	M	Cor pulmonale	6	III	0	I	IHC/IF
pAD/AD group (*n* = 15; age 84.9 ± 8.7)	38	70	M	AD-type dementia*	8	III	4	II	IHC/IF
	39	74	F	Lung cancer (small cell)	5	IV	4	II	IHC/IF
	40	74	M	Coronary heart disease	26	IV	5	III	IHC/IF
	41	97	M	Multisystem failure*	25	IV	5	III	IHC/IF
	42	80	M	Lung cancer	15	I	3	I	IHC
	43	81	F	Chronic Renal Failure	7	IV	2	II	IHC
	44	80	F	Multiple system failure*	6	IV	5	II	IHC/IF
	45	98	M	Multiple system failure	7	IV	4	II	IHC/IF
	46	82	M	AD-type dementia*	9.5	III	5	II	IHC/IF
	47	88	M	Cor pulmonale	6	II	3	II	IHC
	48	89	M	Multiple system failure	8	III	2	III	IHC
	49	96	M	Heart disease*	20	VI	5	V	IHC/IF
	50	92	M	Coronary heart disease	12	VI	5	V	IHC/IF
	51	87	F	Multiple system failure	6	III	3	III	IHC
	52	85	M	Glioma	12	III	4	III	IHC

*Demented according to the last clinical report or enquiry with the next-of-kin of the donor. Braak neurofibrillary tangle (NFT) stages were assessed according to immunolabeling with the AT8 antibody. Thal β-amyloid (Aβ) phases were assessed according to immunolabeling with the 6E10 antibody. Staging of granulovacuolar degeneration (GVD) was based on immunolabeling with the Ck1δ antibody in reference to Thal et al. (2011).

### Immunohistochemistry and immunofluorescence

For each brain, a set of cryoprotected frozen temporal lobe sections (3–4 sections/brain, with an interval of ∼1,000 μm) passing the mid-hippocampus was immunohistochemically stained with the avidin–biotin complex (ABC) method consistently with each of the following antibodies (see Table 2 for detailed information): (1) mouse anti-Aβ 6E10, (2) mouse anti-pTau AT8, (3) goat anti-sortilin extracellular domain (ECD), (4) rabbit anti-sortilin C-terminal (CT), (5) mouse anti-casein kinase I isoform δ (CK1δ), and (6) rabbit anti-charged multivesicular body protein 2B (CHMP2B). The immunolabeling procedures were as previously described (Hu et al., 2017; Tu et al., 2020), with the labeled sections used for the confirmation of neuropathologies and assessment of the numerical densities of intraneuronal sortilin aggregates.

**TABLE 2:** Primary antibodies used in this study.

Antibody name	Manufacturer	Product code	Clone	Host species	Dilution IHC	Dilution IF
Aβ	Signet	39320	6E10	Mouse	1/4,000	1/1,000
Calnexin	Proteintech	10427-2-AP	Polyclonal	Rabbit	1/1,000	1/400
Cathepsin B	Millipore	IM27L	CA10	Mouse	1/500	1/100
Cathepsin D	Millipore	IM03	BC011	Mouse	1/1,000	1/100
CHMP2B	Abcam	ab226304	polyclonal	Rabbit	1/2,000	1/500
CK1δ	Santa Cruz	sc-55553	C-8	Mouse	1/1,000	1/200
GSK3β	Cell Signaling	12456	D5C5Z	Rabbit	1/1,000	1/200
NeuN	Millipore	MAB377	A60	Mouse	1/2,000	1/1,000
p62	Abnova	H00008878-M01	2C11	Mouse	1/1,000	1/100
p62	Bioworld	AP6006	Polyclonal	Rabbit	1/200	1/50
pS65-Ub	Millipore	ABS1513-I	Polyclonal	Rabbit	1/1,000	1/100
pTau	Invitrogen	MN1020	AT8	Mouse	1/5,000	1/2,500
pTau	Abcam	ab62639 (human Tau a.a.12–30)	Polyclonal	Sheep	1/2,500	1/1,000
Sortilin (CT)	Abcam	ab16640	Polyclonal	Rabbit	1/1,000	1/100
Sortilin (ECD)	R&D Systems	AF3154	Polyclonal	Goat	1/1,000	1/100
TOMM34	Santa Cruz	sc-101284	S-05	Mouse	1/6,400	1/2,000
TOMM34	Protein Tech	12196-1-AP	Polyclonal	Rabbit	1/6,400	1/1,000
TPPP	Santa Cruz	sc-515819	A-6	Mouse	1/3,200	1/1,000

a.a., amino acid residues; Aβ, β-amyloid; CHMP2B, charged multivesicular body protein 2B; CK1δ, casein kinase I isoform δ; CT, C-terminal; ECD, extracellular domain; GSK3β, glycogen synthase kinase-3 isoform β; IF, immunofluorescence; IHC, immunohistochemistry; NeuN, neuron-specific nuclear antigen; p62, sequestosome 1; pS65-Ub, PTEN-induced putative kinase (PINK1)-generated phospho-ubiquitin (pS65-Ub); pTau, phosphorylated Tau; TOMM34, 34 kDa-translocase of the outer mitochondrial membrane; TPPP, tubulin polymerization promoting protein. In the cases that two antibodies to the same protein are listed, the use of a particular antibody is indicated in the figure legend.

Based on the above overall assessment of immunolabeling/pathology, the individual cases (marked with “IHC/IF” in Table 1) from each subject group were selected for additional immunohistochemical and double immunofluorescent characterizations using thin-cut paraffin sections. For this purpose, a tissue block containing the hippocampal formation was dissected out from the formalin-fixed temporal lobe slice, with the samples from three to four cases embedded in the same paraffin block. Serial paraffin sections were cut at 4 μm, which were stained with individual antibodies using the ABC-peroxidase method (along with hematoxylin counterstain in some cases) (Table 2). Other paraffin sections were immunofluorescent stained with a pair of primary antibodies from two different host species. The primary antibodies used in this study were identified according to the existing references (Zhang et al., 2009; Hu et al., 2017; Andrés-Benito et al., 2021; Hondius et al., 2021; Hou et al., 2020; Puladi et al., 2021). For each antibody, pilot experiments were performed to optimize the working dilution and to check the specificity by using positive and negative assay/sample controls. For all primary antibodies, the immunohistochemical labeling was visualized with the pan-specific secondary antibody, i.e., biotinylated horse anti-mouse, rabbit, and goat IgGs (1:400; Vector Labs., Burlingame, CA, United States) followed by peroxidase-diaminobenzidine (DAB) reaction. For double immunofluorescence, the signal was visualized with an adequate combination of the Alexa Fluor^®^ 488 and Alexa Fluor^®^594 conjugated donkey anti-mouse, anti-rabbit, or anti-goat secondary antibodies (1:100, Invitrogen, Carlsbad, CA, United States), according to the species origins of the primary antibodies. The sections were counterstained with the nuclear dye Hoechst 33342 (bisbenzimide) and briefly incubated in 0.1% Sudan black to block autofluorescence before coverslipped with VECTASHIELD^®^ antifading mounting media (Vector Labs).

### Image acquisition

All immunolabeled bright-field sections were scan-imaged using the 40 × objective on a Motic–Olympus microscope equipped with an automated stage and imaging system (Motic China Group Co., Ltd., Wuhan, Hubei, China). A final autofocused, montaged and magnification-adjustable image covering the entire stained sections was digitally documented. The images were examined with the Motic viewer (Motic Digital Slide Assistant System Lite 1.0, Motic China Group Co., Ltd.), to comparatively assess the labeling across anatomical regions from low to high resolutions. Images covering the whole tissue section and subregions of interest were extracted at desired magnifications and exported for figure preparation. Quantification of the intraneuronal sortilin aggregates was also carried out using this digital interface (detailed below). All immunofluorescent sections were scan-imaged with a Keyence imaging system (KV-8000, Keyence Corporation, Osaka, Japan) using the 40 × magnification objective and a Z-stack setting of 1-μm scanning depth. The final autofocused, montaged, and magnification-adjustable digital images were used for on-screen examination and exporting of low and high magnification micrographs for figure presentation and single-cell densitometry.

### Quantitative image analysis

To estimate the incidence of intraneuronal sortilin aggregates, quantitation was carried out using randomized sampling approaches to obtain data from anatomically consistent area of interest for comparison between cases and subject groups. Counting was carried out in sections immunolabeled with the sortilin ECD antibody. The counting region was anatomically defined as a rectangular zone lateral to the DG, which covered the subicular to CA1 areas extending between the superior and inferior borders of the granule cell layer (GCL). Using the scale markers on the X and Y axes of the Motic viewer interface as distance references, this rectangular zone was divided into grids in 200 μm × 200 μm size. Subsequently, 40 grids per section were randomly generated using an Excel plug-in application, which were individually marked over the image using the X and Y coordinates of these grids (Ai et al., 2021). The numbers of neuronal somata and intraneuronal aggregates in the grids in the s.p. (representing effective sampling) were counted on-screen at 40 × magnification and recorded into MS Excel table. Specifically, the aggregates with a diameter above or 2 μm were counted, using the 20-μm scale bar as a reference as needed. This cutoff was arbitrary for a purpose of consistency, as it was practical to judge the size of an aggregate being at least one-tenth of the length of the scale on-screen. Based on the numbers obtained from two sections per brain, the percentage of neurons containing these aggregates and the average number of aggregates per neuron, were calculated for each brain case. The mean values and standard derivations (SD) of the counts were subsequently calculated for the four subject groups.

We carried out comparative single-cell densitometry to determine the trend of change in sortilin immunoreactivity inside individual neurons relative to the accumulation of GVD, p62, and pTau. The quantifications were carried out using paraffin sections from two PART cases and two pAD cases that were immunofluorescent labeled with the sortilin ECD antibody and the CK1δ, CHMP2B, p62, and pTau antibodies, respectively. High magnification (40×) images over 10 non-overlapping microscopical fields across the s.p. of the subicular to CA1 region lateral to the DG were exported from each section, with each set included the Alexa Fluor^®^ 488 and Alexa Fluor^®^594 fluorescence separately as well as a merged file. The images were converted into gray-scale tiff files, with the optical densities over individual neuronal profiles measured using the OptiQuant software. After obtaining the specific optical densities of the two antibodies from individual neurons from all the PART and pAD cases, we normalized the density data to the highest value of the same labeling (i.e., defined as 100%) from the measured neurons. The resulted relative specific optical densities of the labeling of two antibodies in the same neurons were plotted against each other, along with a correlation analysis.

To determine a spatial relevance of intraneuronal sortilin aggregation to the progression of sorfra plaque deposition, quantification was carried out in two sections per brain from the pAD/AD cases (*n* = 10) immunolabeled with the sortilin CT antibody. Four counting zones were selected in a mid-hippocampal temporal lobe section, with the first zone (zone 1) set at the area wherein the sorfra plaques became fewer and then absent, and the last zone (zone 4) approximately at the curving part of CA3. The remaining two zones (zone 2 and zone 3) were set at approximately one fourth of the distance between zone 1 and zone 4. The counting area in each zone covered radially the entire thickness and tangentially 300-μm width of the s.p.; again, we counted the aggregates with a diameter above or 2 μm, using the 20-μm scale bar as a reference in the cases that the size was difficult to judge. The numbers of aggregates per millimeter were calculated according to the sum of the aggregates and the total area of the counted grids.

### Statistical analysis and figure preparation

The numerical densities (mean ± SD) of intraneuronal sortilin aggregates among the four subject groups were statistically analyzed using one-way analysis of variance (ANOVA), with Bonferroni multiple comparison *post-hoc* tests performed to determine the existence of intergroup differences (GraphPad Prism 7.1, San Diego, CA, United States). These statistical tests were also used to compare the areal densities (mean ± S.D.) of the aggregates quantified in the four zones of the subicular to CA1–CA3 subregions in the pAD/AD cases. Pearson correlation was used for the analyses of the single-cell double immunofluorescent densitometric data to depict the relative changes of two labels among individual neurons. The minimal level of significant difference was set at *p* < 0.05. Figures were prepared with Photoshop 8.0 by assembling representative micrographs and graphs from data analyses.

## Results

### Morphological characterization and quantitative analysis of intraneuronal sortilin aggregates in human hippocampal formation

As characterized previously, sortilin immunoreactivity (IR) visualized with the antibodies to sortilin ECD and CT appeared microscopically identical in various anatomical regions in adult human brain, with the IR localized to neurons especially enriched in large-sized pyramidal or projection neurons (Hu et al., 2017; Xu et al., 2018). As observed in the temporal lobe sections from a mid-age adult case, sortilin IR in the neocortical and hippocampal pyramidal neurons appeared largely punctate at high magnifications, with fine granules (generally below 0.5 μm in diameter) seen inside the somata and proximal dendritic processes (Supplementary Figures 1A–F). These fine granules were visible in immunofluorescent labeling with these two sortilin antibodies (Supplementary Figure 1G) or sortilin and NeuN antibodies (Supplementary Figure 1H). In comparison, in sections from two elderly subjects, aggregates larger in size than the above-mentioned fine granules were seen in neocortical and hippocampal pyramidal neurons. Thus, besides the fine granular profiles, larger and more heavily labeled intracellular inclusions were found in a subpopulation of pyramidal neurons in the aged case free of cerebral Aβ/pTau pathologies, as well as in the case with cerebral pTau but no Aβ lesions (i.e., PART case) (Supplementary Figures 2A–L). These intraneuronal aggregates exhibited bright and colocalized immunolabeling by the ECD and CT sortilin antibodies (Supplementary Figures 2M,N). In sections from an AD case, the intracellular aggregates were present in many pyramidal neurons in the hippocampal formation and temporal neocortex (Supplementary Figures 3A–D). Again, they were strongly co-labeled by the sortilin ECD and CT antibodies, while the overall neuronal labeling by the CT antibody tended to reduce in the presence of extracellular sorfra deposition (Supplementary Figures 3B,B1,B2,E). It was also notable that the intracellular aggregates appeared to be reduce or were absent in neurons with heavy pTau IR (Supplementary Figure 3F).

**FIGURE 1:**
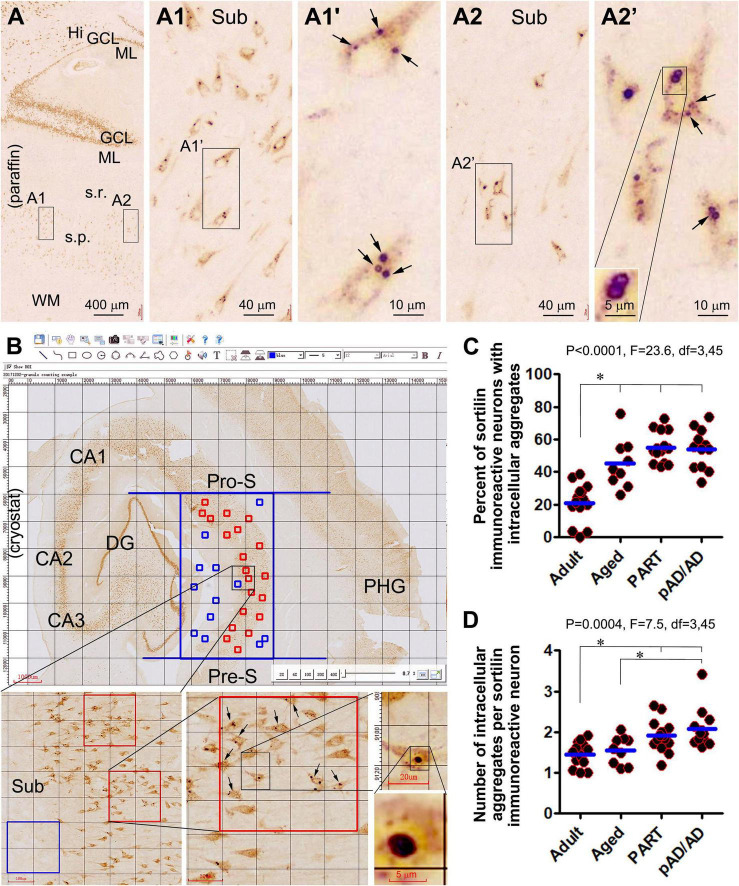
Morphology and quantification of intraneuronal sortilin aggregation in human hippocampal formation. Image panels show DAB-immunolabeling with the antibody to sortilin extracellular domain (ECD) in 4-μm thick paraffin [**(A)** and enlarged panels)] from the brain of case #33 (Table 1) and 40-μm thick cryostat [**(B)** and enlarged panels] sections from the brain of case #48. Normal-appearing sortilin immunoreactivity (IR) occurs in pyramidal neurons and granule cells in the neocortex and hippocampal formation **(A,B)**, microscopically featured by lightly to moderately labeled fine granules approximately below 0.5 μm in size **(A1–A2’**. However, heavily labeled aggregates appearing in solid and circular forms (pointed by arrows) are present inside a subpopulation of pyramidal neurons, especially prominent in the subicular and CA subregions. Quantification of intraneuronal sortilin aggregates are carried out in mid-hippocampal sections using the grid-addition option of the Motic imaging interface **(B)**. The squares in the stratum pyramidale (s.p.) are considered as effective sampling zones (marked in red), whereas those outside the s.p. are non-effective sampling zones (marked in blue). Data from individual cases of the adult, aged, primary age-related tauopathy (PART), and probable Alzheimerortiisease (pAD) and AD, groups are plotted, along with one-way ANOVA with *post hoc* tests **(C,D)**. *Difference reached statistically significance between the indicated groups. Additional abbreviations: Aβ, β-amyloid; NFT, neurofibrillary tangle; CA1–CA3, Ammon’s horn subregions; Pro-S, prosubiculum; Sub, Subiculum; Pre-S, presubiculum; PHG, parahippocampal gyrus; Hi, hilus; ML, molecular layer; s.r., stratum radiatum; WM, white matter. Scale bars are as indicated.

Examining closely in thin-cut paraffin sections, the intraneuronal sortilin aggregates exhibited a spectrum in regard to their size and labeling intensity (Figures 1A–A2’). Thus, the aggregates appeared to be derived from the normally present fine granules. As the aggregates increased in size, so did their labeling intensity. Overall, the smaller aggregates stained lighter and appeared solid (center-filled), whereas the larger ones were more heavily stained. Importantly, the largest aggregates were circular in shape at high magnification with a diameter reaching 3–5 μm (Figures 1A1,A1’,A2,A2’). In other words, the sortilin aggregates exhibited much of the same morphological characteristics of GVD as established previously (Ball and Lo, 1977; Funk et al., 2011; Thal et al., 2011; Köhler, 2016).

According to the quantification described in “Materials and methods section and illustrated in Figure 1B, the percentages of neurons containing intracellular aggregates were estimated to be 21.1 ± 11.9% (mean ± SD, same format below) in the adult group, 45.1 ± 15.3% in the aged group, 55.2 ± 9.6% in the PART group, and 54.0 ± 12.2% in the pAD/AD group. The one-way ANOVA analysis (same test below) indicated an overall difference in the frequency of neurons with the aggregates (*p* < 0.0001, *F* = 23.6, *df* = 3,45), with Bonferroni multiple comparison *post hoc* tests showing significant difference in the adult group relative to the aged, PART, and pAD/AD groups, respectively (Figure 1C). The mean number of aggregates per neuron was 1.5 ± 0.3; 1.6 ± 0.3, 1.9 ± 0.4, and 2.1 ± 0.5 in the above groups in the same listing order, respectively. Statistically, there was an overall difference in the mean values among the groups (*p* = 0.0004, *F* = 7.5, *df* = 3,45), with significant difference reached between the adult vs. PART groups, adult vs. pAD/AD groups, and aged vs. pAD/AD groups, respectively (Figure 1D).

### Verification of a differential occurrence of granulovacuolar degeneration in p62/pTau positive neurons

It is well established that GVD develops in the hippocampal formation in human brains with PART, as well as with AD-type Aβ and Tau pathologies. Specifically, GVD bodies often occur in the so-called pretangle neurons exhibiting light to moderate pTau IR (Thal et al., 2011; Köhler, 2016; Andrés-Benito et al., 2021; Hondius et al., 2021; Hou et al., 2020; Puladi et al., 2021). To replicate these findings, we prepared paraffin sections from some PART and pAD/AD cases, along with aged cases free of Aβ/pTau as control (Table 1). The GVD lesions were rarely observed in sections from the aged control cases. Therefore, immunohistochemistry and double immunofluorescence were carried out in sections from the PART and pAD/AD cases. Aβ, GVD, and pTau pathologies were examined in the hippocampal formation, with the GVD lesions validated using a panel of related antibodies (Table 2).

As seen in sections from a set of PART cases confirmed to be lack of Aβ deposition (Supplementary Figures 4A–D), a small group of pyramidal neurons in the subicular subregions, CA1 and CA2 exhibited light-to-moderate pTau IR (Supplementary Figures 5A–E’), with a few of them heavily labeled (Supplementary Figures 5C’–E’). Sortilin IR in the subicular and hippocampal pyramidal neurons occurred generally as fine intracellular granular profiles, with heavily labeled aggregation bodies seen in a subpopulation of neurons (Supplementary Figures 6A–E’). Moreover, CK1δ and CHMP2B IR were observed in many pyramidal neurons in CA2, and a few in CA1 and subicular subregions including the prosubiculum (Pro-S), subiculum (Sub), presubiculum (Pre-S), and parasubiculum (Para-S) (Supplementary Figures 7, 8). The labeling appeared as intracellular bodies among individual neurons, although a diffuse cytoplasmic IR occurred in the somata and proximal dendrites in the neurons with densely packed GVD bodies (Supplementary Figures 7A1,B1,A2,B2, 8A1,B1,A2,B2). The distribution and morphology of pS65-Ub (Supplementary Figure 9) and TPPP (Supplementary Figure 10) labeled neurons appeared to be comparable to that labeled with the CK1δ and CHMP2B antibodies, including the presence of diffuse cytoplasmic IR in the neurons with a large number of GVD bodies (Supplementary Figures 9B1,D1, 10B,C). In another PART case (Supplementary Figures 11, 12), a larger number of pTau immunoreactive neurons and processes was present in the hippocampal formation. Many of the pTau positive neurons appeared to contain tangles, giving their distorted morphological configuration, uneven or thread-like distribution of IR in the somata. Some pTau positive neurons showed a loss of nucleus and/or cytoplasmic constituents as seen in hematoxylin counterstain preparation (Supplementary Figure 12). In a neighboring section, p62 immunolabeled neuronal and neuritic profiles were present in the hippocampal subregions, and exhibited similar morphological appearance as with the pTau positive neurons (Supplementary Figure 13). In other adjacent sections, many neuronal profiles were also immunolabeled for the GVD markers, including CK1δ (Supplementary Figure 14), CHMP2B (Supplementary Figure 15), TOMM34 (Supplementary Figures 16, 17), TPPP (Supplementary Figure 18), and GSK3β (Supplementary Figure 19). Again, besides the heavily labeled inclusion bodies, diffuse cytoplasmic IR was visualized by these markers in some neurons (Supplementary Figures 14D, 15C, 16D, 17B–D, 19D).

Further, in the section from a case in the pAD/AD group, abundant Aβ IR occurred in the temporal neocortex, subicular subregions and hippocampal formation (Supplementary Figure 20A). Also, Aβ IR appeared as compact and diffuse plaques, and as subpial and vascular amyloidosis (Supplementary Figures 20B–D). A great amount of pTau IR was present in the above anatomical regions (Supplementary Figure 21A). In fact, most pTau positive pyramidal neurons exhibited tangles at high magnification (Supplementary Figures 21B,C). In other adjacent sections immunolabeled with the CK1δ (Supplementary Figure 22) and CHMP2B (Supplementary Figure 23) antibodies (labeling of other GVD markers not shown), a large number of pyramidal neurons contained GVD bodies. Again, diffuse cytoplastic CK1δ/CHMP2B IR was seen in the neurons with densely packed GVD bodies. However, cytoplasmatic IR was also found in morphologically distorted neuronal profiles wherein no or few GVD bodies could be observed (Supplementary Figures 22B’, 2BC’).

**FIGURE 2:**
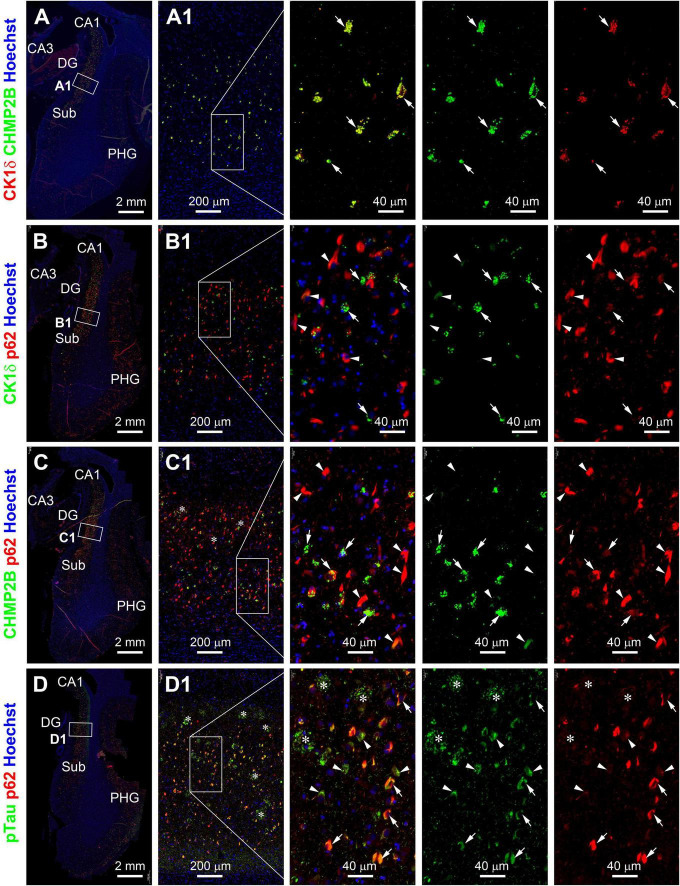
Representative images showing double immunofluorescent characterization of granulovacuolar degeneration (GVD) relative to tauopathy in temporal lobe paraffin sections from a case (#39) in the pAD/AD group. Casein kinase 1 delta (Ck1δ) and charged multivesicular body protein (CHMP2B) labeling are extensively colocalized among GVD bodies (arrows) in hippocampal pyramidale neurons [**(A,A1)** and enlarged panels]. Ck1δ positive GVD bodies exist preferably in pretangle neurons (arrows) but not those with tangle-like structures (arrowheads) expressing p62 (rabbit antibody indicated in Table 2 and **B,B1** and enlarged panels). A similar differential colocalization pattern is seen among the labeled neurons for CHMP2B and p62 (mouse antibody in Table 2 and **C,C1** and enlarged panels). p62 (mouse antibody) and pTau (sheep antibody) labeling colocalize in the somata of both pretangle neurons (arrows) and neurons with tangles (arrowheads). pTau, but not p62, IR is localized to neuritic clusters (*) [**(D,D1)** and enlarged panels]. Abbreviations are as defined in Figure 1. Scale bars are as indicated.

We further carried out double immunofluorescence using the paraffin sections from the PART and pAD/AD cases to examine the colocalization pattern of GVD, p62, and pTau IR in pyramidal neurons. CK1δ and CHMP2B IR were colocalized extensively at the GVD bodies among individual pyramidal neurons in the PART (Supplementary Figure 24A) and AD cases (Figure 2A). In general, CK1δ or CHMP2B labeled GVD bodies occurred in neurons with light or moderate p62 or pTau IR. In fact, CK1δ or CHMP2B labeled GVD bodies were fewer or not present in neurons with heavy p62 or pTau IR (Supplementary Figures 24B,C and Figures 2B,C). Also, p62 and pTau IR were extensively colocalized in the somata and dendrites. Neuronal somata with relatively light p62/pTau IR exhibited a granular appearance in many cases (pointed by arrows), whereas those with heavy p62/pTau IR often appeared to contain tangle-like structures (pointed by arrowheads) (Figures 2D,D1; Supplementary Figures 24B1, C1). It should be noted that neuritic clusters (at the putative sites of neuritic plaques according to their arrangement) exhibited bright pTau IR. In contrast, little or no p62 IR occurred in these neuritic clusters (Figure 2D1 and enlargements, marked with asterisks).

### Partial colocalization of granulovacuolar degeneration markers in intraneuronal sortilin aggregates

Having characterized the relationship of GVD to p62/pTau pathology, we set to determine the extent to which some previously reported GVD markers would colocalize at the intraneuronal sortilin aggregates. Double immunofluorescence for sortilin and one of the GVD markers, respectively, was carried out using additional paraffin sections from representative PART and pAD/AD cases. Overall, a differential colocalization pattern was observed in sortilin/CK1δ (Supplementary Figure 24D), sortilin/CHMP2B (Supplementary Figure 24E), sortilin/TOMM34 (Supplementary Figure 25), and sortilin/GSK3b (Supplementary Figure 26) double immunofluorescence. Thus, a subset of intraneuronal sortilin aggregates colocalized with CK1δ (Supplementary Figure 24D1, pointed by arrows; also see Figure 5), CHMP2B (Supplementary Figure 24E1, pointed by arrows; also see Supplementary Figure 30), TOMM34 (Supplementary Figure 25), or GSK3b (Supplementary Figure 26) immunolabeling. However, in the same microscopic field, there also existed CK1δ, CHMP2B, TOMM34, and GSK3β immunolabeled GVD bodies that did not show an increased sortilin IR (Supplementary Figures 24D,E, 25, 26).

**FIGURE 3:**
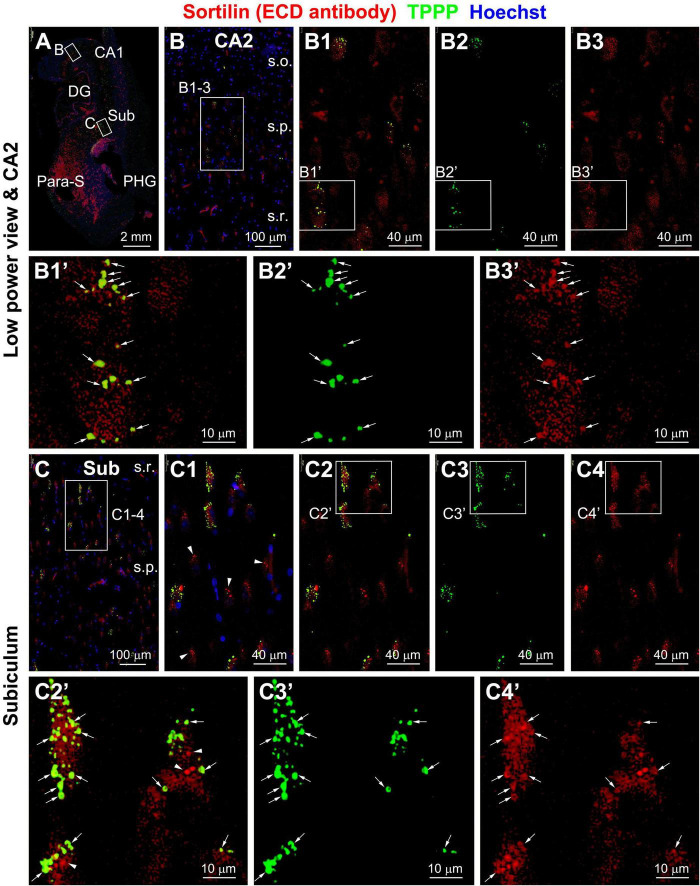
Representative images showing colocalization of tubulin polymerization promoting protein (TPPP) among intraneuronal sortilin aggregates in hippocampal and subicular pyramidal neurons (section from case #34). Boxed areas in **(A)** are enlarged sequentially [**(B,B1–B3,B1’–B3’)**; **(C,C1–C4,C1’–C4’)**]. In a subpopulation of pyramidal neurons, TPPP positive granulovacuolar degeneration (GVD) bodies appear to be mostly labeled for sortilin (pointed by arrows) **(B1’–B3’,C1’–C3’)**. However, TPPP labeling is absent in other pyramidal neurons containing sortilin immunoreactive GVD bodies [**(C1,C2’)**, pointed by arrowheads for example]. Abbreviations are as defined in Figure 1. Scale bars are as indicated.

**FIGURE 4:**
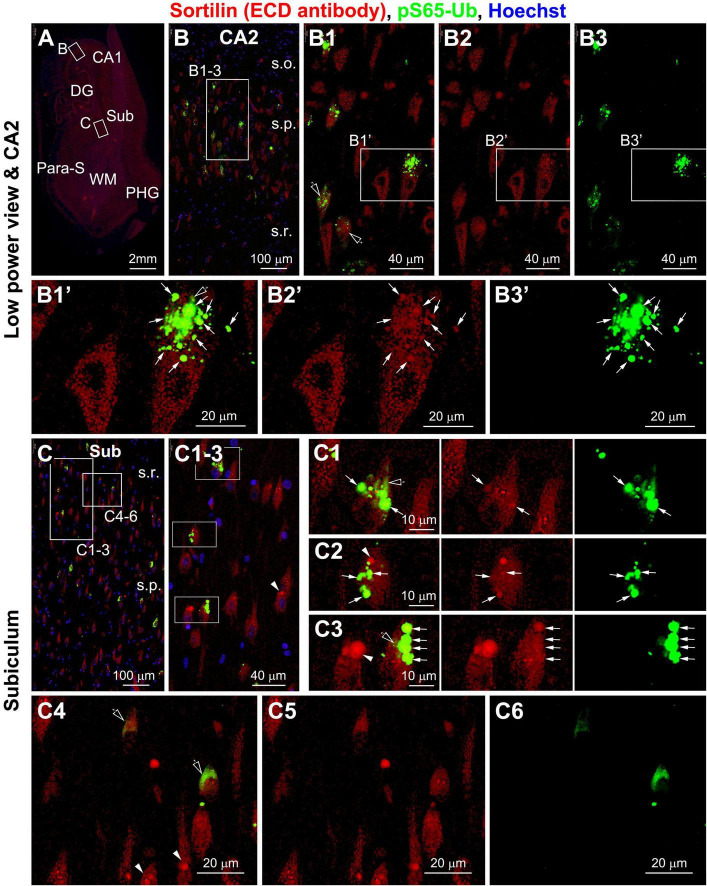
Representative images showing colocalization of PTEN-induced putative kinase 1 (PINK1)-generated phospho-ubiquitin (pS65-Ub) with intraneuronal sortilin aggregates in hippocampal and subicular pyramidal neurons (section from case #34). Boxed areas in **(A)** are enlarged sequentially [**(B,B1–B3,B1’–B3’)**; **(C,C1–C6)**]. The pS65-Ub positive granulovacuolar degeneration (GVD) occur in a subpopulation of pyramidal neurons, which also show relatively strong sortilin labeling (as pointed by arrows) [**(B1,B1’–B3’,C1–C3)**]. Again, some sortilin-labeled aggregates do not exhibit pS65-Ub reactivity (pointed by arrowheads) [**(C1–C3,C4–C6)**]. Spreading cytosol pS65-Ub labeling is seen between the GVD bodies (pointed by open arrows) [**(B1–B3,B1’–B3’,C1–C3)**], but may fill up the neuronal somata in some neurons with rare or no large-sized sortilin immunoreactive aggregates [**(C4–C6)**]. Abbreviations are as defined in Figure 1. Scale bars are as indicated.

In sortilin/TPPP double immunofluorescence there existed a great extent of colocalization among the intracellular granular profiles (Figures 3A–C4’). This colocalization was mostly evident in pyramidal neurons packed with a large number of the TPPP positive GVD bodies in granular and circular forms (Figures 3B1’–B3’,C2’–C4’). However, there were also neurons that contained sortilin immunoreactive aggregates that did not exhibit TPPP IR (Figures 3B1–B3’,C1–C4’). In sortilin/pS65-Ub double immunofluorescence (Figures 4A–C), there was also a great extent of colocalization of the two markers in neurons at the GVD bodies (Figures 4B,C).

**FIGURE 5:**
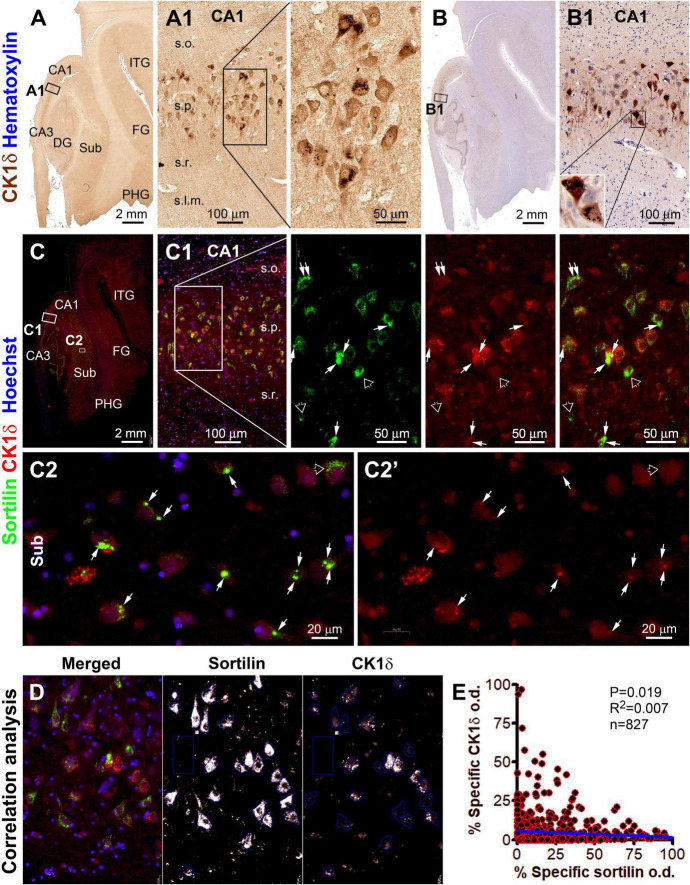
Microscopical characterization and single-cell densitometric analysis of casein kinase 1 delta (CK1δ) relative to sortilin immunolabeling in hippocampal/subicular pyramidal neurons. **(A,A1,B,B1)** Show DAB-immunolabeling in temporal lobe sections with and without hematoxylin counterstain (from case #44). At high magnification, a large subset of pyramidal neurons in CA1 contain intraneuronal granular bodies in varying numbers. Light-to-moderate cytosol reactivity is seen in the somata and dendritic processes of some neurons [**(A1,B1)**]. **(C)** and enlarged panels **(C1,C2,C2’)** show sortilin (ECD antibody) and CK1δ double immunofluorescence with Hoechst 33342 nuclear counterstain in an adjacent paraffin section. CK1δ labeling occurs in a subset of sortilin-labeled neurons, with colocalized immunofluorescence occurred at relatively large-sized aggregates (pointed by arrows). However, CK1δ labeling is not present in other sortilin-labeled neurons with normal-looking fine granules or with large-sized aggregates (pointed by open arrows). **(D)** Illustrates the methodology for correlated single-cell densitometry detailed in “Materials and methods” section. **(E)** Plots the normalized (percentages) specific optical density (o.d.) values of CK1δ against sortilin labeling in 827 CA1 and subicular pyramidal neurons measured in identically processed paraffin sections from 2 PART and 2 pAD cases. Pearson analysis indicates a statistically (*p* = 0.019) significant inverse correlation with a relatively small coefficient (*R*^2^ = 0.007). FG, fusiform gyrus; ITG, inferior temporal gyrus. Other abbreviations are as defined in Figure 1. Scale bars are as indicated.

It should be noted that, as with the labeling pattern seen in peroxidase-DAB preparation, the GVD antibodies also displayed relatively light and spreading cytoplasmic immunofluorescence in some pyramidal neurons (as seen for CK1δ in Supplementary Figures 24A–D and Figures 5A–D; CHMP2B in Supplementary Figures 24D1,E1, and pS65-Ub in Figures 4B1’–B3’,C1–C3, pointed by open arrows). Such cloudy cytoplasmic immunofluorescence was often present between densely packed GVD bodies, but could also occur in proximity to isolated GVD bodies in some neurons. In addition, the diffuse cytoplasmic immunofluorescence could occur in a large part of or the entire intraneuronal space of a neuron with distorted morphology (Figures 4C4–C6 and Supplementary Figures 26B1–B3,C1–C4).

We carried out additional double immunofluorescent characterizations to explore sortilin colocalization with some cell organelle markers (Supplementary Figures 27–29). Sortilin IR showed some degree of colocalization with the IR of both the endosomal marker calnexin and the Golgi body marker GM130. However, there were also some areas within the cell where the IR for calnexin or GM130 did not colocalize with sortilin, and *vice versa* (Supplementary Figures 27A–A2). It should be noted that calnexin IR also occurred in relatively small-sized cellular profiles likely interneurons, which were poorly labeled by the sortilin antibody (Supplementary Figures 27A1,A2, pointed by arrows). Also, GM130 IR appeared granule-like in neuronal somata, which was partially colocalized with sortilin IR by the presence of distinct yellow areas in merged images (Supplementary Figures 27B,C and enlarged panels). Again, the relatively small-sized cellular profiles were found to exhibit GM130 IR but little sortilin IR. In addition, sortilin IR was partially colocalized in hippocampal and subicular pyramidal neurons with immunolabeling for two lysosomal markers, cathepsin B (Supplementary Figure 28) and cathepsin D (Supplementary Figure 29).

### Alteration of neuronal sortilin IR relative to granulovacuolar degeneration, p62 and pTau accumulation

The colocalization pattern and relative change between sortilin and CK1δ IR among individual neurons are shown in Figure 5. As seen in the immunohistochemically stained paraffin section from a pAD case, CK1δ-labeled GVD bodies were distinctly present in a subgroup of pyramidal neurons in the CA and subicular subregions (Figures 5A–B1). In double immunofluorescence, many CK1δ positive GVD bodies expressed bright sortilin IR (Supplementary Figures 5C,D, pointed by arrows). The overall sortilin IR appeared to be more or less reduced in the pyramidal neurons with GVD bodies compared to those without (Figures 5C1,C2,C2’). The single-cell densitometric data indicated a statistically significant inverse correlation between the densities of sortilin and CK1δ IR among individual pyramidal neurons (*p* = 0.019; *R*^2^ = 0.007; *n* = 827 neurons) (Figure 5E). Similarly, the overall sortilin IR tended to reduce in the neurons that contained a large number of CHMP2B positive GVD bodies in double immunofluorescence (Supplementary Figures 30A–E). The densitometric analysis indicated an inverse correlation between sortilin and CHMP2B IR among CA1 and subicular pyramidal neurons (*p* = 0.001; *R*^2^ = 0.018; *n* = 615 neurons) (Supplementary Figure 30F).

**FIGURE 6:**
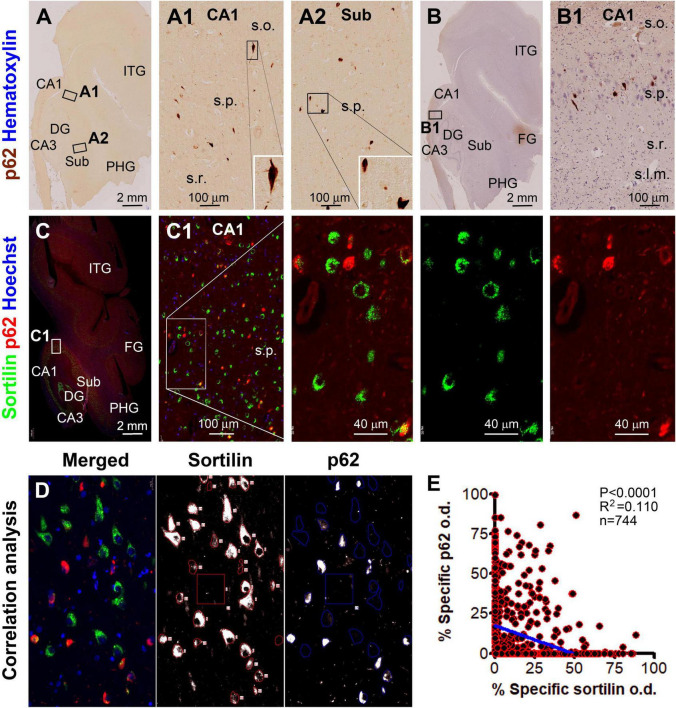
Microscopical characterization and single-cell densitometry of p62 relative to sortilin immunolabeling in hippocampal/subicular pyramidal neurons. **(A,A1,B,B1)** Are low power and enlarged views of DAB-immunolabeling with the mouse p62 antibody in a paraffin section from case #44. Note that some p62 labeled neurons appear in morphology that is essentially comparable to pTau positive neurons with tangles (inserts). **(C)** and enlarged views show p62 and sortilin double immunofluorescence with Hoechst nuclear counterstain. There is a full spectrum of variability for p62/sortilin colocalization across the labeled neuronal population in a single microscopic field. Among the neurons exhibiting colocalization, sortilin labeling tends to reduce as p62 immunofluorescence increases [**(C1)** and enlarged views]. **(D)** Illustrates the methodology for correlative single-cell densitometry. **(E)** Plots the normalized specific o.d. values obtained from 744 neurons from 2 PART and 2 pAD cases. There exists a significant inverse correlation between p62 and sortilin reactivity among individual neurons (*p* < 0.0001, *R*^2^ = 0.11, Pearson analysis). FG, fusiform gyrus; ITG, inferior temporal gyrus. Other abbreviations are as noted in Figure 5. Scale bars are as indicated.

**FIGURE 7:**
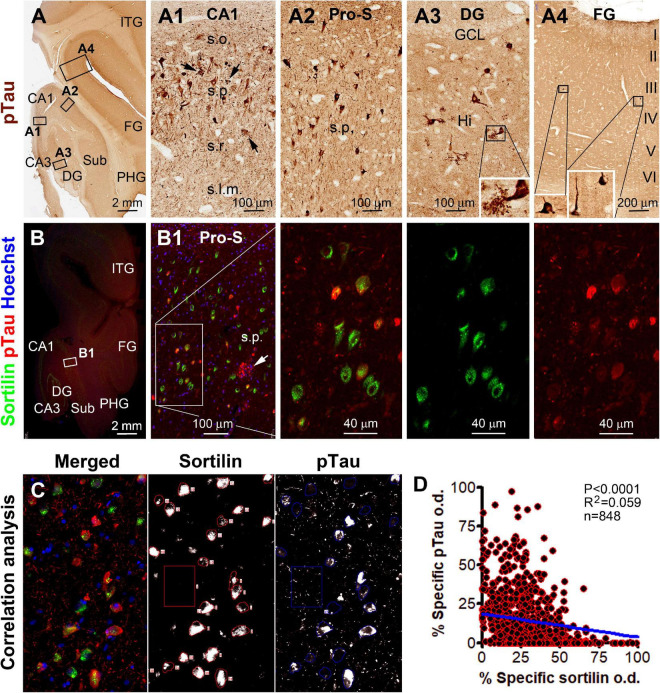
Microscopical characterization and single-cell densitometry of phosphorylated Tau (pTau) relative to sortilin immunolabeling in hippocampal/subicular pyramidal neurons. **(A–A4)** Show low-power view and enlarged views of pTau (AT8 antibody) labeling in different hippocampal and neocortical areas as indicated (section from case #44). **(A3)** And insert show pTau labeled mossy cells in the hilus. Neuritic clusters are seen in the CA1 and subicular areas (pointed by arrows). In double immunofluorescence [**(B,B1)** and enlarged panels, and **(C)**], there exists a full spectrum of variability regarding the extent of colocalization of pTau and sortilin. Thus, neurons exhibiting only sortilin and pTau immunolabeling, respectively, represent the two ends of this spectrum. **(C)** Shows the appearance of labeled neurons seen in the OptiQuant interface for correlative single-cell densitometry. There exists a distinct inverse correlation between pTau and sortilin reactivity among individual neurons (*p* < 0.0001, *R*^2^ = 0.059, Pearson analysis). Abbreviations are as noted in Figure 5 **(D)**. Scale bars are as indicated.

In the hippocampal section from the above-mentioned pAD case, a large subpopulation of pyramidal neurons in all CA sectors and subicular areas were immunohistochemically labeled for p62 and pTau to various intensities, including many darkly labeled neuronal profiles that appeared to be filled with tangles (Figures 6A–B1, 7A–A4). In double immunofluorescence, by comparatively examining the labeled neurons in the same microscopic field, sortilin IR was reduced as p62 IR or pTau IR emerged and increased in intensity in the neuronal somata. In fact, sortilin IR was greatly or completely lost in the neuronal profiles densely packed with p62/pTau immunofluorescent product (Figures 6C,C1,D, 7B,B1,C). Single-cell densitometric analyses confirmed a distinct inverse correlation between sortilin IR and p62 IR (*p* < 0.0001; *R*^2^ = 0.110; *n* = 744 neurons; Figure 6E), and between sortilin IR and pTau IR (*p* < 0.0001; *R*^2^ = 0.059; *n* = 848 neurons; Figure 7D), among the individual hippocampal pyramidal neurons.

### Regional relationship of intraneuronal sortilin aggregation with sorfra plaque formation

**FIGURE 8:**
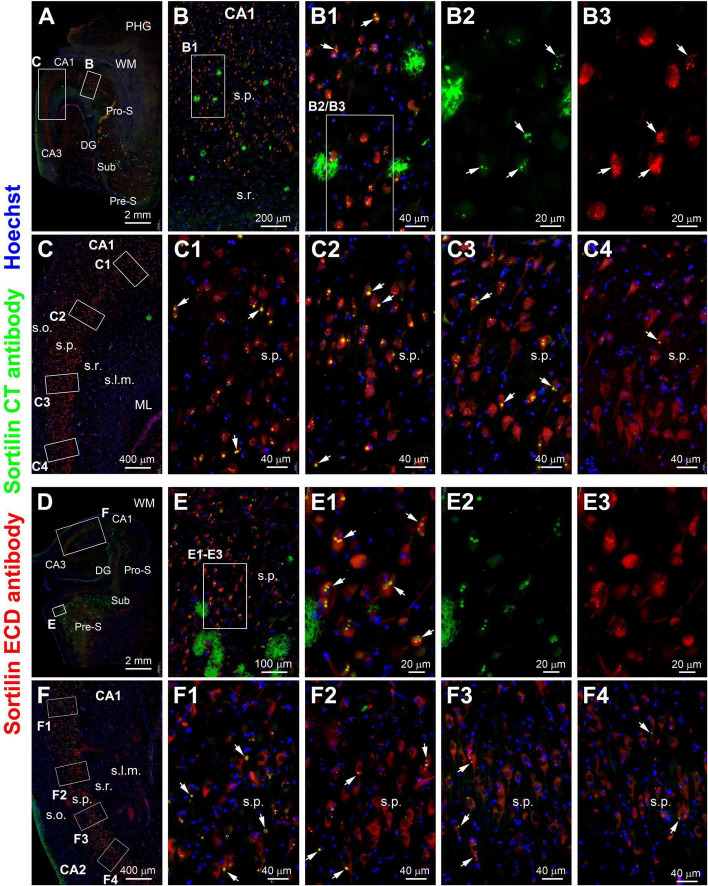
Double immunofluorescence illustrating a regional relationship between intraneuronal sortilin aggregation and sorfra plague formation in the hippocampal formation. **(A)** and enlarged views [**(B–C4)**] are from case #39 and [**(D–F4)**] are from case #40. In both cases, extracellular sorfra plaques displayed by the CT antibody are abundantly present in the temporal neocortex and subicular cortex, with their amounts reduced as moving from the subiculum (Sub) to prosubiculum (Pro-S), and further to CA1, such that no sorfra plaques are seen in the stratum pyramidale (s.p.) of CA2 and CA3 **(A,D)**. Intraneuronal sortilin aggregation bodies (examples are pointed by arrows) co-labeled by the two antibodies are frequently observed among the pyramidal neurons in the hippocampal or subicular areas wherein sorfra plaques are present [**(B,B1–B3,E,E1–E3)**]. However, the number (or frequency) of pyramidal neurons containing intraneuronal sortilin aggregates are progressively reduced as moving from the distal CA1 to CA3 areas [**(C,C1–C4,F,F1–F4)**]. Abbreviations are as defined in Figure 1. Scale bars are as indicated.

**FIGURE 9:**
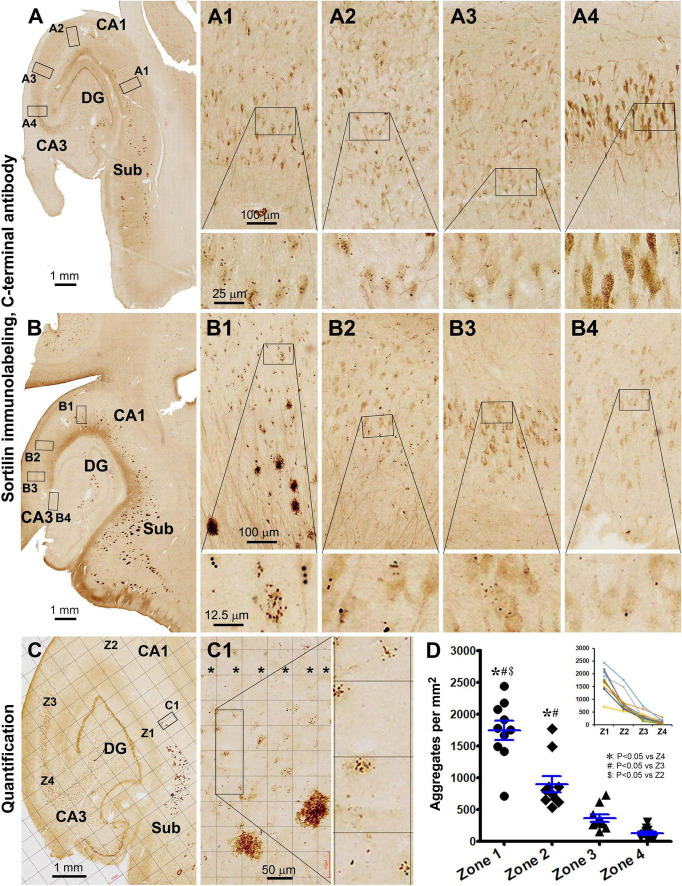
Quantitative analysis of the regional density of intraneuronal sortilin aggregates using temporal lobe sections immunolabeled with the CT sortilin antibody. [**(A–A4)** Are from case #49; **(B–B4)** from case #39, and **(C–C1)** from case #51]. A trend of regional expansion of sorfra plaques in the stratum pyramidale (s.p.) from the subicular to CA1, CA2, and CA3 subregions is noticeable by comparing the images from the three cases [**(A–C)**]. Note that the densities of intraneuronal aggregates are also reduced as moving from the subicular to CA3 direction [**(A1–A4,B1–B4)**]. **(C)** And enlarged views show a zone-based quantification detailed in “Materials and methods,” with areal densities of the aggregates in four zones (Z1–Z4, in **C**) across s.p. (* in width of 300 μm) are quantified on-screen at high resolution using the Motic viewer. **(D)** Summarizes the mean densities in the four zones obtained from 10 pAD/AD cases, with the inserted graph plotting the tendency lines in individual cases. Statistical testing results (one-way ANOVA) are marked, with *, # and $ symbols indicating the existence of difference. Abbreviations are as defined in Figure 1. Scale bars are as indicated.

A pattern of regional progression of extracellular sorfra deposition could be recognized by comparing the distribution of the plaques in temporal lobe sections from pAD/AD cases exhibiting sorfra pathology at stages 2–3 and Aβ pathology at Thal phases 2 and above (Tu et al., 2020). Thus, based on the immunolabeling of the sortilin CT antibody, sorfra plaques were found to expand progressively from the subicular areas to CA1, CA2, and CA3, and the hilus as the overall amount of sorfra deposition increased in the temporal lobe regions (Figures 8, 9A–C). A trend of decrease in the intracellular sortilin aggregation bodies was observed as moving from subicular regions to the farther sectors of the CA subareas (Figures 8A–9F4, 9A–A4,B–B4).

Thus, to determine a spatial relevance of intraneuronal sortilin aggregation to the progression of sorfra plaque deposition, a zoned-based quantification was carried out in sections immunolabeled with the sortilin CT antibody (Figure 9C). The areal densities (mean ± SD) of intraneuronal sortilin aggregates were 1,739 ± 479, 896 ± 404, 361 ± 182, and 126 ± 85 aggregates per mm^2^ in zones 1, 2, 3, and 4, respectively. The one-way ANOVA analysis indicated that there existed an overall difference between the mean values among the four zones (*p* < 0.0001, *F* = 47.3; *df* = 3, 36). Bonferroni’s multiple comparison test indicated significant intergroup difference between all paired zones except for zone 3 in comparison with zone 4 (Figure 9D).

## Discussion

**FIGURE 10:**
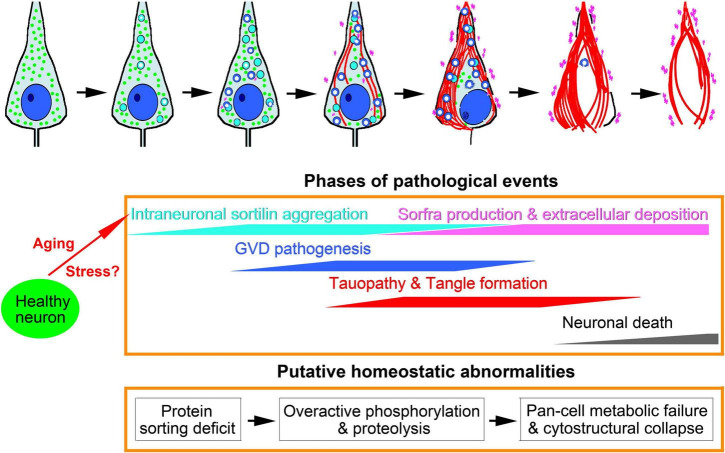
Schematic representation of intraneuronal sortilin aggregation in relevance to granulovacuolar degeneration (GVD), Tau pathogenesis, neuronal death and sorfra plaque formation. Major pathologies are symbolized in the top cell drawings, which are interpreted with colored texts and propensity trends in the middle frame. The underlying pathophysiological and pathological implications are hypothesized in the low frame. We propose that aging and stress are the primary factors promoting intraneuronal sortilin aggregation, which implicates early onset protein sorting deficit. At a certain point, aberrant protein modification (including proteolysis and phosphorylation), and cleavage and degradation machineries, are activated, marking the formation of neurochemically distinct GVD bodies. The rupture of vacuolar bodies releases enzymatically active GVD contents that further catalyze broader destructive effects to cell membrane, organelles and cytoskeleton, resulting in membrane leakage, autophagy, mitophagy, necrophage, microtubule breakdown, and formation of tangles. Along with these degenerative cascades, normal neuronal continents for metabolic and structural maintenance are lost. Thus, neuronal damage and death in partnership with the advance of tauopathy is a consequence of multisystem metabolic crisis and cytostructural collapse. We propose that sorfra products are derived from sortilin cleavage(s) by some GVD-associated enzymes, which are released *via* vesicular secretion and following cytostructural destruction, leading to microscopically detectable accumulation in the extracellular space.

Extracellular sorfra deposition is a recently identified AD-related proteopathy (Hu et al., 2017). It is important to first understand this new lesion relative to Aβ and Tau as the AD hallmark pathologies. We have reported that, while manifested microscopically as a typical plaque lesion, extracellular sorfra deposition does not always co-occur with Aβ deposition, and it develops in the human cerebrum similar to the spatiotemporal pattern of tauopathy (Hu et al., 2017; Zhou et al., 2018; Tu et al., 2020). This study extends to an intraneuronal sortilin pathology in human hippocampal formation, which is morphologically and neurochemically related to the GVD bodies. To reconcile our findings with the advance in the understanding of GVD in brain aging and AD, we schematically proposed a chain of related pathogenic events including GVD development, pTau accumulation, sorfra formation, and neuronal death (Figure 10).

### Intraneuronal sortilin aggregation can progress into granulovacuolar degeneration

Granulovacuolar degeneration was first identified in the hippocampus of demented subjects, microscopically featured as argentophilic and hematoxyphilic granular and vacuolar inclusions inside the pyramidal neurons (Simchowicz, 1911). Electron microscopic studies established that these lesions are membrane-rich cytoplasmic inclusions, with the granular and vacuolar forms implicating an early to-late pathologic evolution (Hirano et al., 1968; Okamoto et al., 1991). Other studies documented the occurrence of GVD in association frequently with AD, but also with brain aging and other neurodegenerative diseases (Woodard, 1962; Tomlinson and Kitchener, 1972; Ball and Lo, 1977; Xu et al., 1992). Since the late 1990s, a growing number of molecular markers were found to immunolabel the GVD bodies (McShea et al., 1997; Ghoshal et al., 1999; Stadelmann et al., 1999; Ishizawa et al., 2003; Lagalwar et al., 2007; Hoozemans et al., 2009; Kadokura et al., 2009; Yamazaki et al., 2010; Castellani et al., 2011; Nishikawa et al., 2014). A recent laser microdissection-mass spectrometry study reported the elevation of more than 100 proteins in GVD-containing pyramidal neurons in AD human brain (Hondius et al., 2021). The GVD markers or constituents identified so far appear to be largely implicative of proteostasis impairment, including abnormal protein folding, aggregation and clearance, and aberrant biochemical modification such as overactive phosphorylation, hydrolysis, and glycolysis. In pathological perspectives, the previous immunohistochemical studies on GVD bodies have been related to lysophagy (Funk et al., 2011; Wiersma et al., 2019; Yamoah et al., 2020), mitophagy (Hou et al., 2020), and necrophagy (Koper et al., 2020; Van Schoor et al., 2021), besides Tau hyperphosphorylation and formation of tangles (Ikegami et al., 1996; Ghoshal et al., 1999; Andrés-Benito et al., 2021). However, the origin and pathogenic course of GVD remain elusive, with the causal/effective relationship of this lesion to other neurodegenerative pathologies or processes being a subject of discussion.

In this study, we found small- and large-sized intraneuronal sortilin aggregates in granular and circular forms morphologically characteristic of the GVD bodies. These aggregates were observed in a subset of hippocampal/subicular pyramidal neurons in the adult, aged, PART, and pAD/AD cases, with an overall trend of increase in their frequency. Thus, they already developed in adult and aged individuals without cerebral Aβ and Tau pathologies. In PART and pAD/AD cases, they existed more broadly than the GVD bodies labeled by previously reported GVD antibodies in the pyramidal neurons in a given brain. Double immunofluorescence showed that a subset of these sortilin aggregation bodies co-expressed some existing GVD markers (CK1δ, CHMP2B, TPPP, pS65-Ub, and p62). Notably, these latter markers labeled the GVD bodies “*de nova*” (even with one microscopically evident granule in a cell). On the other hand, sortilin-labeled GVD bodies appeared to “derive” from normally present fine granular labeling (of intraneural membranous organelles).

Granulovacuolar degeneration is considered an aging-related neuronal pathology. Aging is a critical contributor to proteostasis impairment (Stein et al., 2022). In the human brain, sortilin is enriched in large-sized principal/projection neurons that would need a high load of protein sorting for metabolic and cytostructural maintenance in the somata and processes (Xu et al., 2019). Sortilin IR appeared to be associated with neuronal surface, endoplasmic reticulum, Golgi apparatus, and lysosomes (rather than restricted to a particular type of the above cellular components). This neuronal sortilin expression pattern is consistent with a role of this protein in support of diverse membrane signal transduction, and transportation, secretion and degradation of various partner proteins in neurons (Petersen et al., 1997; Wähe et al., 2010; Xu et al., 2018; Malik and Willnow, 2020). Accordingly, the occurrence of sortilin aggregates inside neurons would be indicative of the existence of abnormal protein sorting, trafficking, and accumulation. It should be noted that our current data could not determine whether the sortilin aggregation occurs initially at a particular part of the intracellular membranous systems, e.g., secretory granules, endosome, trans-Golgi network, and lysosome (Xu et al., 2018; Malik and Willnow, 2020), or could be related to all of these compartments. It is also an open question whether sortilin itself is functionally impaired at first, thereby playing an initiative role in the development of GVD.

Granulovacuolar degeneration formation is also considered a response to stress (Castellani et al., 2011; Thal et al., 2011; Asadi et al., 2021). The impact of stress on neurodegeneration in the human brain has been under intensive investigations (Ionescu-Tucker and Cotman, 2021), with data pointing to early onset and most dramatic alterations in bioenergetics metabolites in the hippocampus (Song et al., 2021). Indeed, the subicular/hippocampal areas serve as a neurocircuitry “hub” integrating the cerebral neocortex and limbic system (Berron et al., 2020). This region would likely function with a high burden of neuronal activity requiring dynamic molecular orchestration inside the somata and processes. The pyramidal neurons here may be therefore inherently vulnerable to metabolic stress, and hence, maladaptive failure in proteostasis maintenance, which may underscore the development of GVD, PART, and dendritic dystrophy (Shi et al., 2020).

### Granulovacuolar degeneration bodies may serve as a catalyzer underlying multifaceted proteostatic disturbances

The causal relationship between GVD and Tau pathogenesis is a matter of debate (Wiersma et al., 2020; Andrés-Benito et al., 2021; Puladi et al., 2021). Cell culture and transgenic mouse brain studies suggest that pTau seeds can induce GVD formation (Ishizawa et al., 2003; Köhler et al., 2014; Wiersma et al., 2019). However, several observations do not appear well coherent with that tauopathy drives GVD in the human brain (Andrés-Benito et al., 2021; Hondius et al., 2021). Granulovacuolar degeneration bodies occur in pretangle neurons, but are reduced and lost in tangle-filled neurons. Some GVD markers are kinases for the phosphorylation of Tau but also other proteins, while others do not have a clearly defined interplay with pTau. Moreover, there are much more types of phosphorylated proteins than pTau in GVD associated neurons (Lagalwar et al., 2007; Kadokura et al., 2009; Rudrabhatla and Pant, 2011; Thal et al., 2011; Dammer et al., 2015; Tagawa et al., 2015; Yan et al., 2018; Yamaguchi et al., 2019; Drummond et al., 2020; Koper et al., 2020; Sathe et al., 2020).

We observed intraneuronal sortilin aggregates in the brains of mid-age and even some young adult humans without microscopically detectable tauopathy. In double immunofluorescence, intraneuronal sortilin aggregates could colocalize with CK1δ and GSK3β that serve as phosphorylation kinases (Ghoshal et al., 1999; Schwab et al., 2000; Leroy et al., 2002; Ferrer et al., 2021). These sortilin aggregates could co-express CHMP2B and p62 that are involved in endolysosomal dysfunction and autophagy (Kaivorinne et al., 2010; Midani-Kurçak et al., 2019). They could also colocalize with pS65-Ub and TOMM34 that are related to mitochondrial protein trafficking and mitophagy (Faou and Hoogenraad, 2012; Hou et al., 2020; Hondius et al., 2021). Further, the aggregates could colocalize with TPPP and pTau (in pretangle neurons), relating to microtubule stability and disassembly (Kovács et al., 2004; Oláh et al., 2011; Hondius et al., 2021). Therefore, the occurrence of various GVD markers may represent a critical stage of intraneuronal sortilin aggregation whereby multiple pathogenic protein processing cascades are activated in neurons in a non-compensable manner (Figure 10).

### Tau-associated neuronal death may relate to multisystem molecular/cellular collapse

A progression from early pTau accumulation to ultimately neuronal death is conceivable based on the morphological evolution as observed comparatively from pretangle neurons to tangle-containing neurons, and to ghost-like cell remains (Braak et al., 2006). As discussed above, the various GVD-associated molecular markers are linked to multiple potentially harmful cascades. These latter may lead to excessive autophagy, mitophagy, and necrophagy, as well as microtubule disability and cytoskeleton collapse, which may jointly result in cell self-destruction and finally neuronal death.

The above notion is supported by our microscopical and single-cell densitometric analysis of sortilin relative to other pathological labels. Notably, many GVD markers exhibited a spreading cytosol reactivity in both DAB immunolabeling and immunofluorescence (Andrés-Benito et al., 2021; Hondius et al., 2021). This diffuse cytoplasmic labeling often occurred near the large-sized vacuolar bodies and could fill up the neurons with densely packed GVD bodies. Thus, the pathogenesis of GVD appears to proceed to a “mature” stage such that the vacuolar bodies may rupture, thereby releasing the enzymatically active contents to catalyze even broader destructive cascades to cell membrane, organelles and cytoskeleton. Moreover, our single-cell densitometry revealed an inverse relationship of sortilin IR relative to that of CK1δ, CHMP2B, p62, and pTau among hippocampal/subicular pyramidal neurons. The coefficients (*R*^2^) of sortilin/p62 IR and sortilin/pTau IR were greater than that of sortilin/CK1δ and sortilin/CHMP2B. Double immunofluorescence depicted a full spectrum of colocalization variability between sortilin vs. p62 and sortilin vs. pTau among pyramidal neurons. The reduced expression of sortilin including those at the intracellular aggregates indicates a loss of normal cellular components along with p62/pTau accumulation. In fact, the loss of intraneuronal sortilin may lead to a “burnout” effect such that the GVD bodies could no longer be formed, therefore also reduced and lost in neurons at a certain point of tangle accumulation. Taken together, Tau-associated neuronal death would be likely a consequence of multisystem molecular and cellular collapse (Figure 10).

### Sorfra plaque formation may result from proteolytic and cytostructural deficits

The extracellular sorfra deposits are expected to be sortilin CT fragments derived from proteolytic processing of the full-length protein (Hu et al., 2017). The chemical identity of sorfra and the corresponding “sortilin-cleaving enzyme(s)” are yet to be determined *via* comprehensive biochemical study and the setup of proper *in vitro* systems. We attempted to gain more pathogenic insight into sorfra formation in the human brain to facilitate the future translational research.

The data obtained through this study point to a likelihood that intraneuronal sortilin aggregation is a precursor of sorfra formation and deposition. The aggregates were heavily labeled by both antibodies against sortilin ECD and CT. The former recognizes only the full-length protein ∼100 kDa, while the latter additionally detects fragments ∼40, ∼25, and ∼15 kDa in western blot (Hu et al., 2017). The CT antibody appears to have a high affinity to sorfra, because in AD brain sections its labeling is preferentially localized to the plaques than the neuronal profiles that are still detectable by the ECD antibody (Hu et al., 2017). Thus, the intraneuronal aggregates should contain concentrated full-length sortilin as well as its CT fragments. The quantitative data obtained from the four subject groups showed the occurrence of sortilin aggregates in some adult, aged and PART cases before the detection of extracellular sorfra plaques (i.e., in the pAD.AD cases). Our zone-based quantification indicated a progressive decrease in the density of the sortilin aggregates from the direction of the subicular toward CA3 subregions, in parallel with the presence to absence of the extracellular sorfra plaques. In other words, the occurrence of sortilin aggregation bodies regionally occur ahead of the development of sorfra plaque formation. Based on the above findings, we propose that sorfra may be produced through sortilin cleavage(s) by some GVD-associated proteolytic enzymes. This speculation might explain as to why sortilin IR is not invariably increased among all GVD bodies as visualized by other markers. In this regard, it is noteworthy that there are two putative phosphorylation sites on the sortilin CT. Therefore, GVD-associated phosphorylation kinases might also participate in sorfra production. Sorfra might be released by vesicular secretion in pretangle neurons (Yamoah et al., 2020) and *via* cell membrane leakage along with Tau-associated cytostructural destruction and neuronal death, consequently accumulate, and deposit in the extracellular space (Figure 10).

## Conclusion

In summary, this study has identified intraneuronal sortilin aggregation in the pyramidal neurons of human hippocampal formation from adults to elderly subjects with cerebral Tau and Aβ pathologies. A subset of the sortilin-enriched aggregates shows morphological and neurochemical characteristics of the GVD bodies, although sortilin reactivity appears to be not elevated at some GVD bodies visualized by other markers. Altered intraneuronal sortilin expression is also found to be relevant to intraneuronal accumulation of pTau and sorfra formation. As an overview, our findings are in line with emerging evidence that proteostasis impairment may play a critical role in pathogenic cascades leading to Alzheimer-related neuropathology and neurodegeneration.

## Data availability statement

The raw data supporting the conclusions of this article will be made available by the authors, without undue reservation.

## Author contributions

X-XY: conceptualization. JJ, CY, J-QA, Q-LZ, X-LC, TT, LW, and Z-YL: methodology. JJ, CY, TT, LW, and X-SW: formal analysis and investigation. X-XY: writing the original draft. X-XY, CC, W-PG, and JM: reviewing and editing. X-XY, AP, ET, and X-PW: funding acquisition. CC: resources. All authors read and approved the final manuscript.
