# Supplementary material for: Intraneuronal sortilin aggregation relative to granulovacuolar degeneration, tau pathogenesis and sorfra plaque formation in human hippocampal formation
Source: Front Aging Neurosci. 2022 Aug 1;14:926904. doi: 10.3389/fnagi.2022.926904 (PMC9376392; doi:10.3389/fnagi.2022.926904)
Supplement: Supplementary file 1 [file Data_Sheet_1.PDF]

Supplemental Figures 1-30

Intraneuronal Sortilin Aggregation Relative to Granulovacuolar Degeneration, Tau Pathogenesis and Sorfra Plaque Formation in Human Hippocampal Formation

Juan Jiang[1], Chen Yang[1], Jia-Qi Ai[1], Qi-Lei Zhang[1], Xiao-Lu Cai[1], Tian Tu[2], Lily Wan[1], Xiao-Sheng Wang[1], Hui Wang[1], Aihua Pan[1], Jim Manavis[3], Wei-Ping Gai[1], Chong Che[4], Ewen Tu[5], Xiao-Ping Wang[6], Zhen-Yan Li[7]*, Xiao-Xin Yan[1]*

[1]Department of Anatomy and Neurobiology, Central South University Xiangya School of Medicine, Changsha, Hunan 410013, China
[2]Department of Neurology, Xiangya Hospital, Changsha, Hunan 410008, China
[3]Faculty of Health and Medical Sciences, The University of Adelaide, Adelaide, SA 5005, Australia
[4]GeneScience Pharmaceuticals Co., Ltd., Changchun High-Tech Dev. Zone, Changchun, Jilin 130012, China
[5]Department of Neurology, Brain Hospital of Hunan Province, Changsha, Hunan 410007, China
[6]Department of Psychiatry, The Second Xiangya Hospital, Changsha, Hunan 410031, China
[7]Department of Neurosurgery, Xiangya Hospital, Changsha, Hunan 410008, China

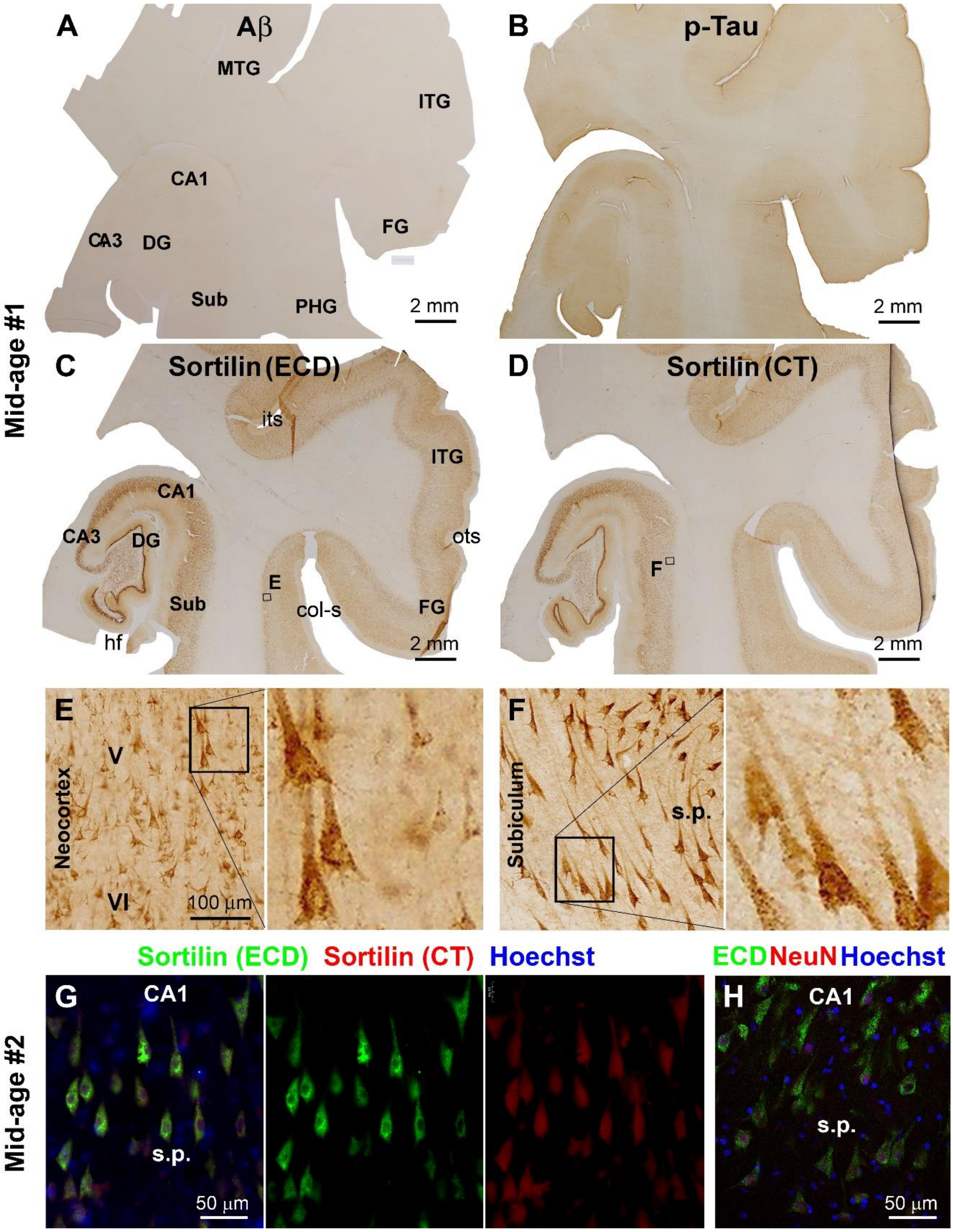

Supplemental Figure 1. Normal immunolabeling pattern of sortilin in temporal lobe areas in the brains from mid-age human subjects (cases #2 and #3 in Table 1). (A) and (B): lack of β-amyloid (Aβ) and pTau labeling in the sections. (C): Immunolabeling with the antibody to sortilin extracellular domain (ECD). (D): Immunolabeling with the antibody to sortilin C-terminal (CT). (E, F): High power views of neocortical and subicular neurons. (G): Colocalization of the ECD and CT antibody labeling. (H): Colocalization of sortilin with neuron-specific nuclear antigen (NeuN). Abbreviations: CA1, CA2, CA3: Ammon's horn subareas; DG: dentate gyrus; Sub: subiculum; FG: fusiform gyrus; PHG: parahippocampal gyrus; ITG: inferior temporal gyrus; MTG: middle temporal gyrus; col-s: collateral sulcus; ots: occipitotemporal sulcus; s.p.: stratum pyramidale.

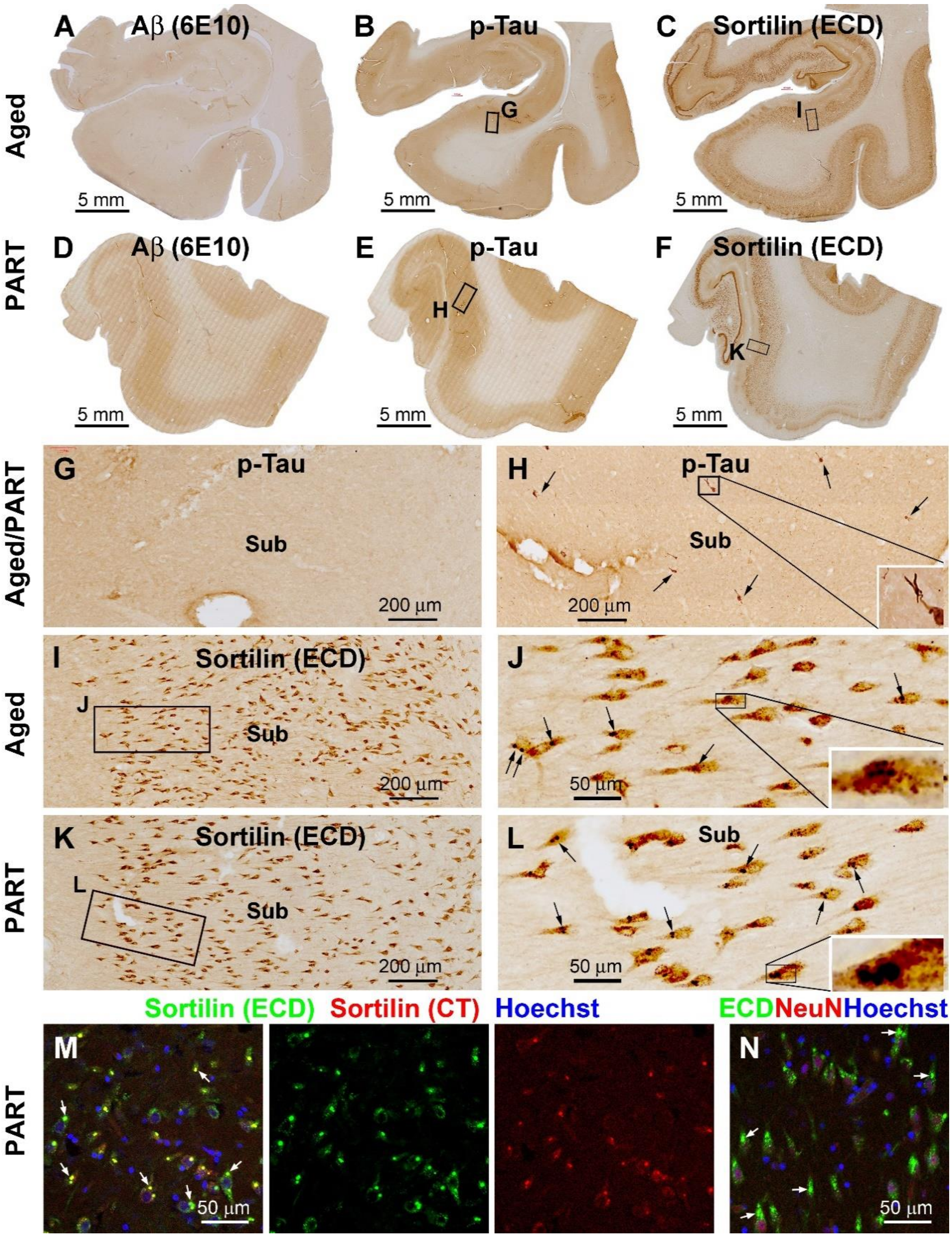

Supplemental Figure 2. Images of immunolabeled temporal lobe sections from an aged case (#16) and a case with primary age-related tauopathy (PART) (#25). (A) and (D) show the lack of Aβ deposition in the sections from both cases. (B, E, G and H): Lack (G at high magnification) and presence (H) of pTau-labeled neurons (pointed by arrows) in the aged and PART cases, respectively. (I)-(L): Intraneuronal sortilin aggregates (pointed by arrows) are present in the subicular pyramidal neurons in both cases. (M): Colocalized immunofluorescent labeling by the sortilin ECD and CT antibodies at the intracellular aggregation bodies. (N): Intracellular sortilin aggregates in neurons double-labeled for the neuron-specific nuclear antigen (NeuN).

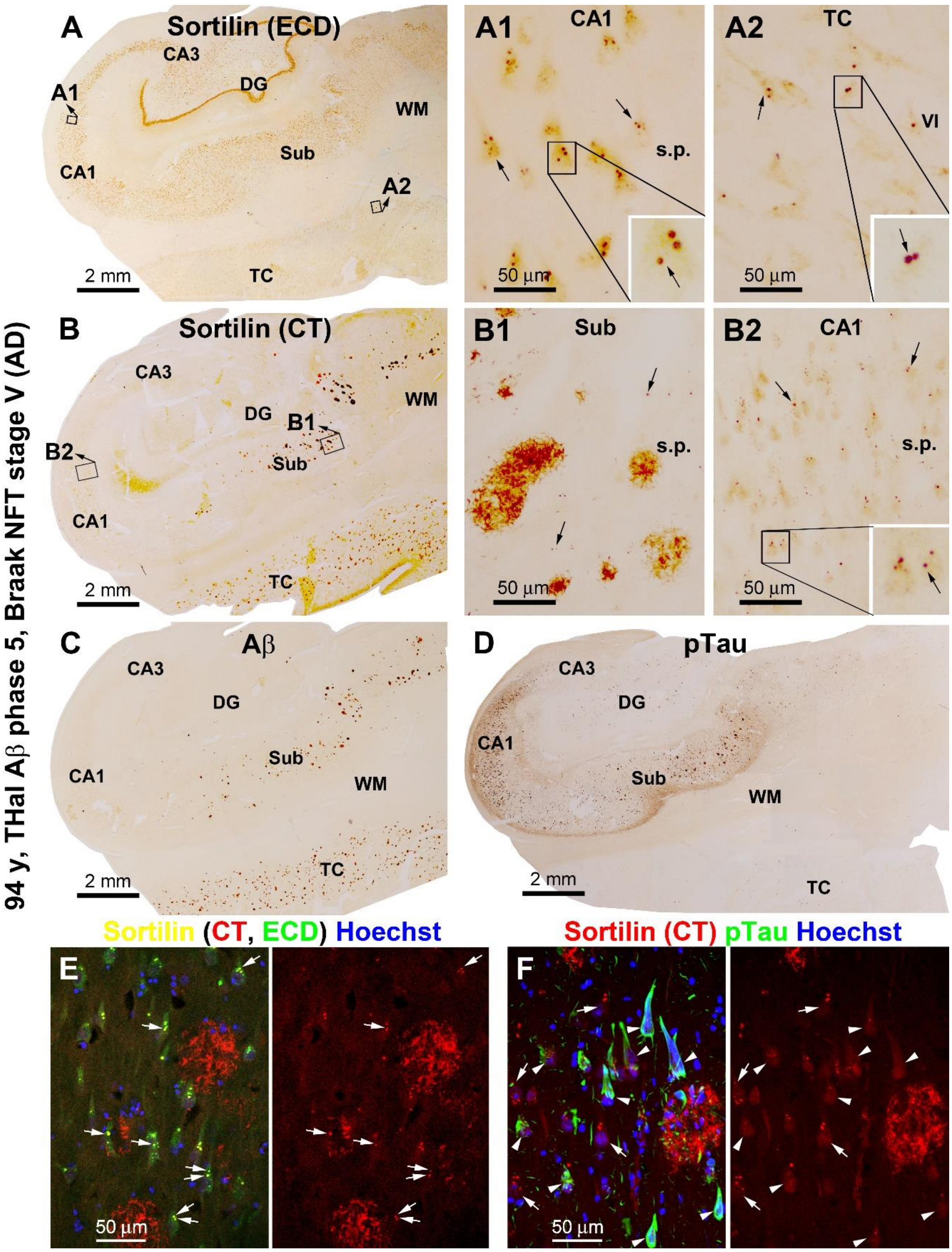

Supplemental Figure 3. Images of immunolabeled temporal lobe sections from subjects with Alzheimer's disease (AD) (cases used in Hu et al., 2017). (A-A2) and (B-B2) are low power and enlarged views of sortilin labeling by the two antibodies; note the intraneuronal aggregates as pointed by arrows. (C) and (D): Aβ and pTau pathologies in the temporal neocortex and hippocampal formation in this case. (E): Colocalized ECD and CT antibody labeling at the intracellular aggregation bodies (pointed by arrows). (F) Intracellular sortilin aggregates can occur in pTau negative as well as positive neurons (pointed by arrowheads), but are occasionally seen or absent in pTau-labeled neurons heavily packed with tangle-like structures (pointed by arrowheads).

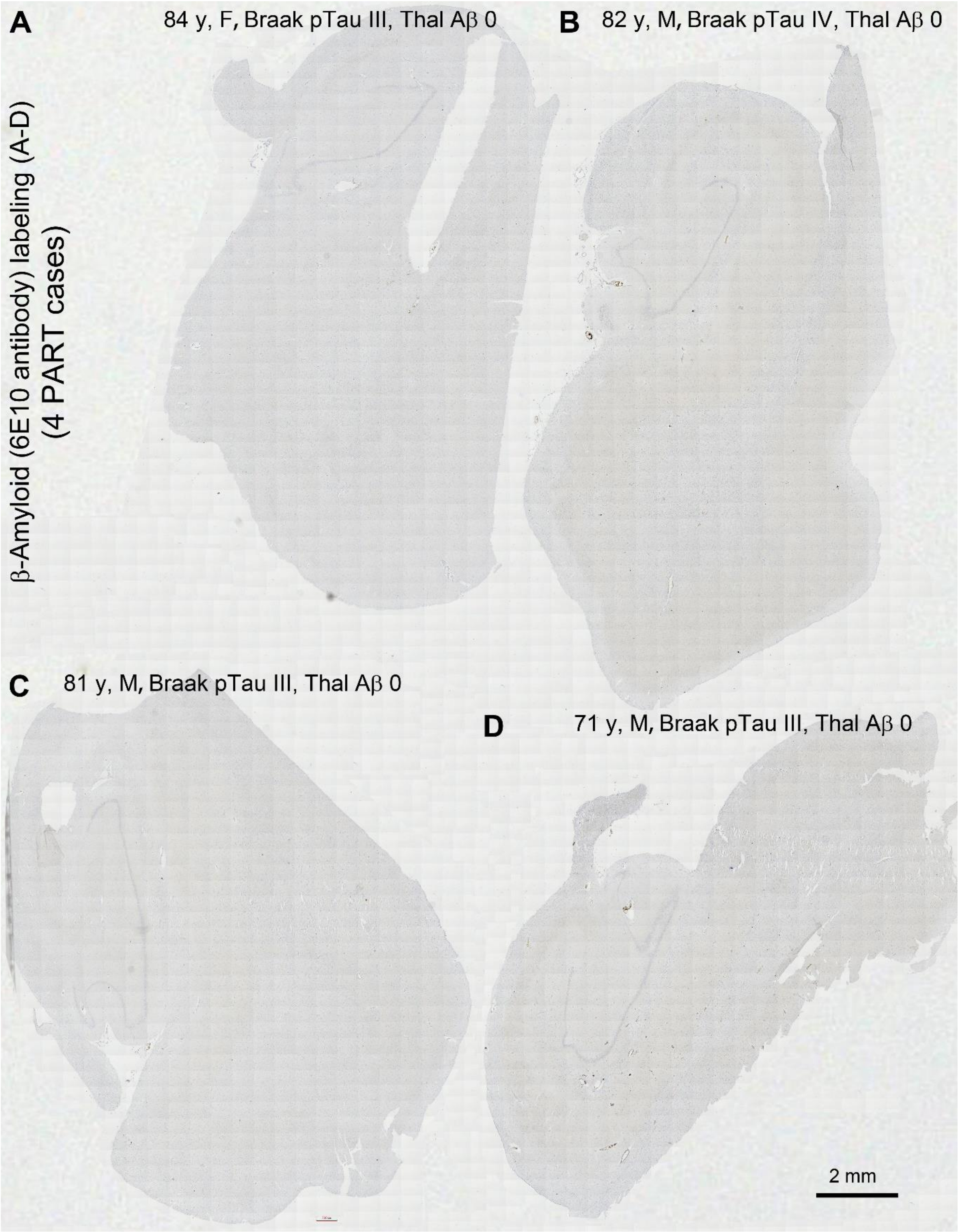

Supplemental Figure 4. Images showing the lack of β-amyloid (Aβ) deposition in four cases (#33, 34, 35, 36) with primary age-related tauopathy (PART) in the brain. Tissue blocks from the 4 cases were embedded in the same paraffin block, with the sections histologically processed together. The Braak staging of tauopathy and Thal staging of Aβ pathology of the brains are as indicated. Adjacent sections from this block were immunolabeled for other antibody markers, with representative images shown in Supplemental Figures 5-10.

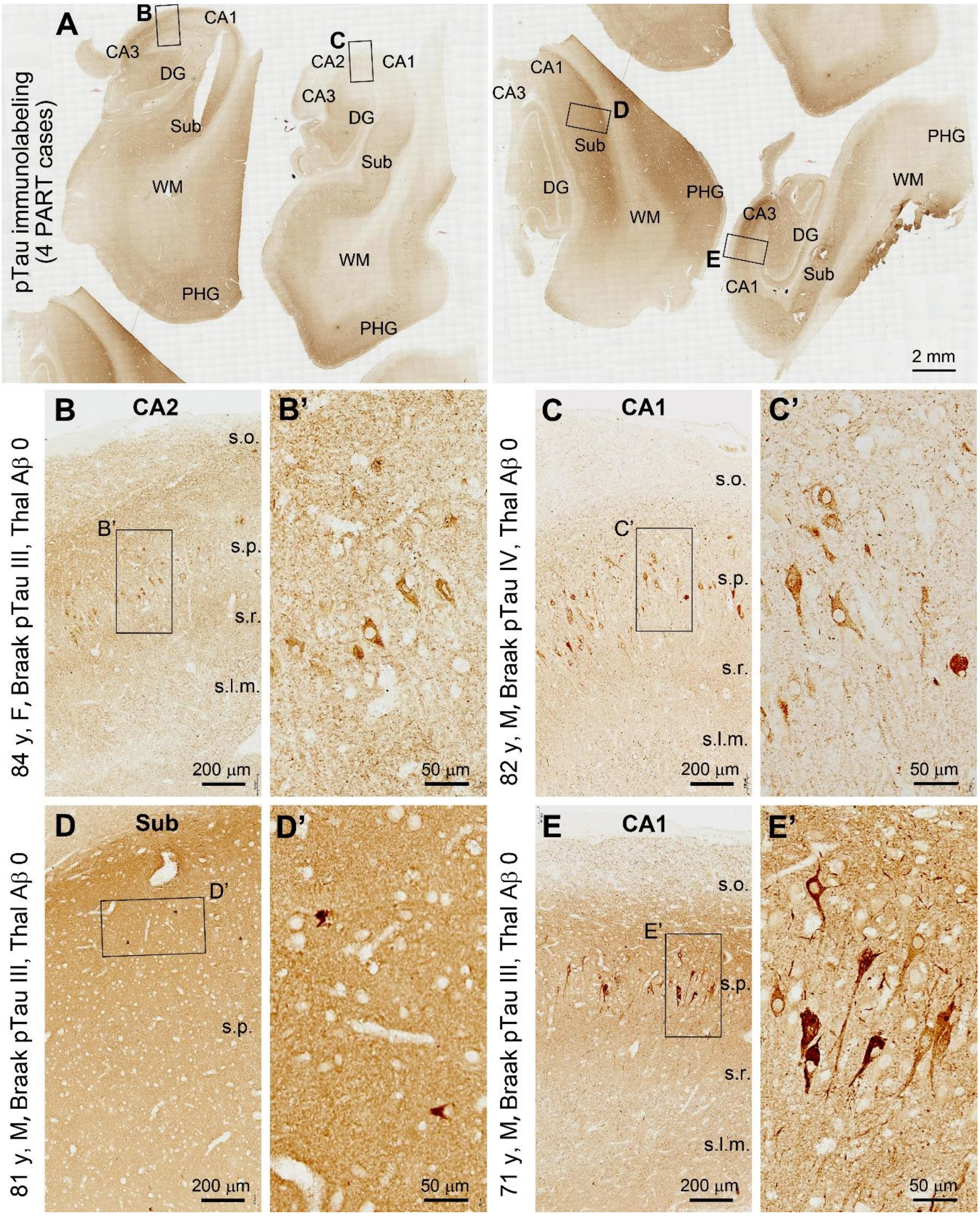

Supplemental Figure 5. Images showing pTau immunolabeling in paraffin sections from the four PART cases that are included in Supplemental Figure 4. (A) shows low magnification views, with framed areas enlarged as panels (B-E'). Most pTau positive neurons are lightly and moderately labeled, whereas some neurons had dense immunolabeling in the somata and dendritic processes. In the two latter cases, some neurons are heavily labeled with pTau, but appear to contain tangles (D, D', E, E'). Abbreviations: CA1, CA2, CA3: Ammon's horn subareas; DG: dentate gyrus; Sub: subiculum; PHG: parahippocampal gyrus; s.o.: stratum oriens; s.p.: stratum pyramidale; s.r.: stratum radiatum; s.l.m.: stratum lacunosum-moleculare; WM: white matter.

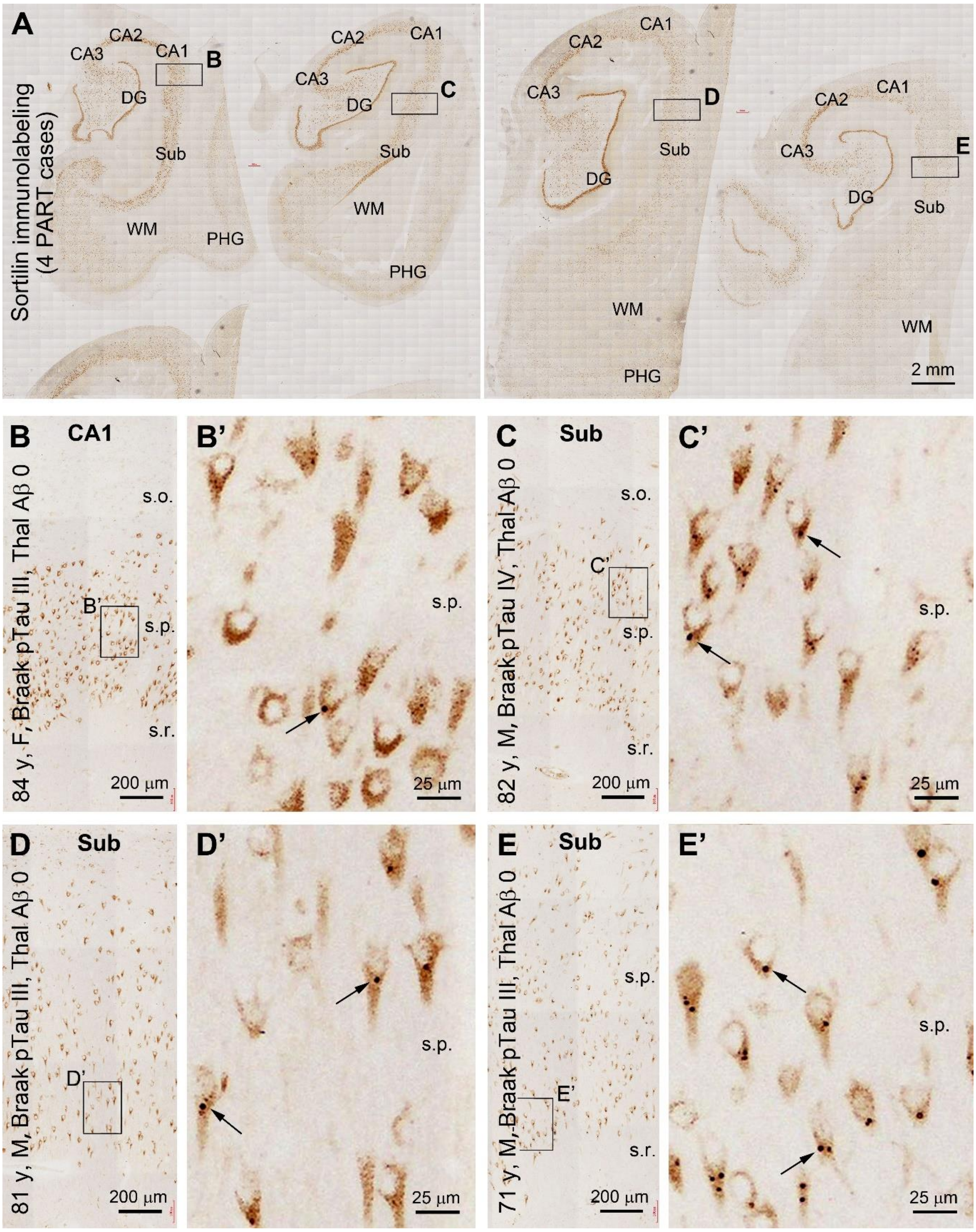

Supplemental Figure 6. Images showing sortilin immunolabeling with the extracellular domain antibody in paraffin sections from the PART cases included in Supplemental Figure 4. (A) shows the low magnification views, with framed areas enlarged as panels (B-E'). Sortilin IR appears largely as fine granules inside the somata and proximal dendritic processes in the subicular and hippocampal pyramidal neurons. The enlarged high magnification panels (B', C', D' and E') show intraneuronal aggregation bodies with varying sizes and exhibiting enhanced sortilin immunoreactivity in some pyramidal neurons in the subicular and CA1 subregions (examples are pointed by arrows).

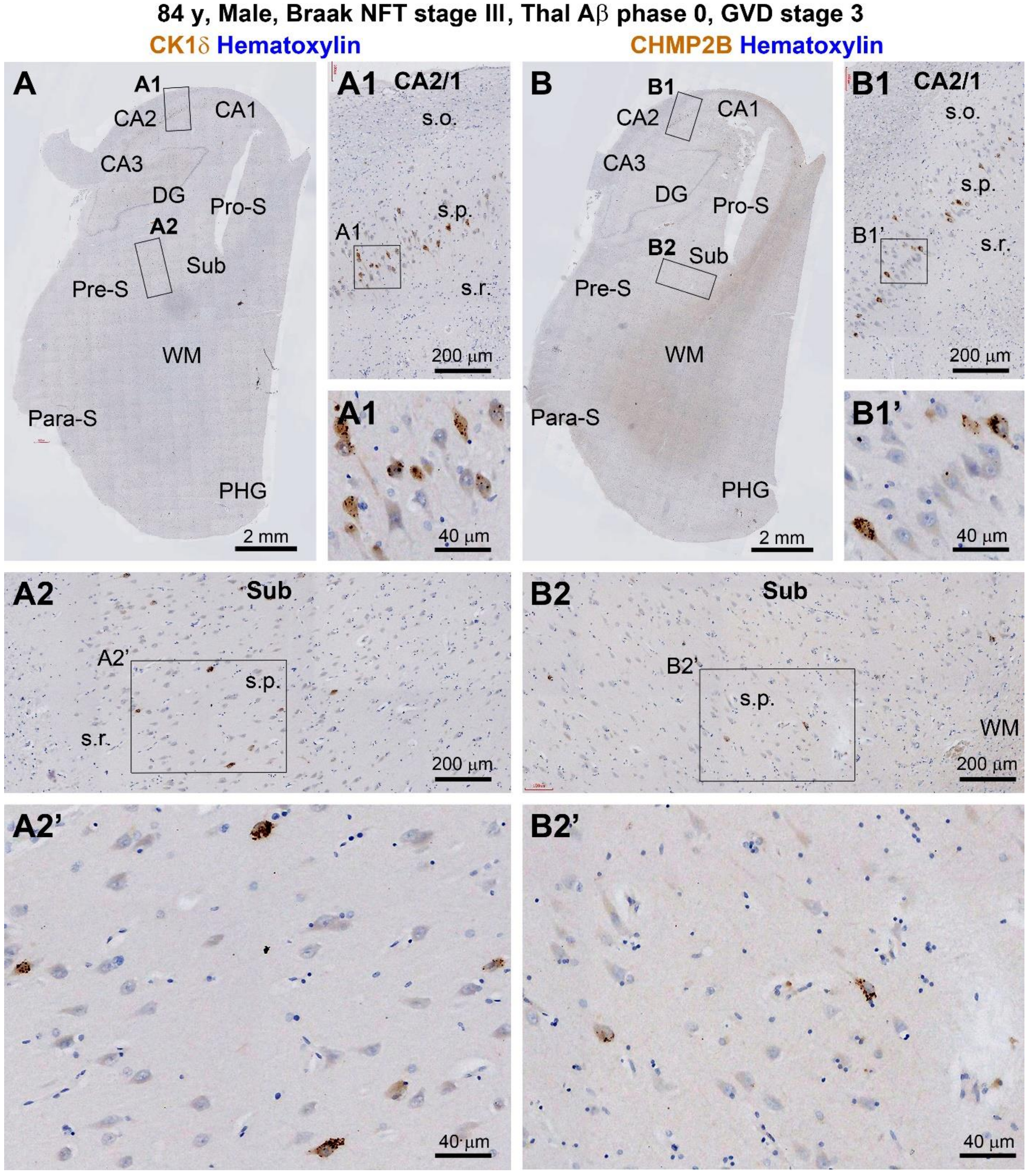

Supplemental Figure 7. Images showing granulovacuolar degeneration (GVD) immunolabeled by casein kinase I isoform δ (CK1δ) and charged multivescular body protein 2B (CHMP2B) in adjacent paraffin sections from a case with primary age-related tauopathy (PART) included in Supplemental Figure 4. (A1, A1') and (B1, B1') show the presence of many immunolabeled pyramidal neurons in the CA2 area. (A2, A2') and (B2, B2') show the presence of a few immunolabeled pyramidal neurons in the subiculum. At high magnification, the GVD bodies are darkly labeled and occurred in varying numbers in the immunolabeled neurons. A diffuse and relatively light cytoplasmic reactivity is often seen in the neurons with densely packed GVD bodies.

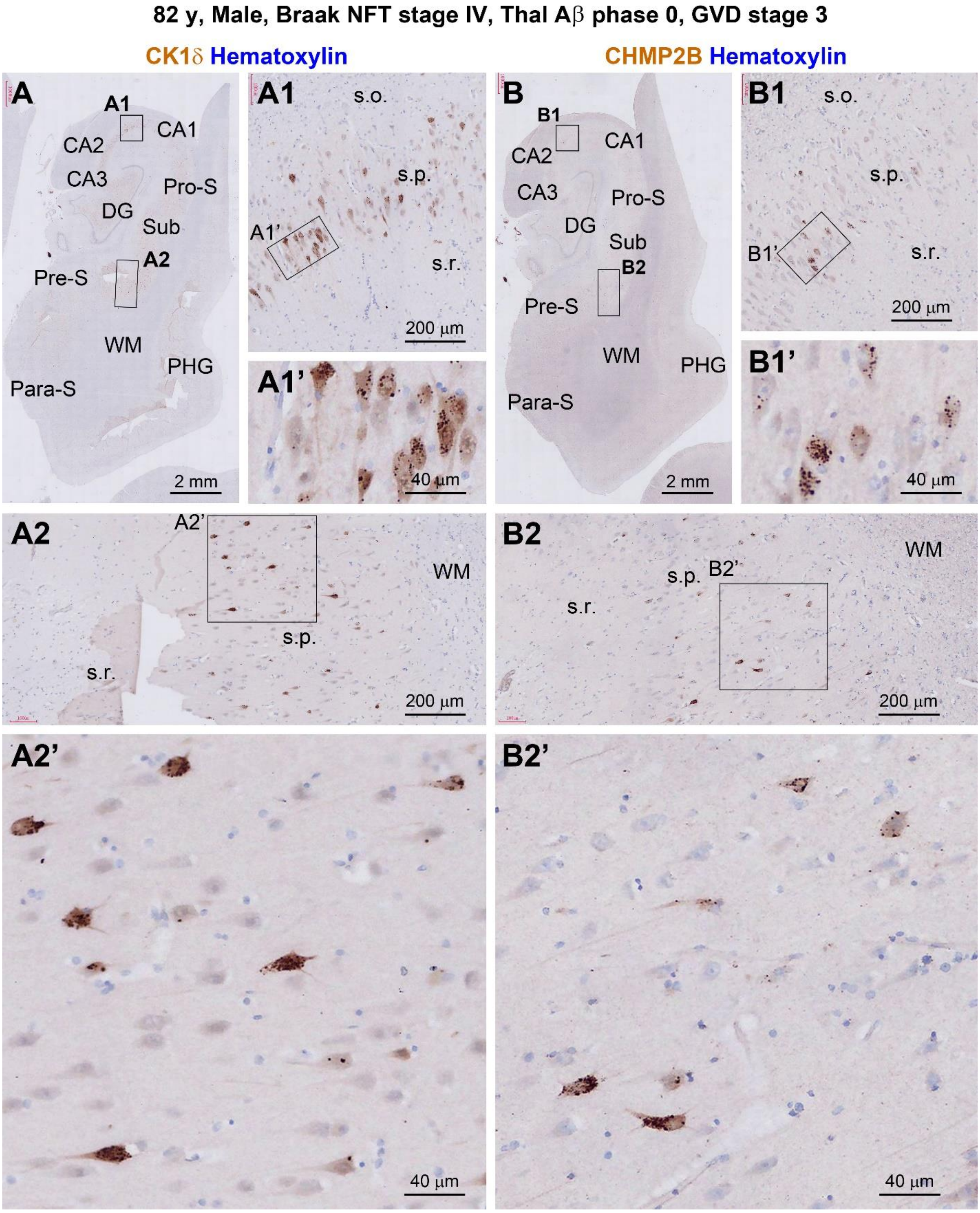

Supplemental Figure 8. Images showing granulovacuolar degeneration (GVD) immunolabeled by CK1δ and CHMP2B in adjacent paraffin sections from another case (#35) with primary age-related tauopathy (PART). (A, A1, A1', B, B1, B1') show the presence of a large subpopulation of immunolabeled pyramidal neurons in the CA1 area. (A2, A2') and (B2, B2') show the presence of many immunolabeled pyramidal neurons in the subiculum. At high magnification, the GVD bodies are densely packed in many of the immunolabeled neurons, in which diffuse cytoplasmic reactivity also exists in the somata and dendritic processes.

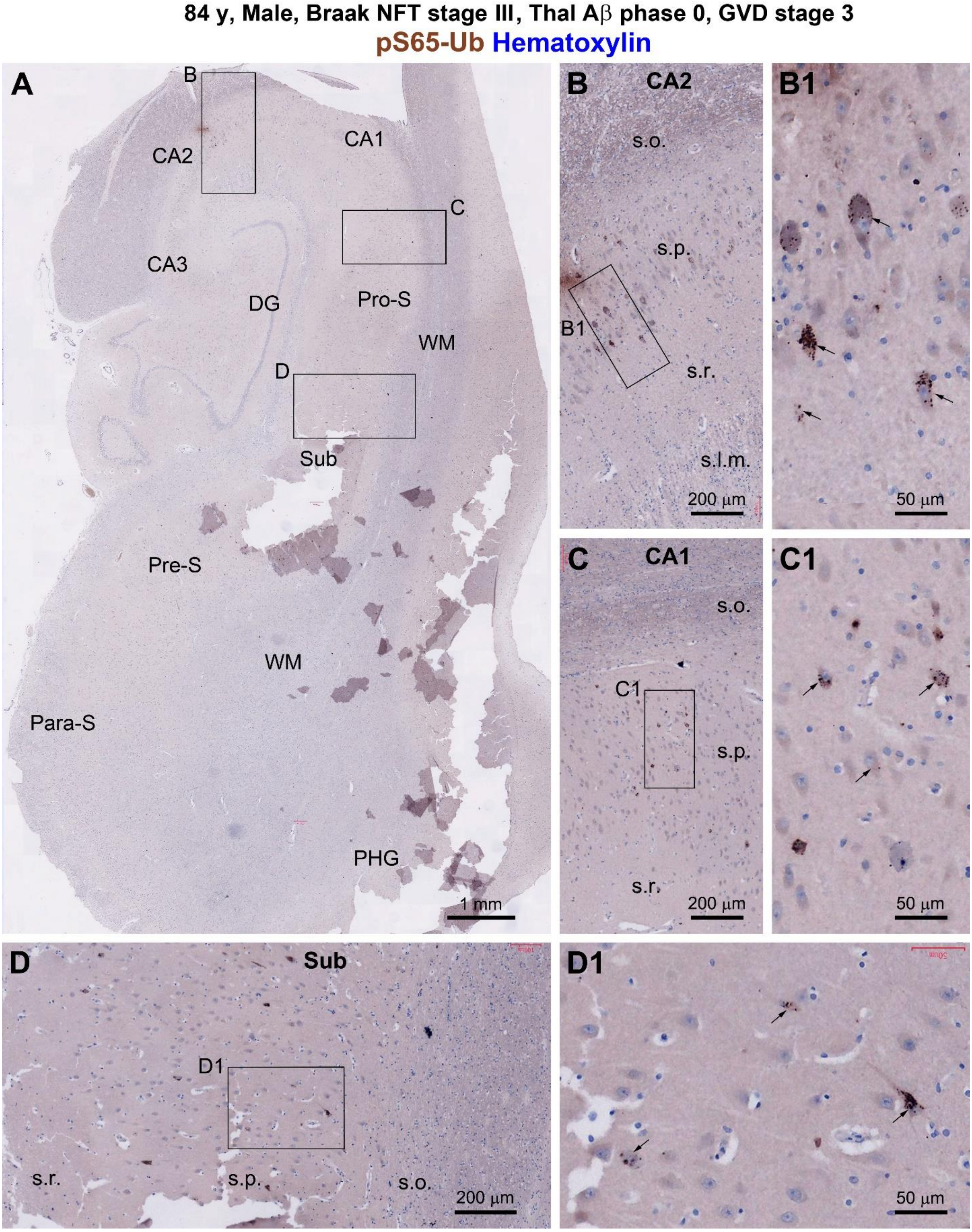

Supplemental Figure 9. Images showing an example of PINK1-generated phospho-ubiquitin (pS65-Ub) labeling of granulovacuolar degeneration (GVD) in paraffin sections from a case (#34) with primary age-related tauopathy (PART). Framed areas in (A) are enlarged as other panels as indicated. Immunolabeled GVD bodies (pointed by arrows) are found in a subpopulation of pyramidal neurons in all subicular and CA1 to CA2 sectors. Neurons with dense GVD bodies also often show light and diffuse cytoplasmic immunoreactivity.

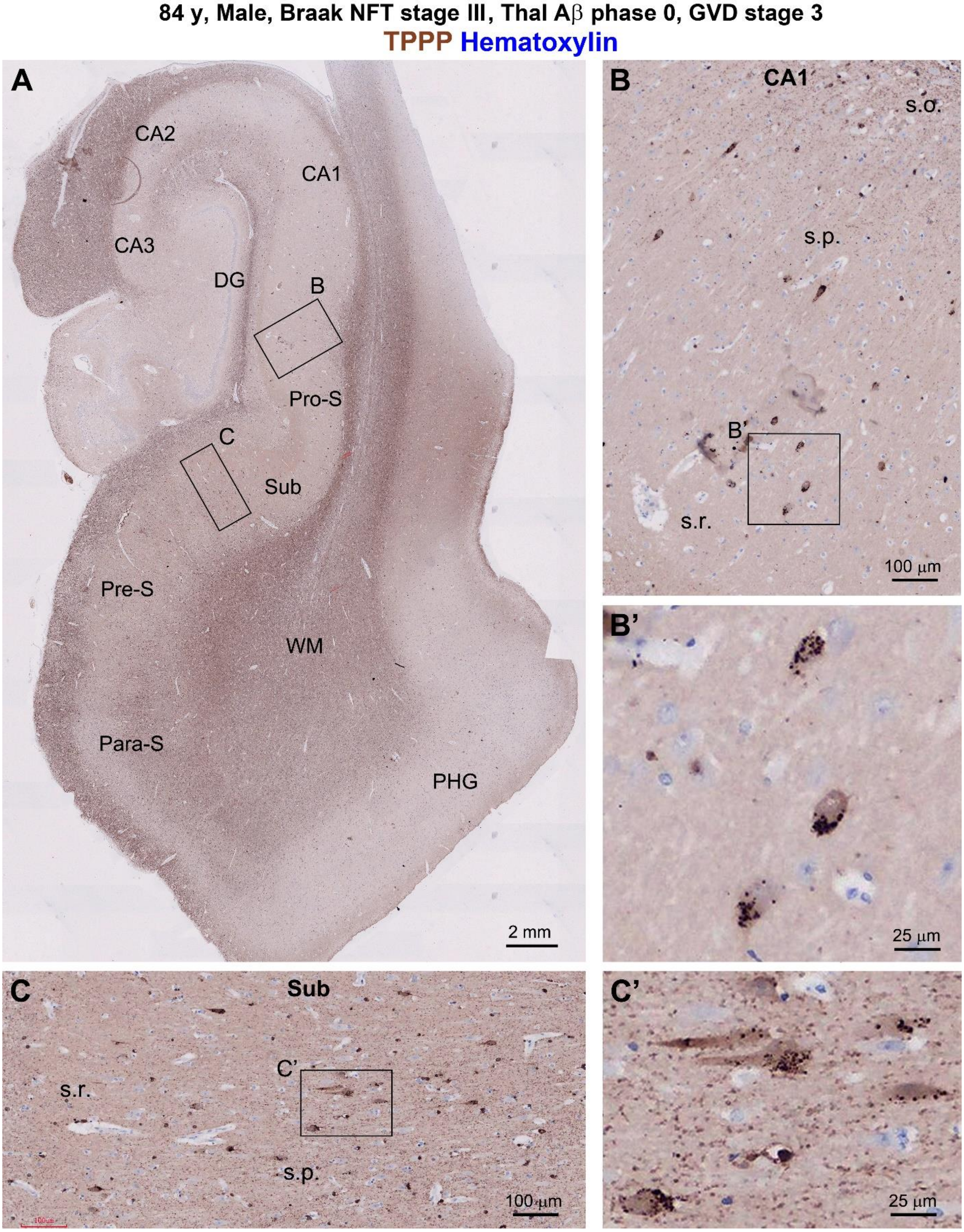

Supplemental Figure 10. Images showing an example of tubulin polymerization promoting protein (TPPP) labeling of granulovacuolar degeneration (GVD) in paraffin sections from the PART case presented in Supplemental Figure 9. Framed areas in (A) are enlarged as other panels as indicated (B-C'). Immunolabeled GVD bodies are found in a subpopulation of pyramidal neurons in all subicular and CA1 to CA2 sectors. Light and diffuse cytoplasmic immunoreactivity is seen in neurons with dense GVD bodies.

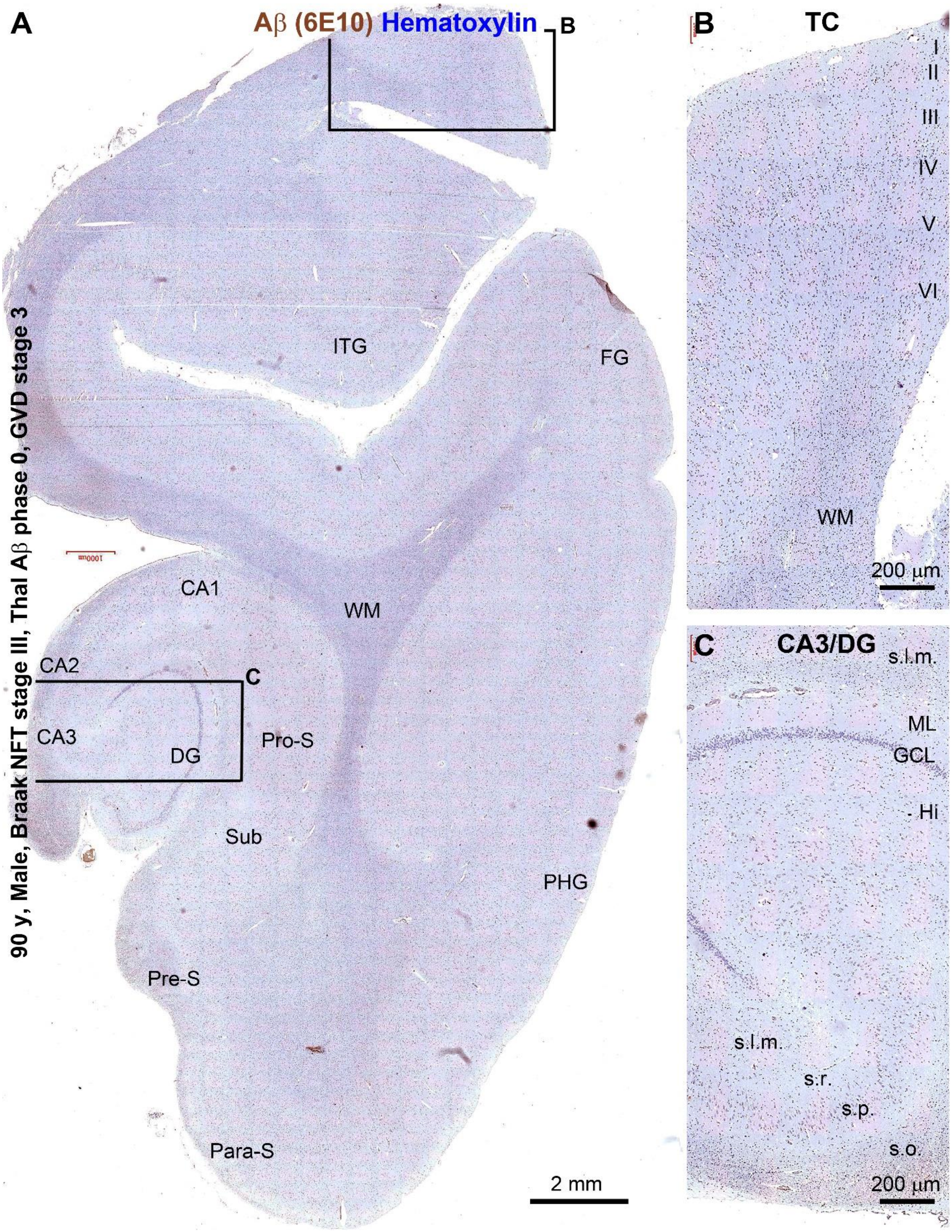

Supplemental Figure 11. Images showing the lack of β-amyloid deposition (Aβ) in temporal lobe paraffin section from an additional case (#30) with primary age-related tauopathy (PART). Other labelings prepared in adjacent sections from this same case are illustrated as Supplemental Figures 12-19. Framed areas in (A) are enlarged as (B, C), illustrating the absence of Aβ labeling in the neocortex as well as the hippocampal formation. Abbreviations are as defined in Supplemental Figures 1 and 5.

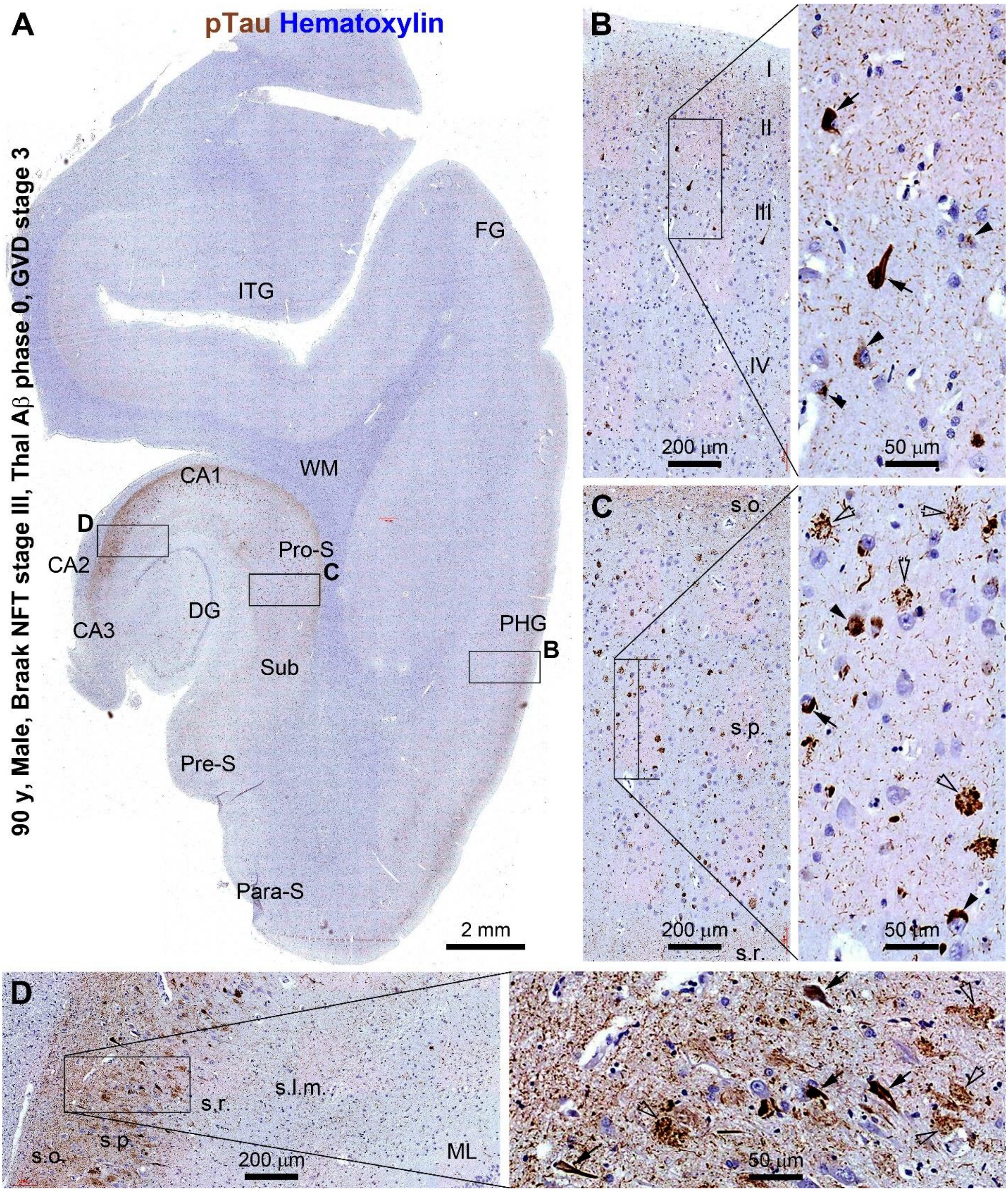

Supplemental Figure 12. Images showing phosphorylated tau (pTau) immunolabeling in an adjacent paraffin section to that of Aβ labeling in Supplemental Figure 11 from the same PART case (#30). Fairly abundant pTau reactivity is seen in the hippocampal formation including in the dentate gyrus (DG). High magnification views of labeled neuronal somata and processes in CA2, prosubiculum (Pro-S) and the parahippocampal gyrus (PHG) are illustrated as indicated in panels (B-D) and enlarged inserts. Some neurons are packed with tangles (pointed by arrows), while others contained granular elements inside the somata (arrowheads). Small neuritic clusters are also observed (open arrows). Other abbreviations are as defined in Supplemental Figure 1.

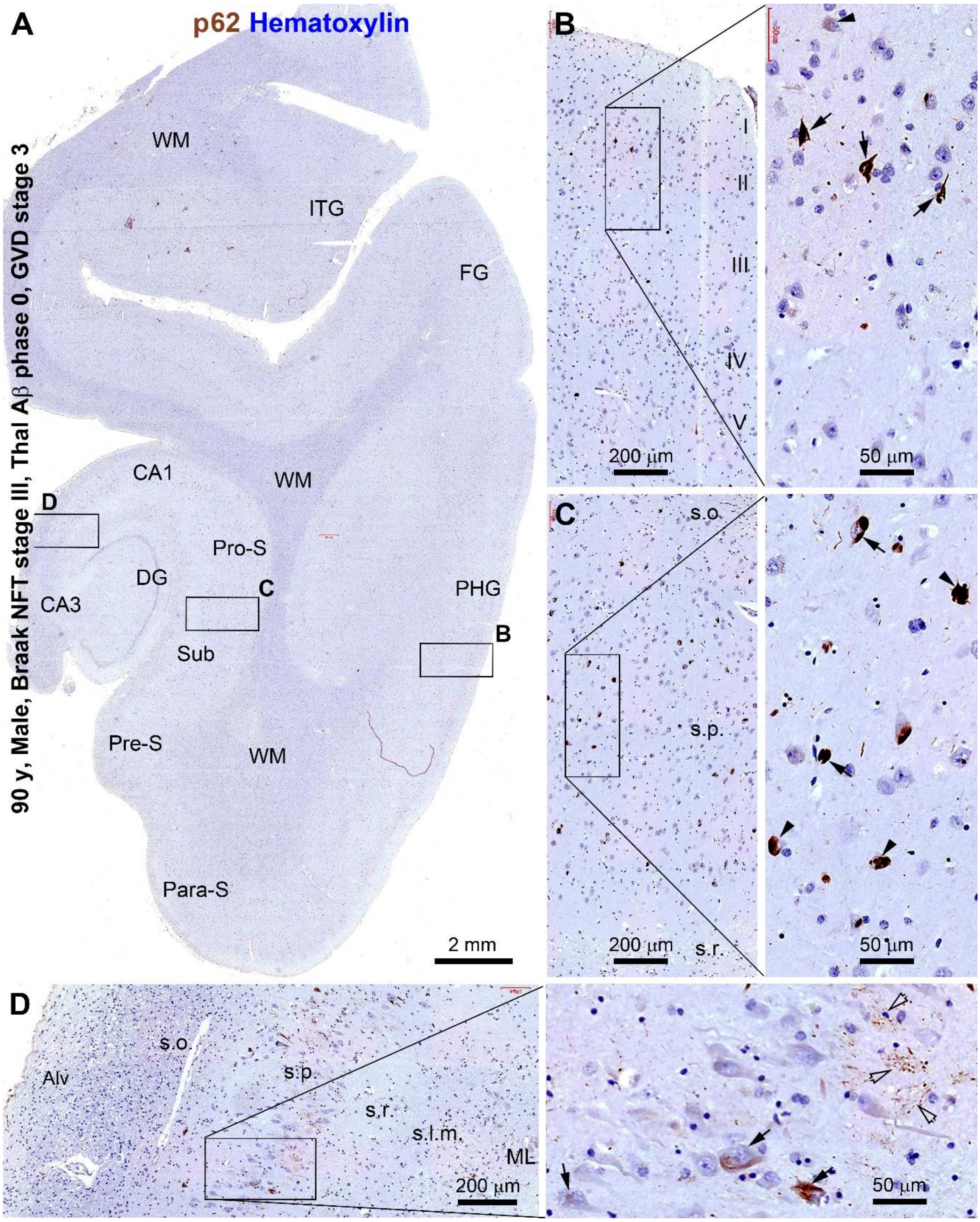

Supplemental Figure 13. Images showing p62 immunolabeling in temporal lobe paraffin section from the same PART case (#30) as with Supplemental Figures 11-12. p62 immunoreactivity is seen in the temporal neocortex (B) and the hippocampal formation (C, D). High magnification views of labeled neuronal somata and processes in CA2 (D), prosubiculum (Pro-S) and the parahippocampal gyrus (PHG) are illustrated as panels enlarged from the framed areas in (B, C, D). Some labeled neurons contain tangle-like structures (pointed by arrows), while others contained granular elements inside the somata (arrowheads). In addition, immunolabeled neuritic elements are observed in the neocortex, subiculum and CA2 (pointed by open arrows). Other abbreviations are as defined in Supplemental Figures 1 and 5.

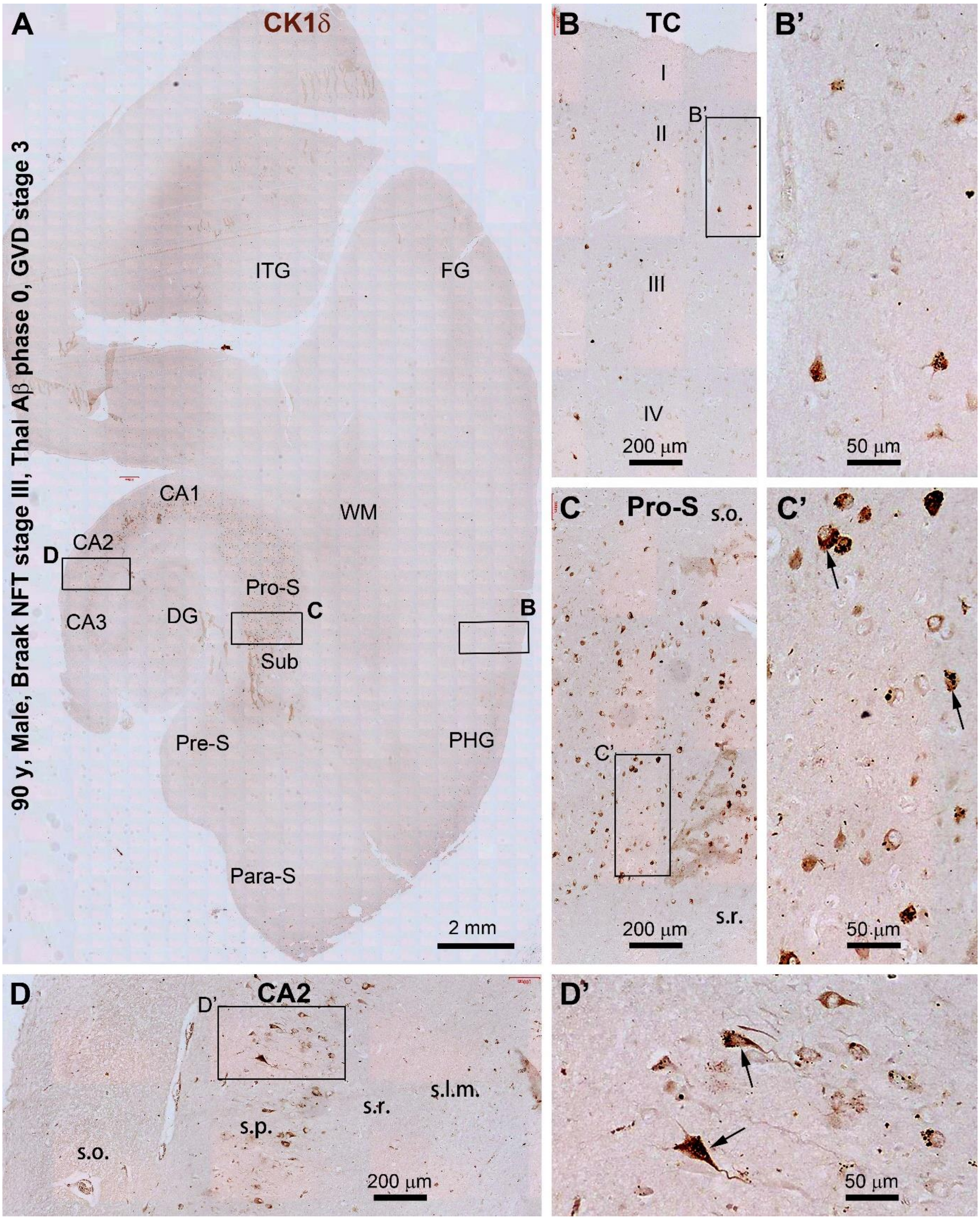

Supplemental Figure 14. Images showing casein kinase 1 delta (CK1δ) immunoreactivity (IR) in temporal lobe paraffin section from the PART case #30. CK1δ IR is seen in a small number of neocortical pyramidal neurons (B, B'), but a large population of subicular (C, C') and hippocampal (D, D') pyramidal neurons. The number of immunoreactive intraneuronal GVD bodies varies from one per cell in a single neuron, to numerous in a given neuron that are densely packed in the somata and proximal dendrites. Cytosol labeling appears and increases in intensity as the number of GVD bodies in the neurons increases (C', pointed by arrows), which can reach to a level such that the entire neuron exhibits tangle-like structures (D', pointed by arrows).

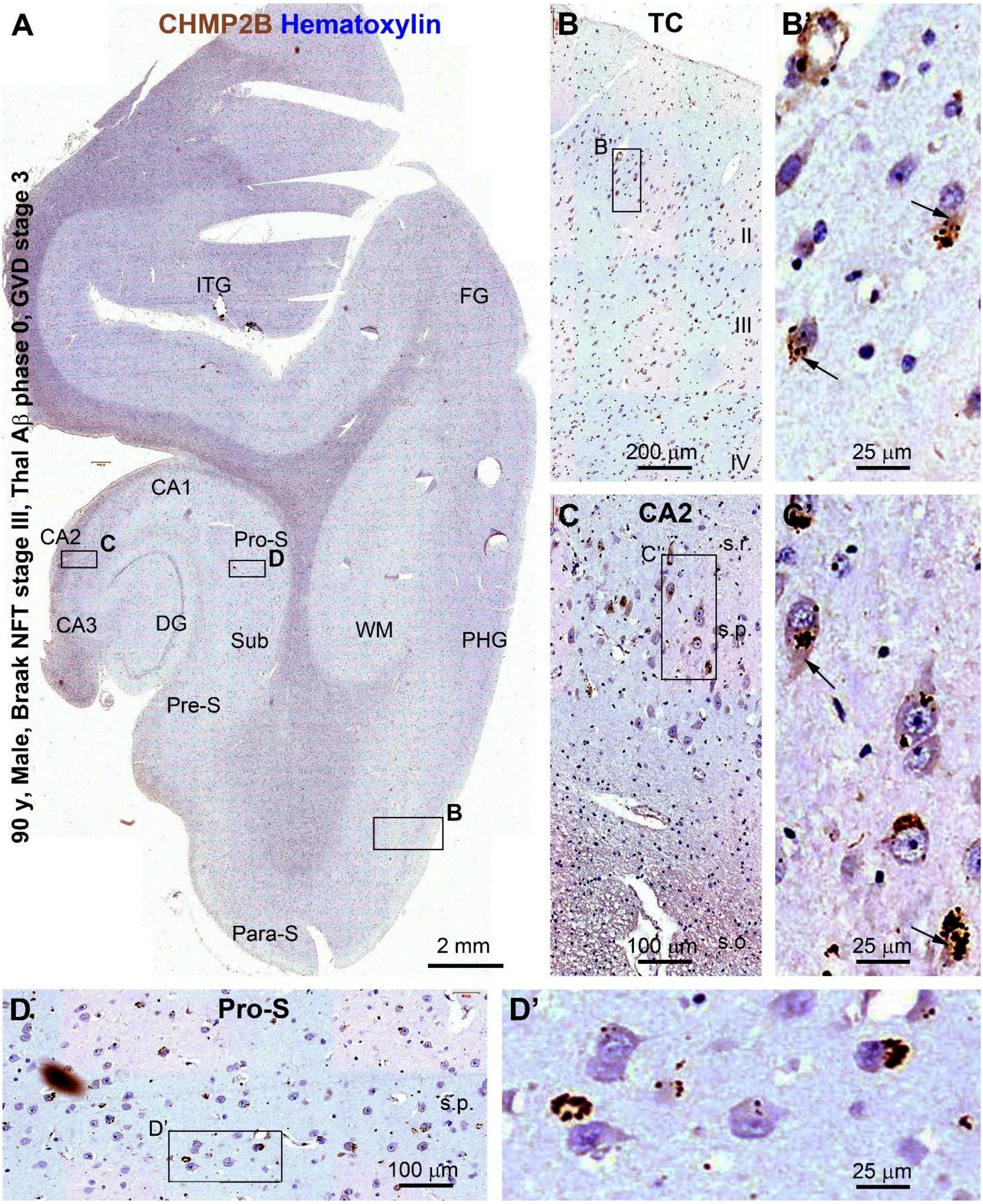

Supplemental Figure 15. Images showing immunoreactivity (IR) of charged multivesicular body protein 2B (CHMP2B) in temporal lobe paraffin section from the PART case #30. CHMP2B IR is seen in a few cortical pyramidal neurons (B, B'), but many hippocampal (C, C') and subicular (D, D') pyramidal neurons. The number of immunoreactive intraneuronal GVD bodies in a given neuron varies from a few to numerous. In the latter case the GVD bodies can be densely packed. Cytosol CHMP2B labeling is often seen in the neurons with a relatively large number of GVD bodies (B', C', pointed by arrows).

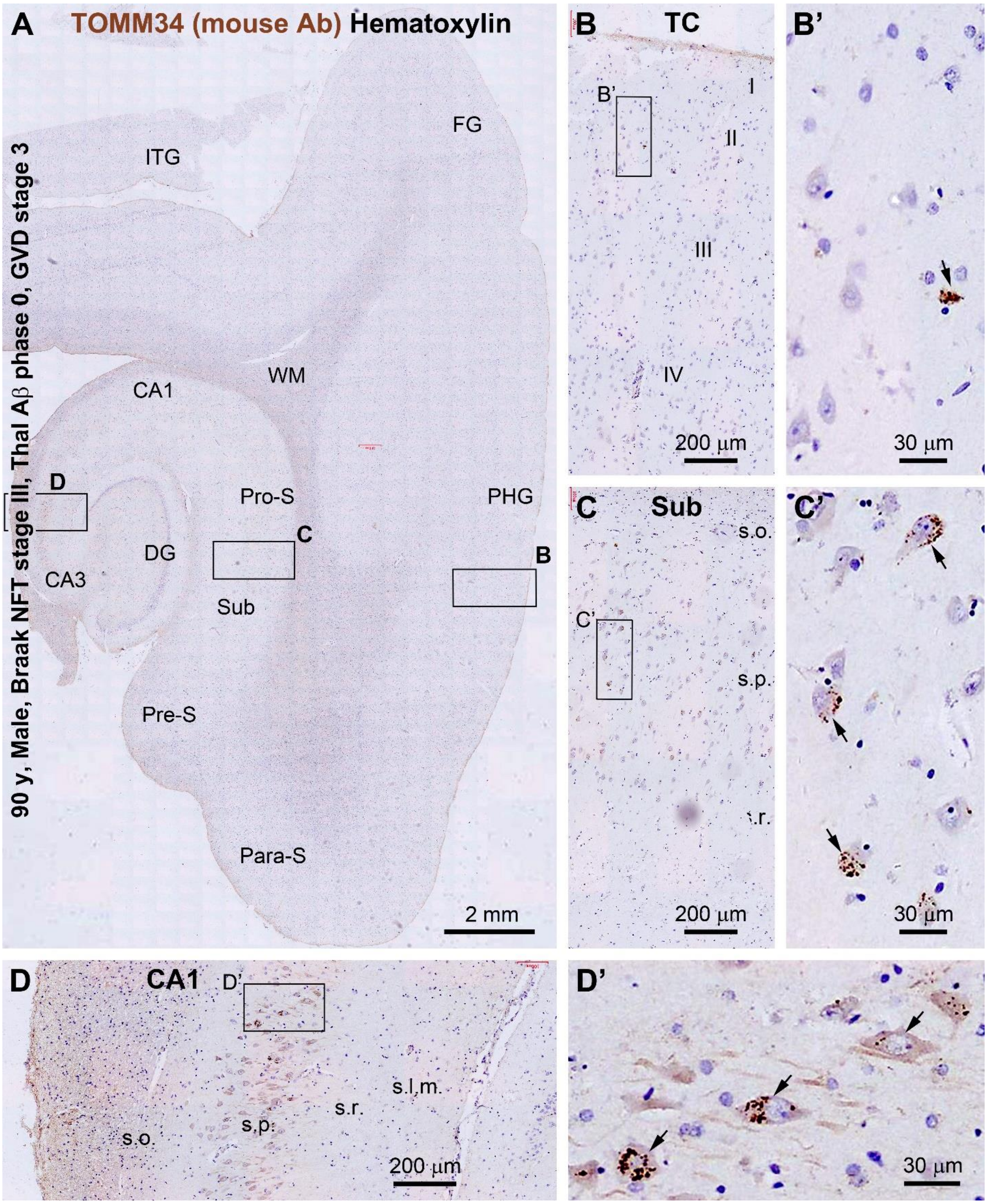

Supplemental Figure 16. Images showing the immunoreactivity (IR) of the 34 kDa-translocase of the outer mitochondrial membrane (TOMM34) as revealed by the mouse antibody in temporal lobe paraffin section from the PART case #30. TOMM34 immunoreactive granules are seen in a few cortical pyramidal neurons (B, B'), but in more subicular (C, C') and hippocampal (D, D') pyramidal neurons (pointed by arrows). The number of the intraneuronal granules in a given neuron can vary from a few to numerous such that they are densely packed. Cytosol immunoreactivity is seen in the somata and dendrite-like processes of some TOMM34 labeled neurons containing the GVD bodies (D').

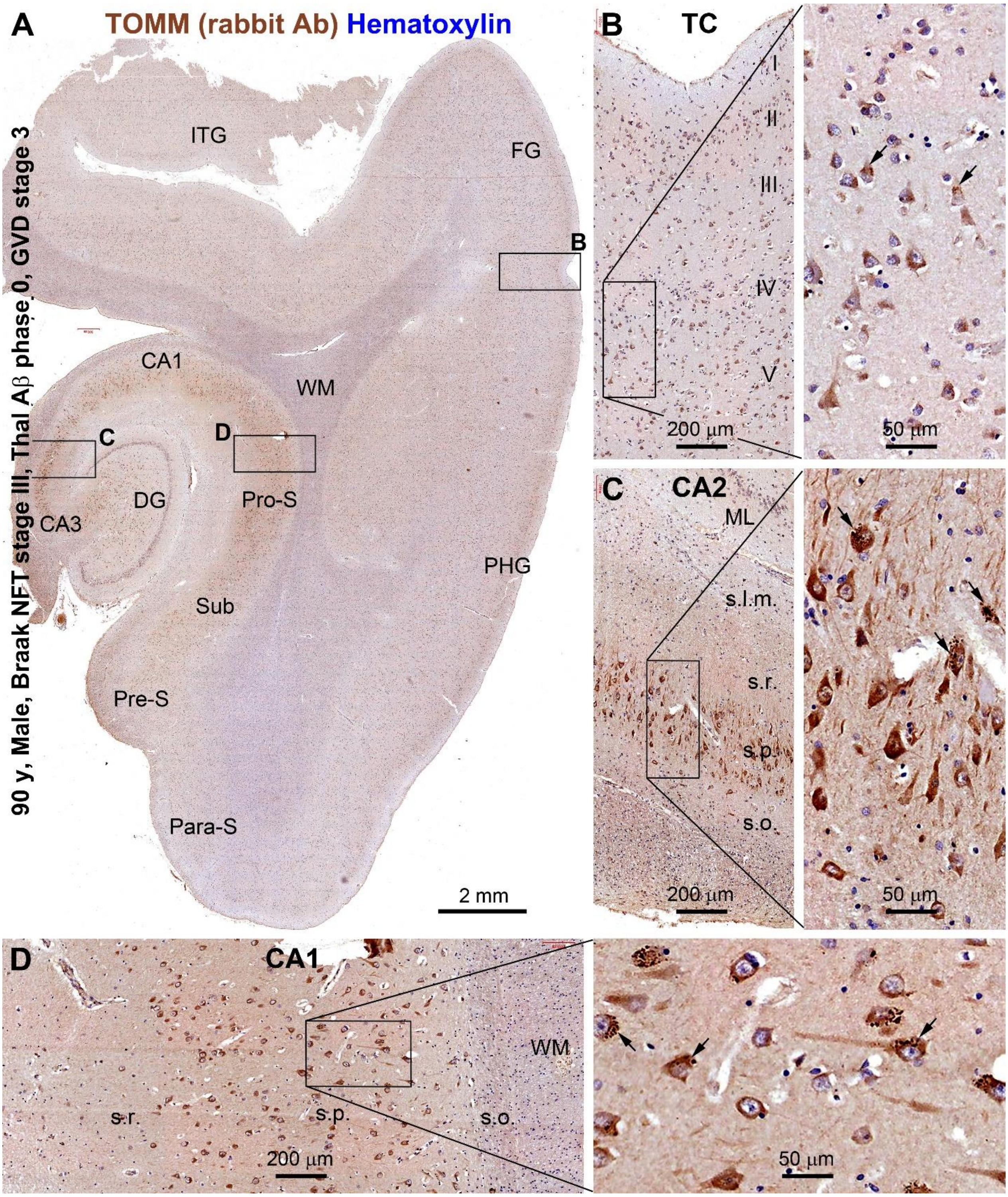

Supplemental Figure 17. Images showing the immunoreactivity (IR) of the 34 kDa-translocase of the outer mitochondrial membrane (TOMM34) as revealed by a rabbit antibody in temporal lobe paraffin section from the PART case #30. Cytosol TOMM34 IR is seen in the somata and dendrites in many neocortical pyramidal neurons (B and enlarged area), but in most subicular (C and enlarged area) and hippocampal (D enlarged area) pyramidal neurons (pointed by arrows), while darkly stained GVD bodies are present in these labeled neurons. The number of immunoreactive intraneuronal GVD bodies in a given single neuron can also vary from a few to numerous, which in the latter cases are often densely packed.

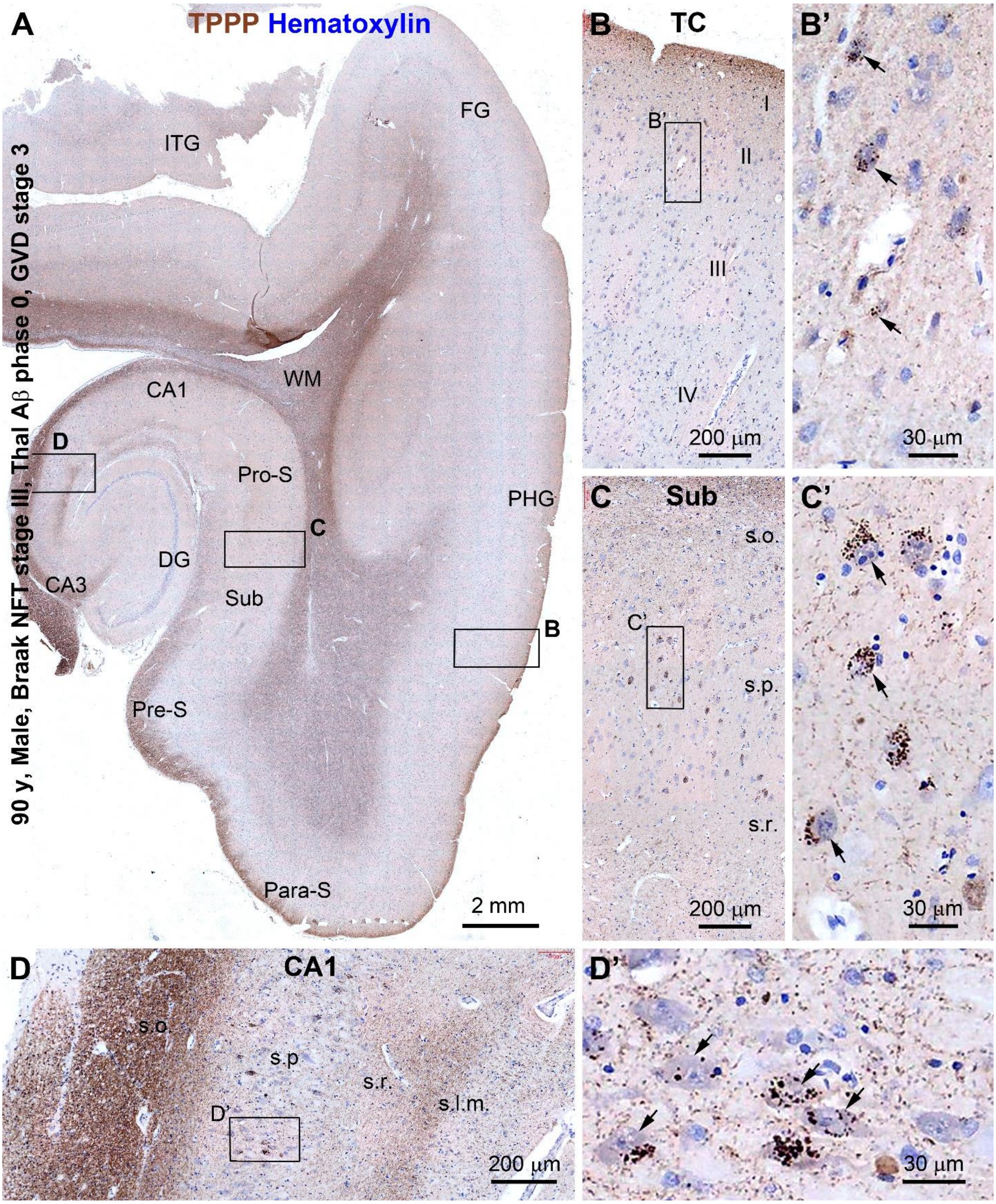

Supplemental Figure 18. Images showing the immunoreactivity (IR) of the tubulin polymerization promoting protein (TPPP) in temporal lobe paraffin section from the PART case #30. TPPP IR is seen in a few neocortical pyramidal neurons (B, B'), and many subicular (C, C') and hippocampal (D, D') pyramidal neurons (pointed by arrows). The number of immunoreactive intraneuronal granules representing GVD bodies in a single neuron varies from a few to numerous that are densely packed. There exists fairly dense fine terminal labeling in the stratum oriens (s.o.) and the white matter (WM) (A, D) and discrete dot-like labeling in the grey matter (C' and D'), which is suggestive of a localization of IR to axonal terminals.

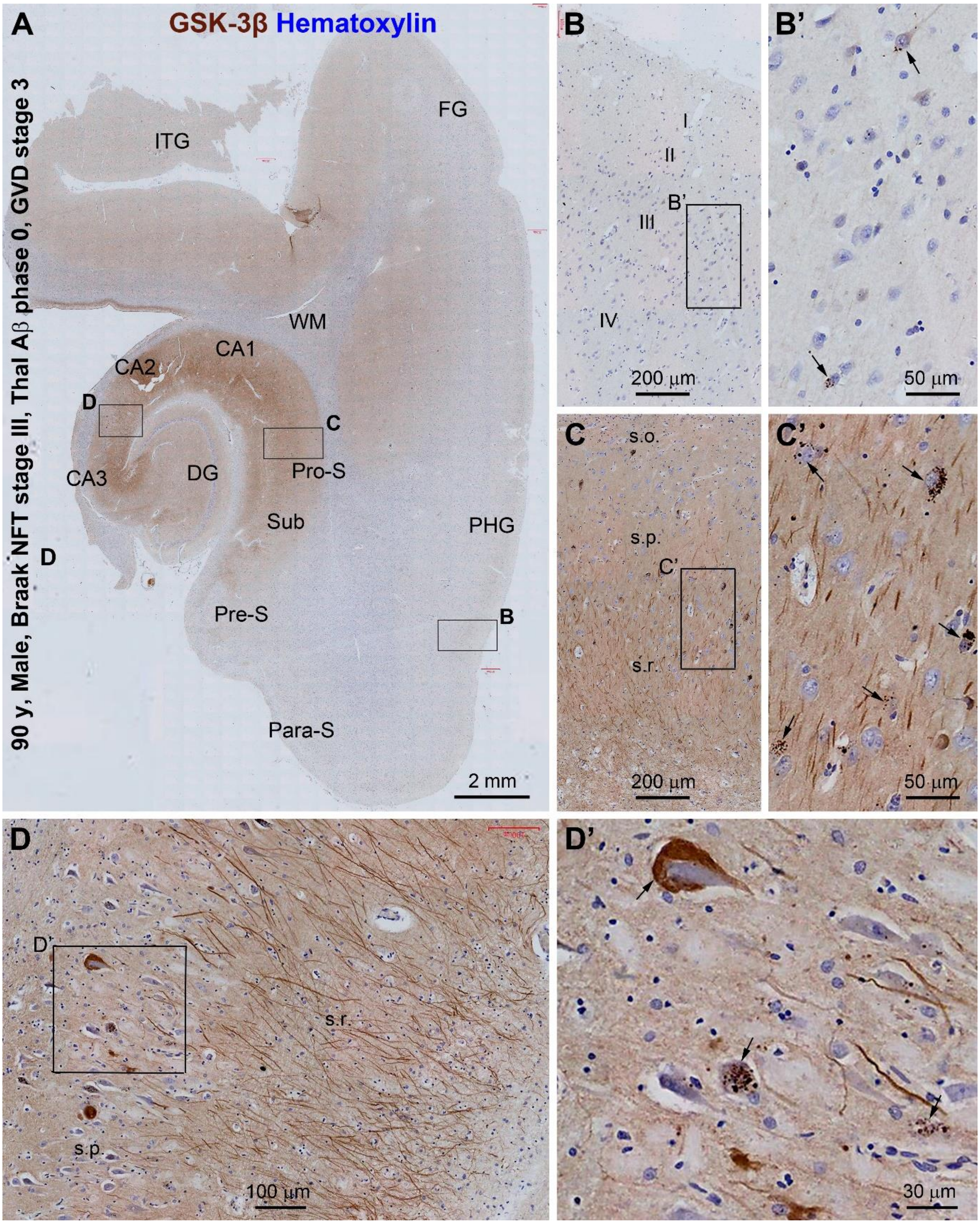

Supplemental Figure 19. Images showing the immunoreactivity (IR) of glycogen synthase kinase-3 isoform β (GSK3β) in temporal lobe paraffin section from the PART case #30. A few pyramidal neurons are labeled in the parahippocampal gyrus (PHG) (B, B'), while a considerable subpopulation of subicular (C, C') and hippocampal (D, D') pyramidal neurons are labeled. Intraneuronal granules varies from a few to densely packed among individual neurons (pointed by arrows). A few pyramidal neurons exhibit cytosol labeling such that the whole cell appeared as tau positive neurons packed with tangles (D, D'), The apical dendrites of the subicular and pyramidal neurons are distinctly labeled (C, C', D, D').

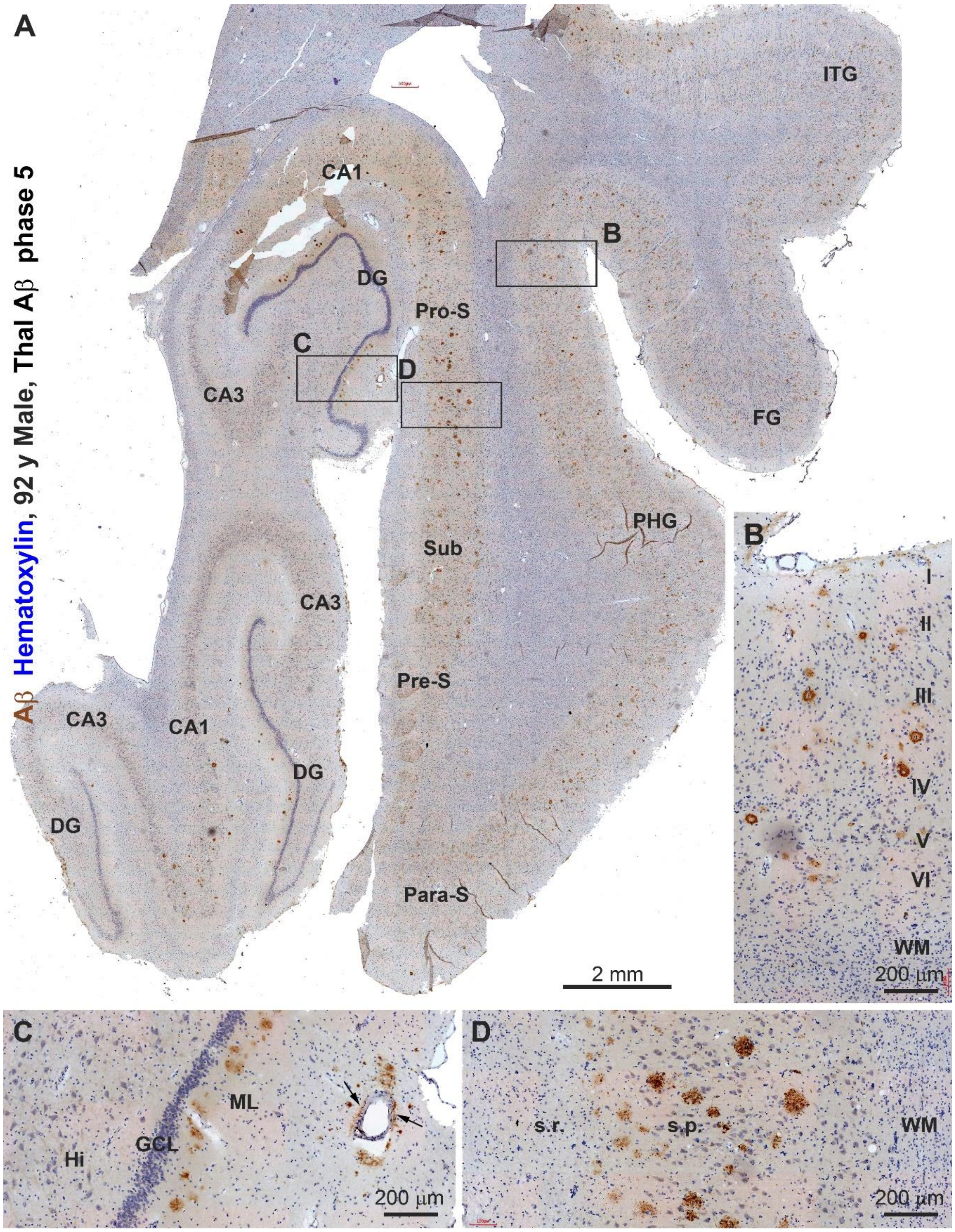

Supplemental Figure 20. Immunoreactivity (IR) of β-amyloid (Aβ) stained with the 6E10 antibody in a temporal lobe paraffin section from the brain of case #50 from the pAD/AD group. Neuropathologies labeled by additional representative antibody markers in adjacent sections are shown in the following Supplemental Figures (21-23). Extensive Aβ deposition occurs across the neocortical and entorhinal cortices and hippocampal formation. In high magnification views (B-D) as enlarged from the boxed areas in (A). Aβ pathology can appear as compact plaques including the dense-cored form (B-D), diffuse plaques (A, e.g., subicular islands), cerebral amyloid angiopathy (C, pointed by arrows) and subpial Aβ deposition (B).

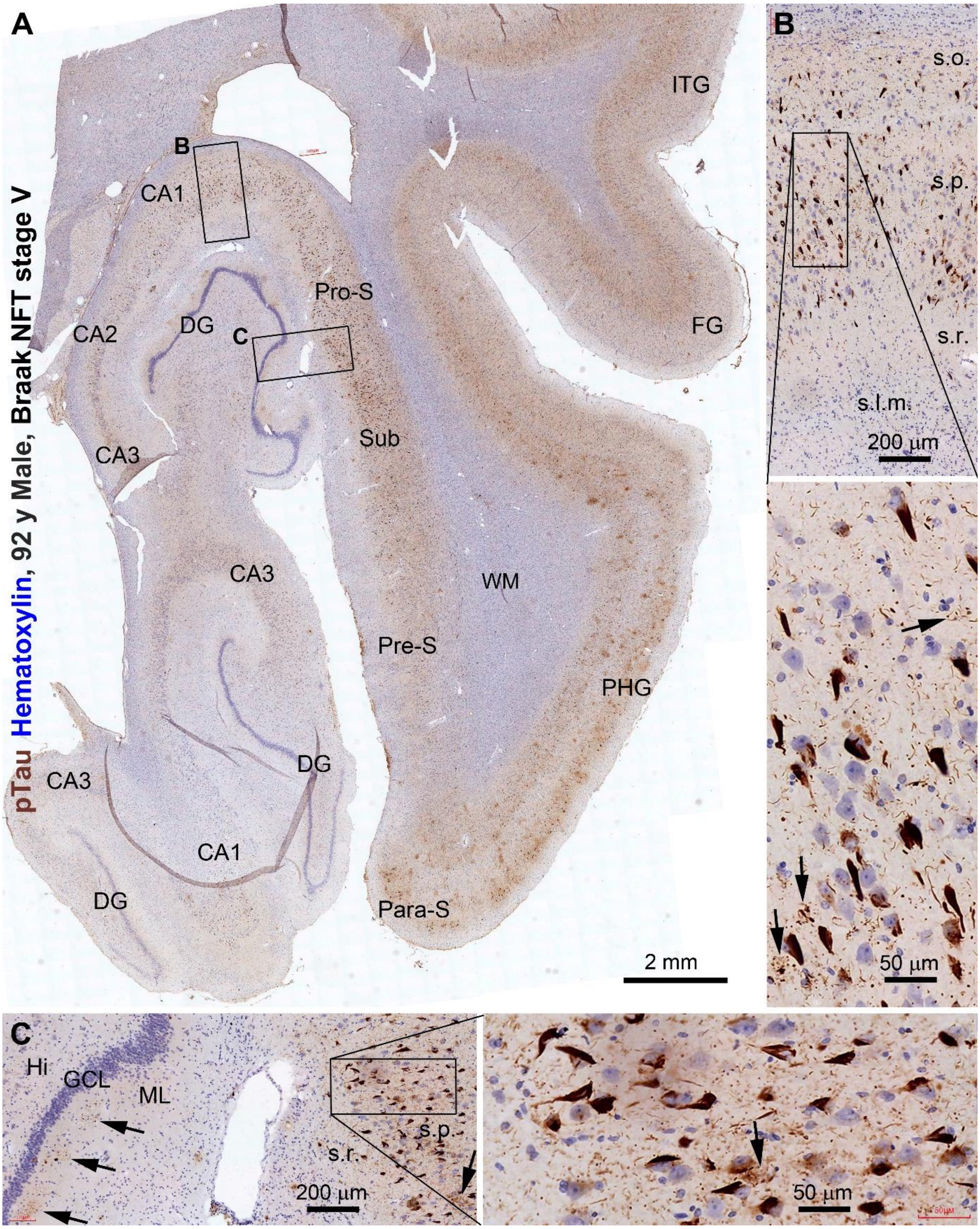

Supplemental Figure 21. Immunoreactivity (IR) of phosphorylated tau (pTau) in the temporal lobe section from the case #50. Extensive pTau IR occurs over the neocortical and entorhinal cortices, and hippocampal formation. In high magnification views (B, C) as enlarged from the boxed areas in (A), pTau IR occurs clearly in the somata and dendritic processes of pyramidal neurons, many of which contain heavily packed tangles and appear to be morphologically distorted. Neuritic profiles are also labeled, some of which are arranged in clusters representing plaque-associated dystrophic neurites, including in the molecular layer (ML) of the dentate gyrus (C, pointed by arrows).

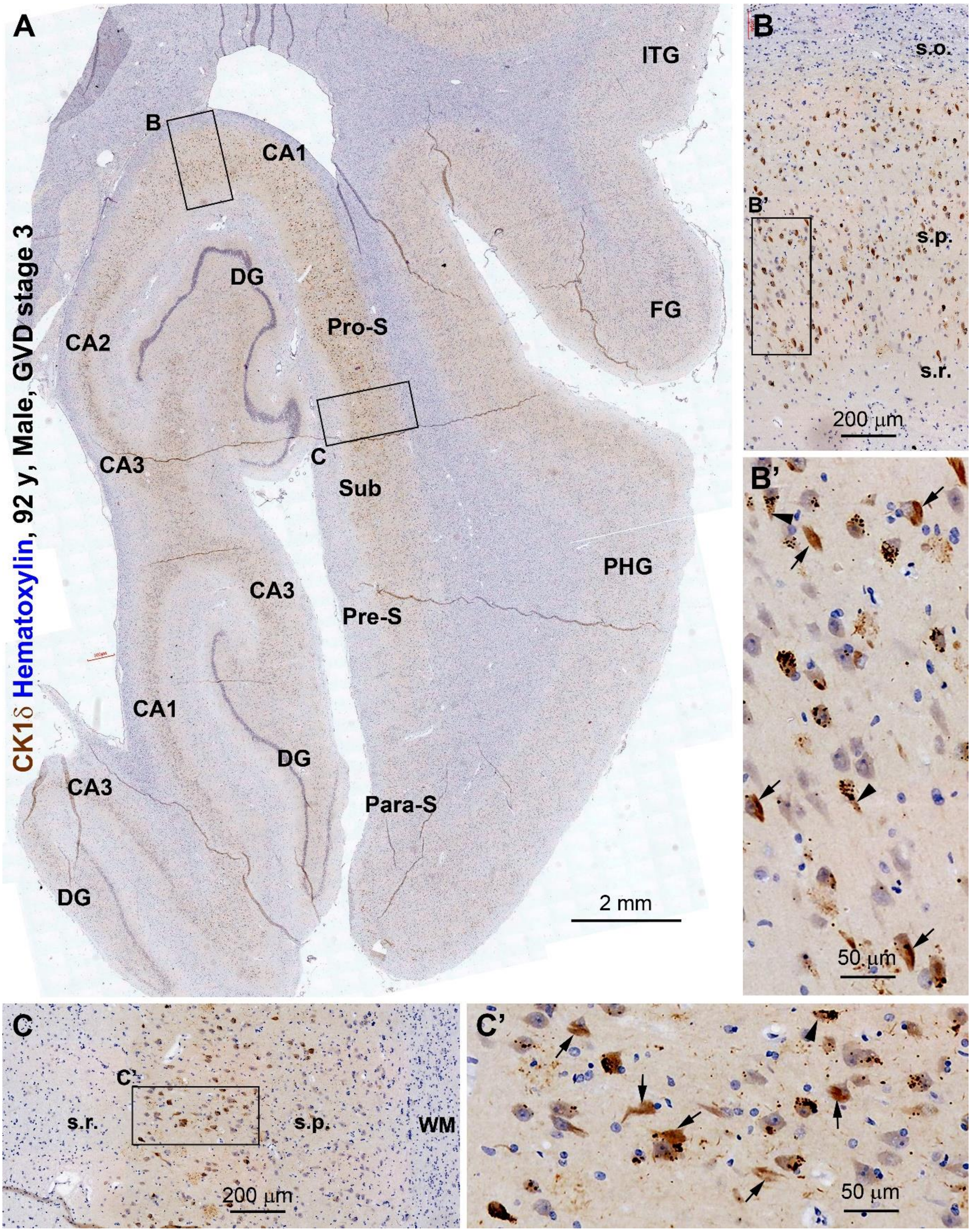

Supplemental Figure 22. Immunoreactivity (IR) of casein kinase 1 delta (CK1δ) in a temporal lobe section from case#50. Immunolabeled neurons are present across the subicular and hippocampal subregions. At high magnification (B, B', C, C'), the labeled intraneuronal granules representing GVD bodies are varied in number and labeling intensity among individual neurons. Cytosol labeling is seen in the neurons that have with densely packed granules (pointed by arrowheads). However, diffuse cytosol IR is also seen in neuronal profiles that are morphologically similar to tangle-filled pTau positive neurons, which otherwise contain fewer or even no CK1δ positive granules (pointed by arrows).

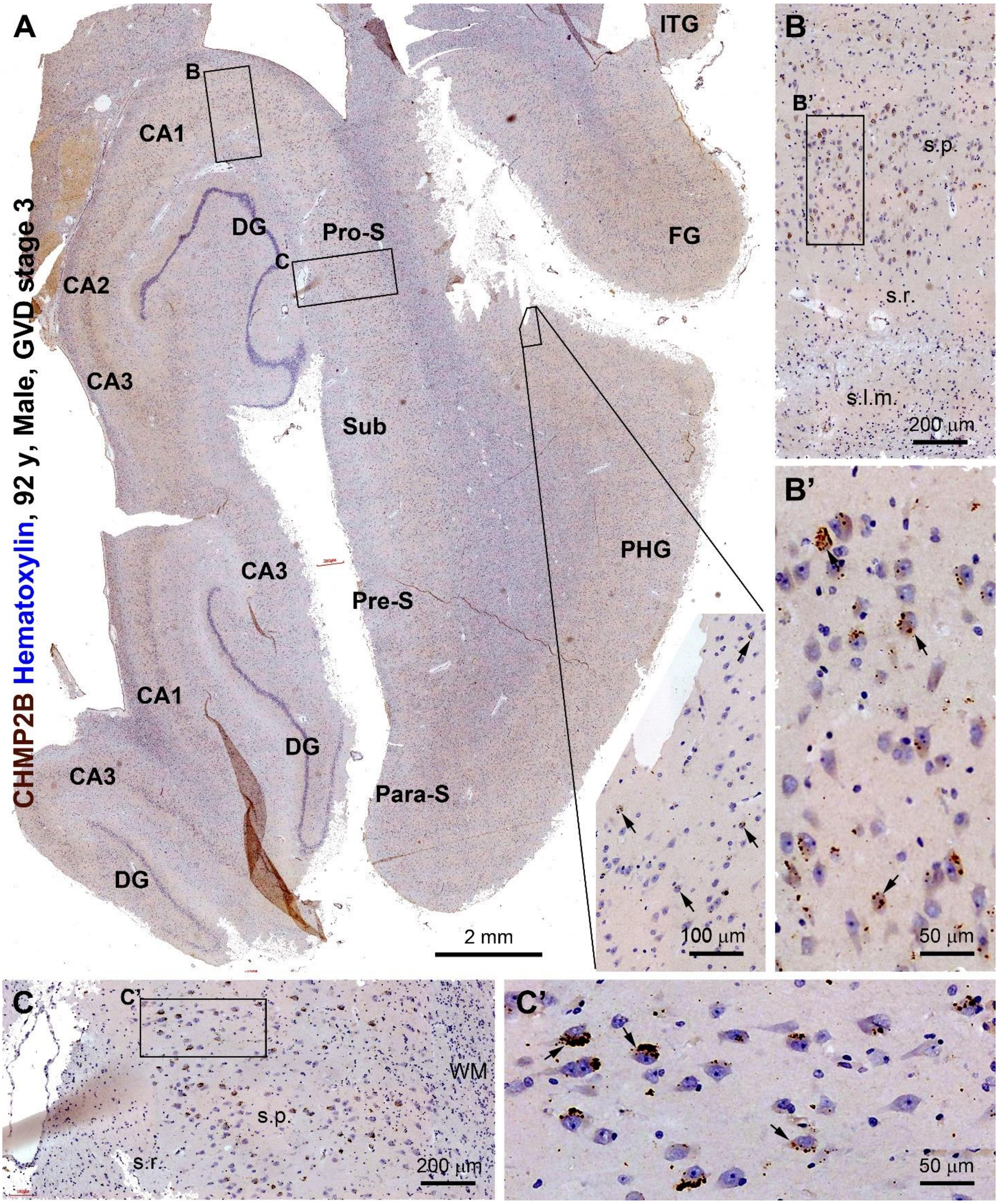

Supplemental Figure 23. Immunoreactivity (IR) of charged multivescular body protein 2B (CHMP2B) in a temporal lobe section from case#50. Immunolabeled neurons are largely present in the subicular subregions and hippocampal formation (B, B', C, C'), with fewer ones seen in temporal neocortex (insert in A). Labeled intraneuronal granules representing GVD bodies can vary dramatically in number among individual pyramidal neurons. Light cytosol labeling is also seen in some labeled neurons, more evident in those with densely packed granules.

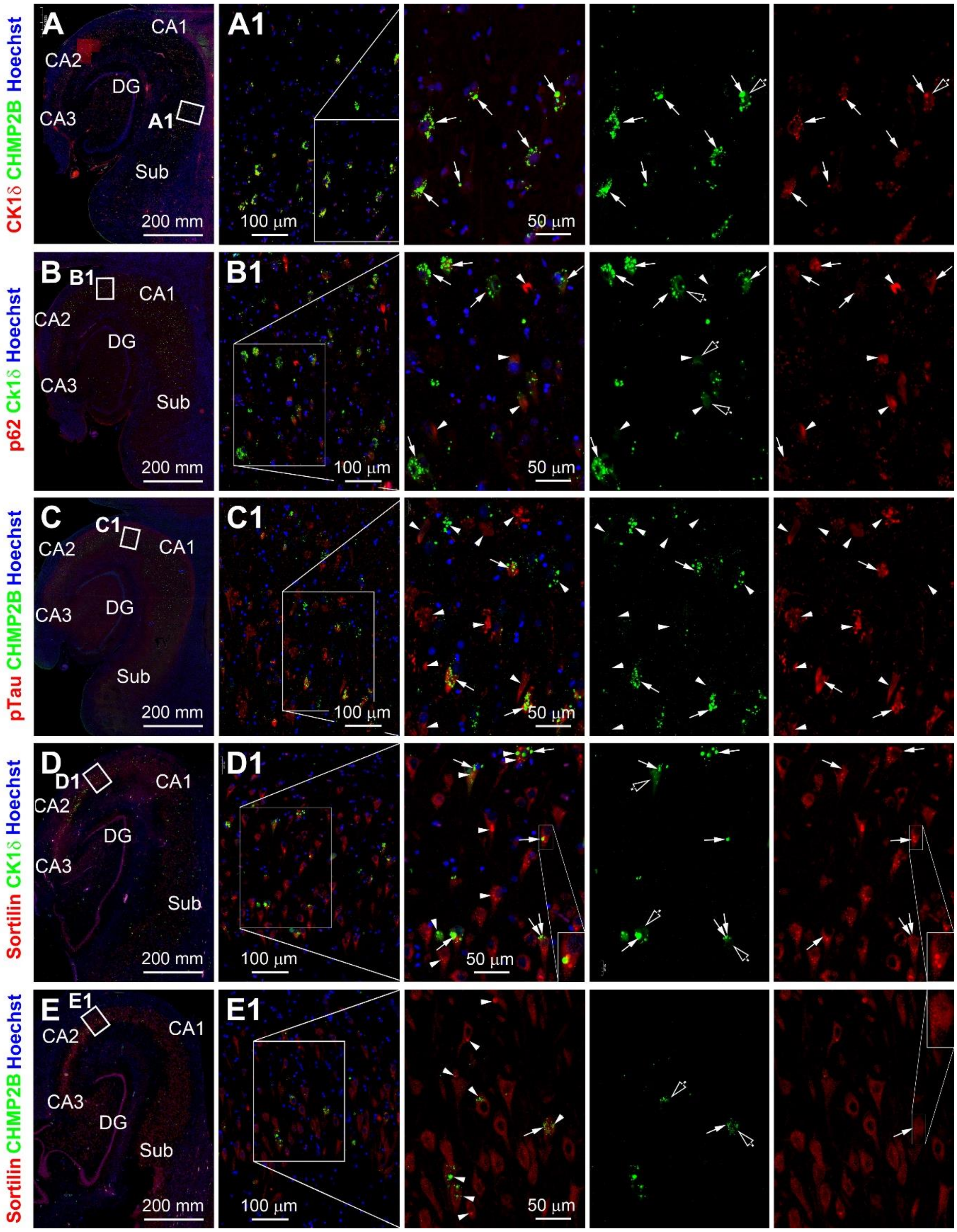

Supplemental Figure 24. Double immunofluorescence in temporal lobe paraffin sections from a PART case (#30). The antibody markers, nuclear dye Hoechst 33342 and image orientation are as indicated. Arrows point to colocalized profiles; open arrows to non-colocalized profiles. Ck1δ and CHMP2B labeling are extensively colocalized at granulovacuolar bodies (A, A1). Ck1δ and CHMP2B labeling are partially colocalized in p62 and pTau neurons. Heavily labeled p62 and pTau neurons (arrowheads) contain few or no Ck1δ/CHMP2B positive granules (B, B1, C, C1). Some sortilin labeled granules are colocalized with Ck1δ and CHMP2B labeling (D, D1, E, E1 and enlargements).

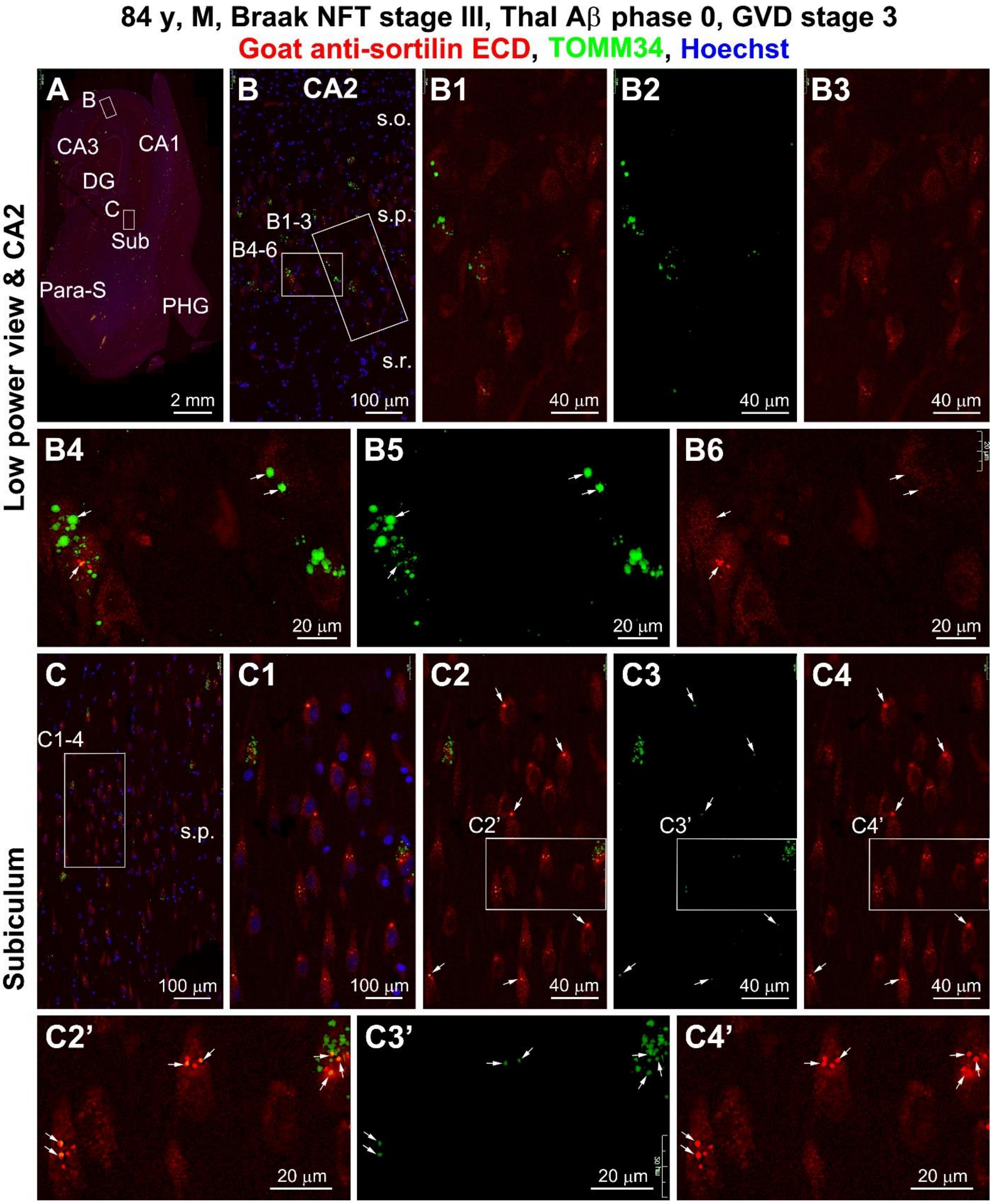

Supplemental Figure 25. Double immunofluorescent characterization of sortilin and the 34 kDa-translocase of the outer mitochondrial membrane (TOMM34) in temporal lobe paraffin sections from the PART case #34. The antibody/nuclear markers and image orientation are as indicated. The sortilin extracellular domain (ECD) antibody reveals fine granular elements as well as enlarged inclusion bodies in the hippocampal and subicular pyramidal neurons (B-B3, C-C4). TOMM34 immunoreactive GVD bodies occur in a subpopulation of sortilin labeled neurons. There is a partial colocalization of the two labeling among the intraneuronal aggregation bodies (B4-B6, C2'-C, arrows point to colocalized profiles).

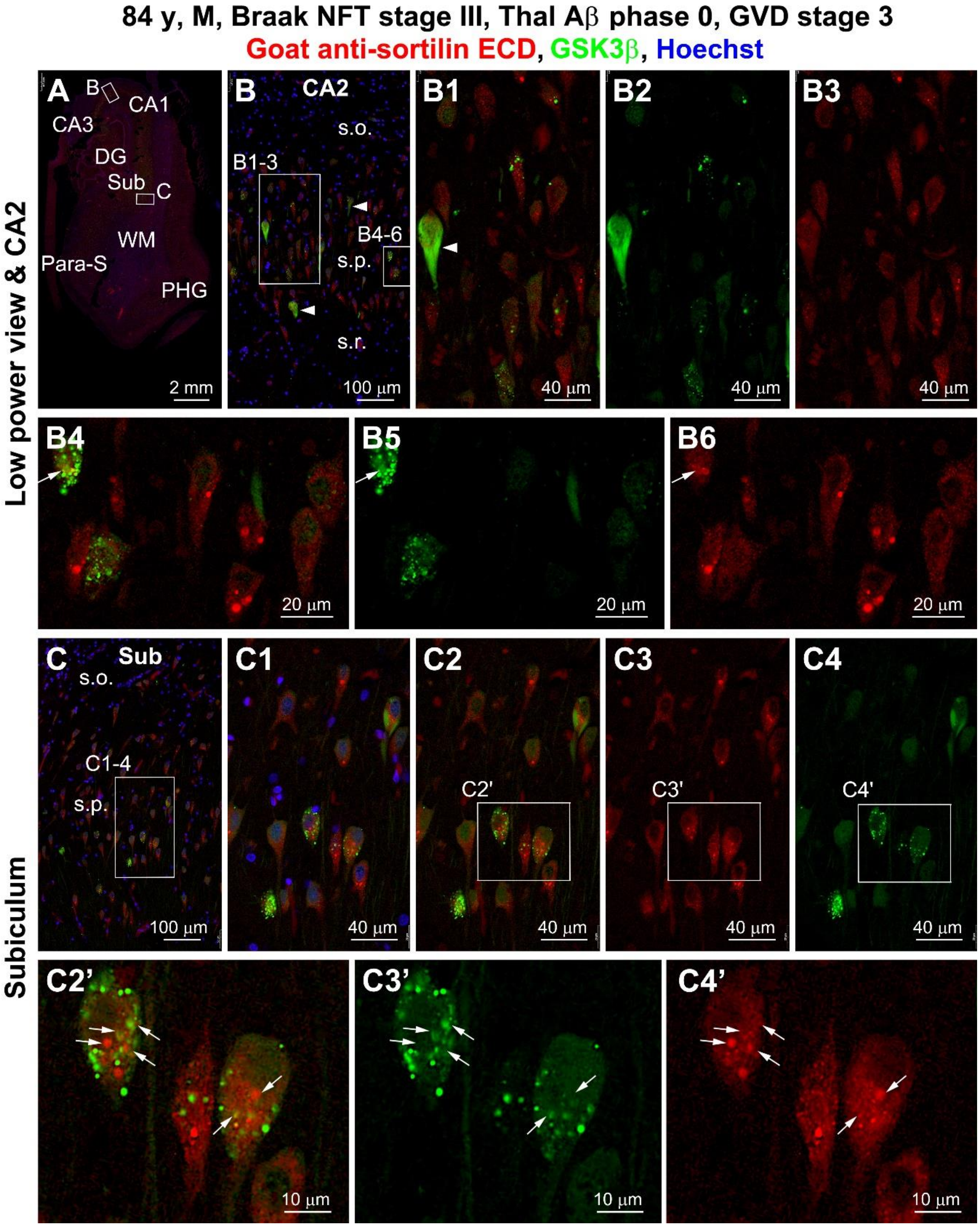

Supplemental Figure 26. Double immunofluorescent characterization of sortilin and glycogen synthase kinase-3 isoform β (GSK3β) in temporal lobe paraffin sections from the PART case #34. The antibody/nuclear markers and image orientation are as indicated. GSK3β and sortilin colocalization are visible at some aggregation bodies (pointed by arrows). Sortilin immunofluorescence appears to be reduced in these GSK3β positive GVD bodies, relative to the aggregates with bright sortilin immunofluorescence (B4-B6, C2'-C4'). Notably, spreading cytosol GSK3β labeling is seen in the neurons densely packed with GVD bodies (B4-6, C2'-C4'), while in other cases it displays the neuronal morphology resembling pTau positive neurons with tangles (B-B3, C-C4, as pointed by arrowheads).

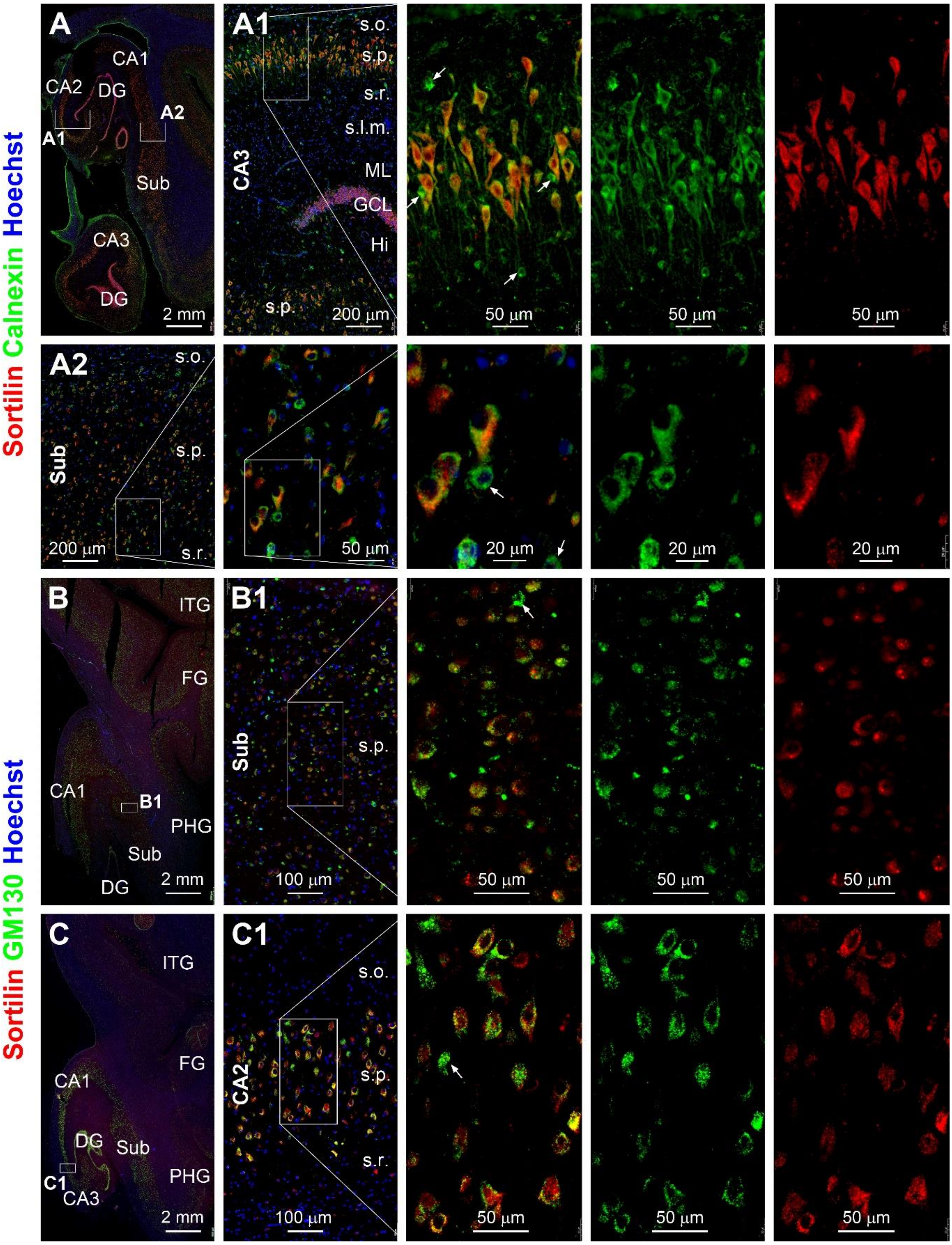

Supplemental Figure 27. Differential colocalization of sortilin with the endosomal marker calnexin and the Golgi body marker GM130 (A from case #18; B from case #30; C from case #39). There is a partial colocalization for the two pairs of markers among the hippocampal and subicular pyramidal neurons as evidenced by the presence of yellow areas inside the somata and proximal dendrites in the merged image panels. It should be noted that calnexin and GM130 labeling are also present in relatively small-sized cells, likely interneurons, wherein the sortilin labeling is weak or absent (examples are pointed by arrows).

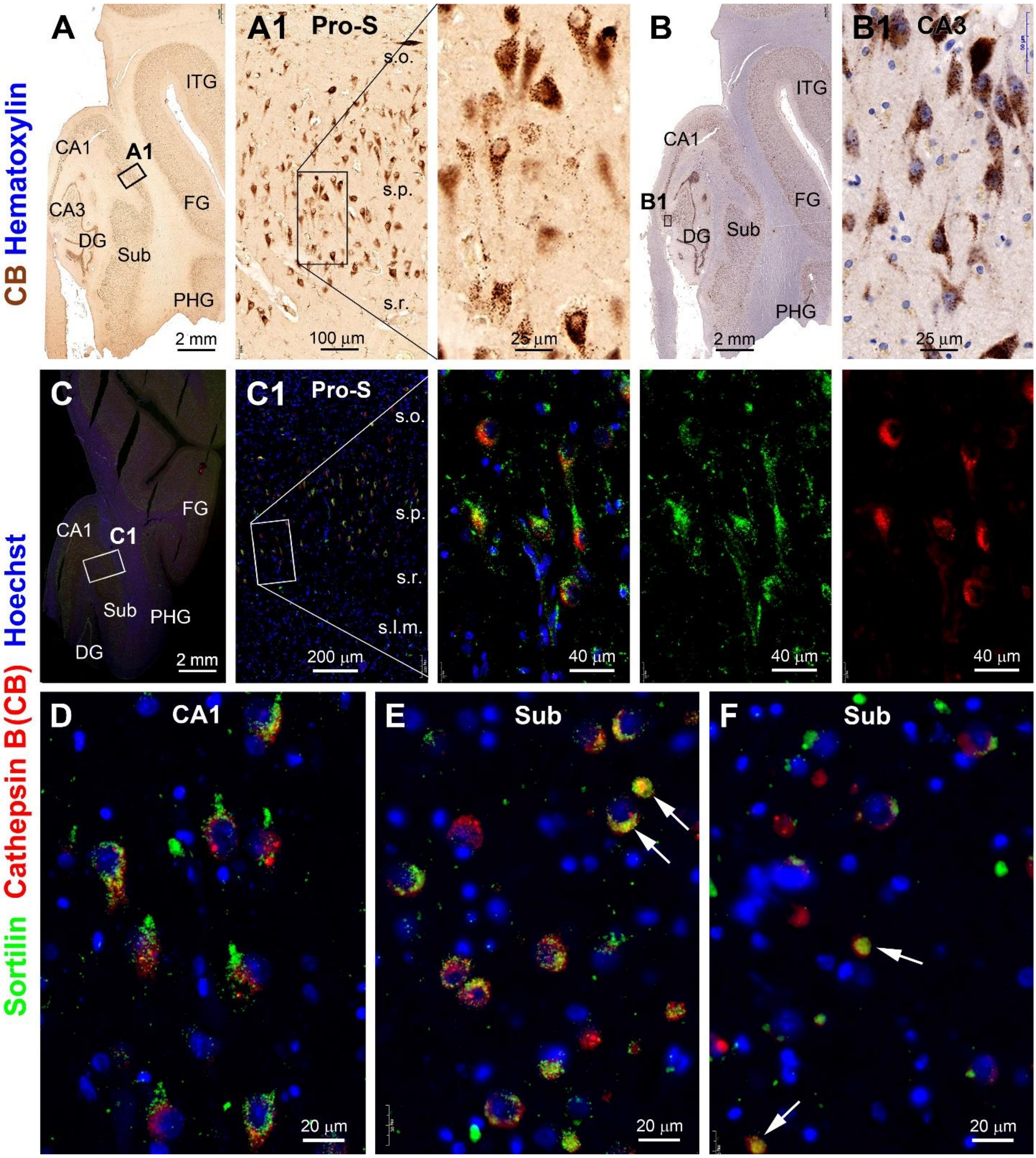

Supplemental Figure 28. Partial colocalization of sortilin with the lysosomal marker cathepsin B (CB) in hippocampal/subicular pyramidal neurons in paraffin sections from case #44 (from the pAD/AD group). Panels (A, A1) and (B, B1) are low power and enlarged views of CB immunolabeling with the peroxidase-DAB method. CB labeling appears granule-like in the somata and proximal dendrites. In double immunofluorescence (C, D and enlargements), there exists a partial colocalization of the two markers, as indicated by the yellow areas inside the neurons the merged image panels, including at some aggregation bodies (pointed by arrows). In some neurons, the intraneuronal areas appear greener or redder in the merged panels, which implicates that the distributions of two markers can be compartmented inside neurons.

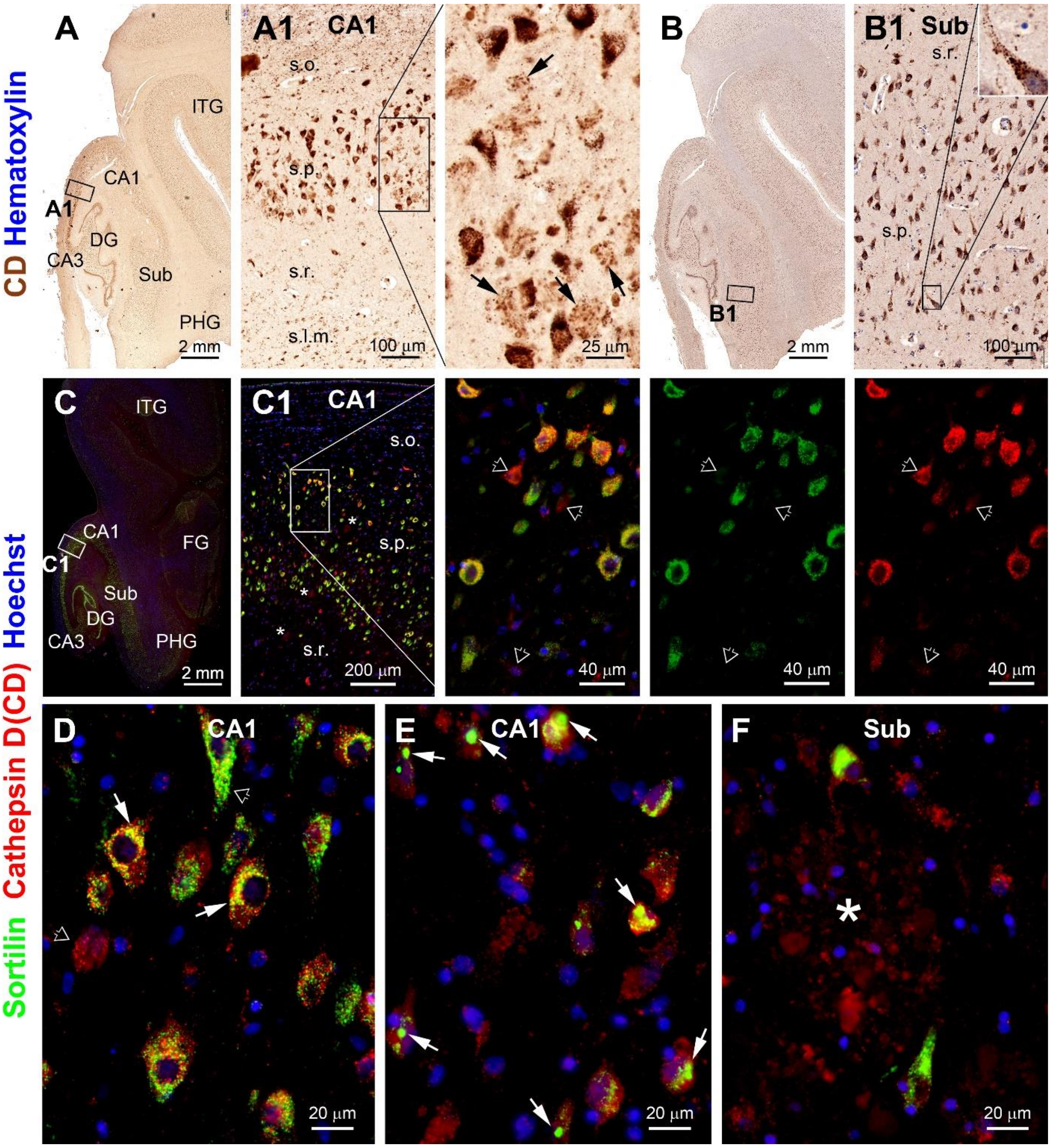

Supplemental Figure 29. Partial colocalization of sortilin with the lysosomal marker cathepsin D (CD) in hippocampal/subicular pyramidal neurons. The paraffin sections were from case #44 in the pAD/AD group. Panels (A, A1) and (B, B1) are low power and enlarged views of CD immunolabeling with the peroxidase-DAB method. CD labeling appears granule-like in the somata and proximal dendrites, and also occurs in clusters of dystrophic neurites (A1 and enlargements, pointed by arrows). In double immunofluorescence (C-F and enlargements), there exists a partial colocalization of the two markers, which appears as yellow areas inside the neurons, including at some aggregation bodies (pointed by arrows). This partial colocalization indicates that the expression of two proteins is somewhat compartmented in neurons. Notably, some neurons with heavy CD labeling exhibits reduced or no sortilin labeling (pointed by open arrows). It should be also noted that the dystrophic neurites (*) are clearly labeled for CD, whereas no sortilin labeling is present in these swollen neurites (F).

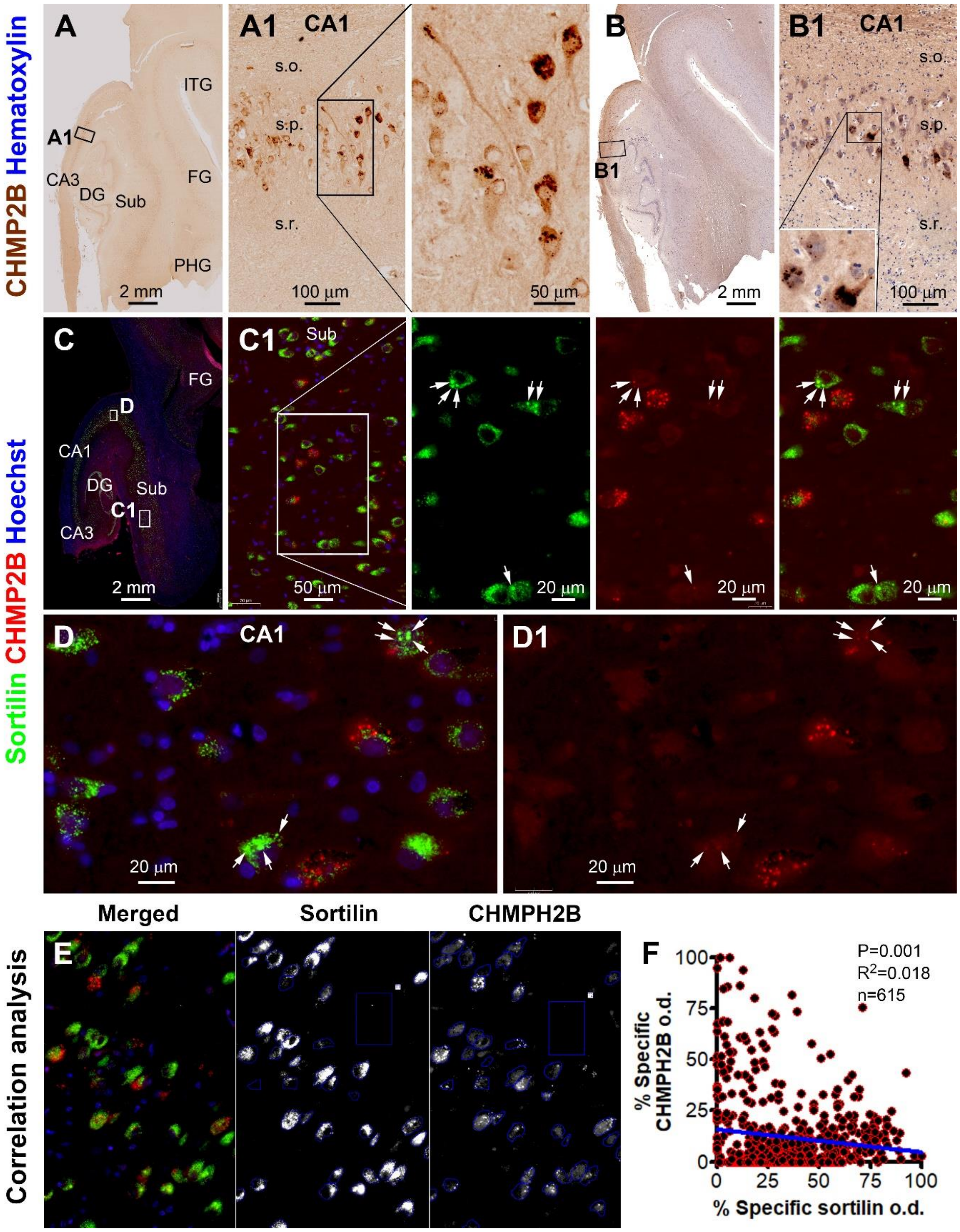

Supplemental Figure 30. Differential colocalization of the charged multivescular body protein 2B (CHMPH2B) and sortilin in hippocampal/subicular pyramidal neurons (case #44). (A-B1): DAB-immunolabeling of CHMPH2B; noting the cytosol labeling in some neurons. There is a partial colocalization CHMPH2B and sortilin at the aggregation bodies (pointed by arrows). Among the co-labeled neurons, sortilin labeling tends to reduce as the number of CHMPH2B granules increases (C-D1). Panel (E) illustrates the methodology for single-cell densitometry as described in main Figure 5. There exists a significant inverse correlation between CHMPH2B and sortilin immunoreactivity among individual labeled neurons (F).
